# Candidate Glaucoma Biomarkers: From Proteins to Metabolites, and the Pitfalls to Clinical Applications

**DOI:** 10.3390/biology10080763

**Published:** 2021-08-10

**Authors:** Andrés Fernández-Vega Cueto, Lydia Álvarez, Montserrat García, Ana Álvarez-Barrios, Enol Artime, Luis Fernández-Vega Cueto, Miguel Coca-Prados, Héctor González-Iglesias

**Affiliations:** 1 Instituto Oftalmológico Fernández-Vega, Avda. Dres. Fernández-Vega, 34, 33012 Oviedo, Spain; afvega89@gmail.com (A.F.-V.C.); mgarciadiaz@fio.as (M.G.); 2 Instituto Universitario Fernández-Vega, Fundación de Investigación Oftalmológica, Universidad de Oviedo, 33012 Oviedo, Spain; l.alvarez@fio.as (L.Á.); ana.alvarezbarrios1@gmail.com (A.Á.-B.); enol.artime@fio.as (E.A.); 3 Department of Ophthalmology and Visual Science, Yale University School of Medicine, New Haven, CT 06510, USA; miguel.coca-prados@yale.edu

**Keywords:** glaucoma, biomarkers, proteomics, metabolomics, early diagnosis, ocular fluids, eye tissues, blood serum, omics sciences

## Abstract

**Simple Summary:**

Glaucoma is a devastating eye disease causing progressive vision loss and consequent irreversible blindness. The global prevalence of glaucoma is estimated at 80 million people, with a projected increase in the number of people affected to 112 million by 2040. The clinical diagnosis of glaucoma usually occurs late, by which time up to 40% of neurosensory cells may be lost. There is an overriding need for early diagnosis systems based on the analysis of glaucoma biomarkers. However, plenty of candidate biomarkers have been published to date in humans, without clear clinical translation. In this review, we have summarized the efforts carried out for the discovery of proteomics- and metabolomics-based glaucoma biomarkers in blood, aqueous humor, tears, and ocular tissues from human subjects. The huge amount of data without real clinical application merits a new integrative approach, allowing future diagnostic tests to be based on local and/or systemic biomarkers of glaucoma.

**Abstract:**

Glaucoma is an insidious group of eye diseases causing degeneration of the optic nerve, progressive loss of vision, and irreversible blindness. The number of people affected by glaucoma is estimated at 80 million in 2021, with 3.5% prevalence in people aged 40–80. The main biomarker and risk factor for the onset and progression of glaucoma is the elevation of intraocular pressure. However, when glaucoma is diagnosed, the level of retinal ganglion cell death usually amounts to 30–40%; hence, the urgent need for its early diagnosis. Molecular biomarkers of glaucoma, from proteins to metabolites, may be helpful as indicators of pathogenic processes observed during the disease’s onset. The discovery of human glaucoma biomarkers is hampered by major limitations, including whether medications are influencing the expression of molecules in bodily fluids, or whether tests to validate glaucoma biomarker candidates should include human subjects with different types and stages of the disease, as well as patients with other ocular and neurodegenerative diseases. Moreover, the proper selection of the biofluid or tissue, as well as the analytical platform, should be mandatory. In this review, we have summarized current knowledge concerning proteomics- and metabolomics-based glaucoma biomarkers, with specificity to human eye tissue and fluid, as well the analytical approach and the main results obtained. The complex data published to date, which include at least 458 different molecules altered in human glaucoma, merit a new, integrative approach allowing for future diagnostic tests based on the absolute quantification of local and/or systemic biomarkers of glaucoma.

## 1. Introducing Glaucoma

**Figure 1:**
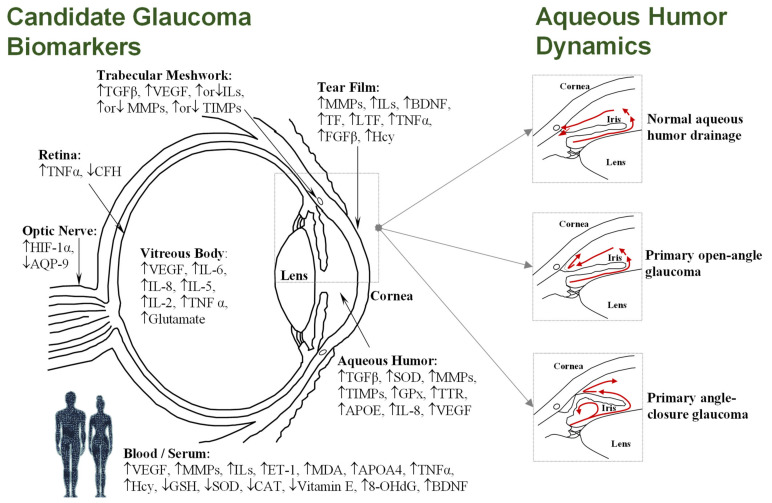
(**Left**): Diagram of the human eye in horizontal section showing the main structures and the most frequently cited biomarkers identified in the different tissues and/or fluids (see Tables 1–4); Upward-pointing arrows indicate higher levels in glaucoma; Downward-pointing arrows indicate lower levels in glaucoma; Simultaneous up-ward or downward-pointing arrows for ILs, MMPs, and TIMPs indicate higher or lower levels in glaucoma, depending on the isoform. (**Right**): Simplified aqueous humor dynamics in a healthy eye, where aqueous humor mainly exits through the trabecular meshwork and uveoscleral route (up); during primary open-angle glaucoma, an obstruction occurs in the trabecular meshwork, increasing the resistance to aqueous humor drainage (middle); physical blockage of the inner surface of the chamber by the iris occurs in angle-closure glaucoma, with consequent obstruction of drainage pathways (down).

Glaucoma, the leading cause of irreversible blindness worldwide, encompasses a complex group of neurodegenerative ocular disorders characterized by the progressive degeneration of the optic nerve, retinal ganglion cell (RGC) death, and the loss of visual field [1]. Global glaucoma prevalence is currently estimated at 80 million people, with 3.5% incidence in people aged 40–80, while the number of people affected is projected to increase to 112 million by 2040 [2,3]. Although age and genetic background are risk factors for glaucoma, elevated intraocular pressure (IOP) is the leading risk factor for most types of glaucoma. The IOP level (normal range 10–21 mm Hg) is dependent on the rate of the removal of aqueous humor fluid, which is secreted by the ciliary epithelium, and flows to the anterior chamber through the trabecular meshwork and into Schlemm’s canal, before draining into the venous system. However, as shown Figure 1, imbalance in the aqueous outflow produces elevated IOP due to fluid accumulation, leading to ischemic infarcts in retinal and optic nerve head lesions [4,5].

Glaucoma is mainly classified into open-angle or angle-closure subtypes, both of which can subsequently be further subdivided into primary or secondary disease. Primary refers to the presence of the characteristic neuropathy of glaucoma, related to a normal or elevated IOP without known pathological cause. Meanwhile, secondary refers to an elevated IOP with a recognizable pathological cause, such as trauma, neovascularization, pigment dispersion, inflammation, or pseudoexfoliation. In addition, glaucoma is clinically classified as acute or chronic, based on its duration [6]. The clinical classification of glaucoma results in different subtypes. Primary glaucomas include primary open-angle glaucoma (POAG), normal-tension glaucoma (NTG), high-tension glaucoma (HTG), primary angle-closure glaucoma (PACG), and primary congenital glaucoma (PCG). Secondary glaucomas include pseudoexfoliation glaucoma (PEXG), neovascular glaucoma (NVG), secondary open-angle glaucoma (SOAG), secondary angle-closure glaucoma (SACG), and glaucoma secondary to pigment-dispersion syndrome (SGPDS), among others. Moreover, acute or chronic glaucomas comprise, among the angle-closure glaucomas (ACG), the chronic angle-closure glaucoma (CACG) and the acute angle-closure glaucoma (AACG), while among the narrow-angle glaucomas (NAG), they comprise the chronic narrow-angle glaucoma (CNAG) and the acute narrow-angle glaucoma (ANAG) [6].

Among all of the subtypes of glaucoma resulting in irreversible blindness, POAG and PEXG are the most prevalent in developed countries [7,8]; both of these types of glaucoma are multifactorial in origin, sharing an abnormal increase in the IOP related to their onset and progression. The IOP elevation is associated with a dysfunction of the normal flow of the aqueous humor, either because of an excessive production of this fluid, or due to the obstruction of the outflow system triggered by an accumulation of aggregates in the trabecular meshwork (see Figure 1) [9]. POAG, comprising nearly three-quarters of all glaucoma cases, is characterized anatomically by an open angle in the junction of the cornea and the iris, where the aqueous humor of the eye drains out of the anterior chamber into the venous system. In spite of the fact that the angle is open, the drainage of aqueous humor is obstructed in POAG. The IOP elevation usually occurs gradually, and symptoms are typically unnoticed by the patient until alterations in the visual field and damage to the optic nerve are detected in clinical eye exams [7]. PEXG is a secondary open-angle glaucoma, with elevated IOP being the manifestation of the exfoliation syndrome (PES) in the eye [10]. An excessive production of abnormal extracellular material, which peels off the outer layer of the lens, progressively accumulates in the angle of the anterior chamber and clogs the drainage system, causing an increase in the IOP levels. The progression of PEXG is faster than that of POAG, and has a worse prognosis in terms of visual field and optic nerve damage [11].

In PACG, the peripheral iris is in contact with the trabecular meshwork and the peripheral cornea, physically blocking the anterior chamber, reducing fluid drainage, and causing increased IOP (see Figure 1). This can be more acute in onset than open-angle glaucoma, and could occur gradually (chronic angle-closure glaucoma) or suddenly (acute angle-closure glaucoma). Ultimately, IOP rises quickly, causing symptoms such as blurry vision, eye pain, headache, redness, rainbow-colored rings (“haloes”) around light, and nausea and/or vomiting requiring immediate medical attention. Risk factors for angle-closure glaucoma include female gender, older age, Asian ethnicity, and having a small eye with a crowded anterior segment, a small central anterior chamber depth, a thicker and more anteriorly positioned lens, or short axial length of the eye [4]. Conversely, NTG shows glaucomatous optic neuropathy and visual field loss without IOP elevation. Advanced age, female sex, Asian ethnicity, low central corneal thickness, vascular dysfunction, ischemia episodes, Raynaud‘s phenomenon, migraine, smoking, high body mass index, hypertension, impaired glucose tolerance, nocturnal systemic hypotension, and overtreated systemic hypertension are among the most important risk factors for NTG [12].

Glaucoma diagnosis, classification, treatment response evaluation, and progression monitoring require the combination of clinical exam, IOP measurements, and the interpretation of visual field and structural imaging parameters. During the early stages of glaucoma, screening techniques based on IOP measurements provide unsuitable sensitivity, especially in those patients with values within the normal range. In addition, cup-to-disc ratio does not provide enough sensitivity and specificity for predicting glaucoma [13]. Above all, it is often clinically observed that by the time glaucoma is diagnosed, the patient has already lost 35–40% of their RGCs [14]. Therefore, there is a current need for a much more sensitive and specific method of early diagnosis of glaucoma, contributing to the detection of disease progression and improvement of its prognosis and monitoring treatment response.

## 2. Molecular Biomarkers

Molecular biomarkers of glaucoma are potentially beneficial in the early diagnosis and management of this eye disease, leading to a better understanding of its pathophysiology [15]. A biomarker is usually defined as a “characteristic that is objectively measured and evaluated as an indicator of normal biological processes, pathogenic processes, or pharmacologic responses to a therapeutic intervention” [16]. The term molecular biomarker refers to all biomarkers that are measurable by methods based on the marker’s molecular properties. This definition would comprise a wide range of biomarkers, from small to large molecules, from nucleic acids to proteins [17]. Ideal characteristics for proper biomarkers include being an agent of measureable entity, with high sensitivity and specificity, which accurately predicts the presence, progression, or absence of a disease.

In 2011, the seventh annual ARVO/Pfizer Ophthalmic Research Institute conference revised the state of knowledge of molecular biomarkers associated with glaucoma, including cutting-edge techniques for their identification and validation [18]. Over the past 10 years, substantial progress has been made, although clinical applications for the early diagnosis of glaucoma are absent to date [19,20]. When looking for molecular biomarkers, the primary neurodegeneration of glaucoma occurs in the optic nerve and the inner retina, although these tissues remain readily inaccessible to biopsy. In contrast, the aqueous humor, vitreous body, and especially the tear film are more accessible, but distant from the degenerative place, let alone serum. To date, most of the studies aimed at the discovery of molecular biomarkers for clinical application have been focused on detecting the molecular and cellular mechanisms involved in the neuronal injury of RGCs in bodily fluids and tissues, including the tear film, aqueous humor, vitreous body, and blood/serum [15,21].

The discovery and implementation of glaucoma biomarkers are hampered by major difficulties. Glaucoma medication may affect the expression of candidate biomarkers in bodily fluids—especially those in contact with topically used drugs, such as tears or aqueous humor. Moreover, tests to validate glaucoma biomarker candidates should include subjects with different types and stages of glaucoma, as well as patients with other ocular and neurodegenerative diseases. In addition, interindividual variations in glaucoma patients, as well as the great existing dynamic range of proteins to metabolite concentrations, the technical irreproducibility in the analysis and absence of proper quality assurance contribute to limit the reliability of candidate markers. Therefore, cutting-edge methods for diagnosing different types of glaucoma, based on the quantitative analysis of molecules in bodily fluids—preferable non-invasive—are currently required [22].

Undoubtedly, molecular biomarkers of glaucoma are perceptive for the early diagnosis, the risk profile for optic neuropathy progression, the early detection of damage progression in diagnosed patients, and in monitoring responses to treatment. Identification of clinically applicable molecular biomarkers is an area of active investigation, and omics disciplines are essential in ongoing studies. Many proteomics and metabolomics works have been carried out to identify altered molecules in glaucoma patients compared to control individuals with or without age-related cataracts, obtaining a large number of dysregulated species in different biological tissues and fluids, including tear film, aqueous humor, trabecular meshwork cells, and blood (serum) [23,24,25,26,27,28,29,30,31,32,33,34,35,36,37,38]. The high numbers of studies have provided hundreds of proteins and metabolites (>450) suggestive of association with the disease, but whether any of the candidate biomarkers proposed is the cause or consequence of glaucoma remains a matter of debate.

However, all of the current data contribute to shed light into the proteome, metabolome, and so on in the different subtypes of glaucoma, within and without the eye, providing a global perspective of all biochemical processes occurring in an organism. Accordingly, although it is virtually impossible to report all of the molecules that have been proposed as candidate biomarkers of glaucoma during recent decades, we attempted to summarize the current literature. In this review, we present an overview of identified biomarkers—i.e., proteins or metabolites—in different fluids and tissues from glaucoma patients that have been replicated or discussed by others, or validated in an additional cohort. The analytical platform and the published fold change related to the up- or downregulation of the protein or metabolite altered are also indicated, along with the fluid or tissue used for the discovery. In addition, the Supplementary Materials include all of the listed studies and the significantly altered proteins or metabolites identified in eye tissues or bodily fluids to date. Moreover, Figure 1 depicts a simplified diagram of the human eye, in horizontal section, showing the main structures and the promising candidate biomarkers identified so far in each specific biofluid and/or eye tissue, which will be extensively discussed in the following sections.

## 3. Candidate Molecular Biomarkers Identified in Eye Fluids, Eye Tissues, and Blood/Sera

### 3.1. Aqueous Humor

The aqueous humor is a colorless intraocular fluid produced by the pigmented and non-pigmented ciliary epithelium; it is derived from the plasma, within the capillary network of the ciliary body, secreted at a rate of 2–2.5 μL/min into the posterior chamber, and circulating through the pupil into the anterior chamber. Therefore, this intraocular fluid contains proteins from the anterior segment structures and serum, being responsible for the supply of nutrients and removal of metabolic wastes from the avascular tissues, as well as for distributing signaling molecules [9,39]. Considering that protein and/or metabolite content in aqueous humor is modified during glaucoma, this biofluid may contain markers directly related to RGC neurodegeneration, inflammation, immune response, oxidative stress, or apoptosis. However, because sampling the aqueous humor is invasive, and is generally limited to patients undergoing an intraocular surgery, inherent limitations must be considered for routine analysis. Most of the studies aiming at the discovery of candidate biomarkers in aqueous humor address its proteome and, to a lesser extent, metabolome profiles, as shown in Table S1 of the Supplementary Materials. Particularly, Table 1 contains the most representative studies published so far in aqueous humor, describing candidate markers that have been replicated by others.

#### 3.1.1. Protein-Based Biomarkers

• Inflammatory-related markers

One of the seminal works identified in the literature used enzyme-linked immunosorbent assay (ELISA) to determine the presence of altered levels of transforming growth factor-beta 2 (TGF-β2) in samples of aqueous humor from 15 patients with POAG and 10 age-matched control subjects undergoing cataract surgery, constituting one of the first attempts to discover candidate biomarkers of glaucoma [40]. In fact, the aqueous humor from POAG patients had a statistically significantly greater amount of total and active TGF-β2 (1.8-fold). Similarly, this observation was confirmed in 43 Korean patients with POAG, NVG, or SOAG, and 20 controls [41], as well as in NVG subjects of Chinese origin, where TGF-β2 and transforming growth factor-beta 1 (TGF-β1), quantified by ELISA, were observed to be upregulated (see Table 1) [42], supporting the hypothesis of the intraocular derivation of this cytokine. Moreover, a cytotoxic protein to the trabecular meshwork and RGCs—the soluble CD44 (sCD44), interacting with TGF-β—was also found to be increased in POAG aqueous humor (2.2-fold vs. controls), which also correlates with the severity of visual field loss [43]. This observation related to sCD44 was later confirmed, in addition to erythropoietin (EPO) overexpression, in the aqueous humor of POAG patients, although no significant differences were found in plasma [44]. The proinflammatory cytokine tumor necrosis factor alpha (TNF-α), analyzed by singleplex immunoassay, was found to be increased (1.7-fold) in the aqueous humor of 32 POAG patients when compared with the same number of controls, suggesting TNF-α to be a reliable biomarker in the progression of glaucoma [45]. Later, a multiplex-based immunoassay carried out in a Japanese population, consisting of 20 POAG, 23 PEXG, and 21 control patients, revealed significantly higher levels of TGF-β1, interleukin (IL)-8, and serum amyloid A (SAA) in the aqueous humor of POAG (5.0-fold, 2.3-fold, and 11.9-fold, respectively) and PEXG (12.5-fold, 4.0-fold, and 18.3-fold, respectively) patients, and lower levels of IL-6 for POAG subjects, when compared with control subjects [46].

Similarly, the analysis of a cytokine expression panel in the aqueous humor of PEXG, PES, and control subjects, via a multiplex immunoassay platform, confirmed the proinflammatory environment in this subtype of glaucoma, detecting higher levels of the chemokines (CXCLs) CXCL13, CCL24, CCL13, CCL22, CCL15, and CXCL16, the proinflammatory cytokine IL-16, and IL-4 [47]. Recently, the level of vascular endothelial growth factor A (VEGF-A) was found to be upregulated in the aqueous humor of NVG patients when compared with proliferative diabetic retinopathy subjects (1.2-fold), indicating specific glaucomatous inflammation, different to secondary retinopathy. Conversely, no differences were observed for VEGF-A in the vitreous body, nor for the molecules ILs, placental growth factor (PGF), or EPO [48]. The implication of cytokines in glaucoma was further explored by determining the levels of TGF-β2 and secreted frizzled-related protein 1 (SFRP1) in the aqueous humor obtained from 126 eyes with different types of glaucoma (POAG, CACG, primary angle-closure suspects (PACS), and AACG), via conventional ELISA. Only the concentration of TGF-β2 in POAG patients was significantly higher than in control subjects, although TGF-β2 in AACG patients with high IOP (> 21 mmHg) was significantly higher than those with normal IOP. The SFRP1 levels were lower in POAG patients when compared with normal IOP subjects, while AACG patients with high IOP had a higher level of SFRP1 than those with normal IOP. Therefore, it seems that there exists a specific and very complex pattern of proinflammatory cytokine expression depending on the type of glaucoma and the IOP [49]. Recently, the aqueous autotaxin (ATX) and TGF-β levels were determined via enzymatic or multiplex immunoassay in the aqueous humor of a large cohort consisting of 281 subjects, including POAG (n = 97), SOAG (n = 48), PEXG (n = 48), and controls (n = 88). Both ATX and TGF-β1 concentrations were higher in glaucoma patients than control subjects, exhibiting acceptable diagnostic performance in detecting disease subtypes [50].

This altered inflammatory environment was further explored in comparison with other diseases affecting the eye. Ten Berge et al., in 2019 [51], analyzed a panel of cytokines—including IL-1β, IL-1ra, IL-2, IL-6, IL-6rα, IL-7, IL-8, IL-10, IL-17A, IL-23, thymus- and activation-regulated chemokine (TARC), monocyte chemoattractant protein-1 (MCP-1), TNF-α, PGF, and VEGF—in patients with glaucoma, age-related macular degeneration (AMD), retinitis pigmentosa (RP), and cataracts, using a multiplex assay. Interestingly, only IL-8 concentrations were higher in the aqueous humor of glaucoma and AMD patients, when compared to control subjects, while increasing age was associated with higher levels of intraocular cytokines and, therefore, future studies should be controlled for the age of patients. Similarly, IL-8 levels were also upregulated, along with VEGF-A and EPO, in the aqueous humor of patients diagnosed with stable NVG, when compared with control subjects and additional patients with some retinal ischemic conditions [52].

Growth differentiation factor 15 (GDF15), a member of the TGF-β superfamily, was found to be increased in the aqueous humor of POAG patients, and also significantly associated with worse functional outcomes in individuals with POAG and PEXG, being therefore proposed as marker of glaucoma severity that may be generalizable to multiple types of glaucoma, regardless of the underlying etiology [53,54]. Furthermore, endothelin (ET) levels, related to inflammation, were quantified in the aqueous humor and plasma of POAG (n = 31) and control (n = 24) subjects by radioimmunoassay (RIA), observing limited significant upregulation (1.05-fold) of ET in the aqueous humor of POAG patients (44.26 + 2.6 pg·mL^−1^) when compared to controls (42.17 + 1.6 pg·mL^−1^), while no significant differences were observed in plasma [55]. Later, endothelin-1 (ET-1) was quantified by ELISA, along with klotho—a newly discovered protein that presumably plays an important role in the aging process—in the serum and aqueous humor of patients with PES and PEXG. Similarly, ET-1 levels were significantly higher while klotho levels were lower in both the PES and PEXG groups when compared to controls [56]. Moreover, the levels of the brain natriuretic peptide (BNP) and atrial natriuretic peptide (ANP), suggestively regulated by proinflammatory cytokines, were measured by RIA in the aqueous humor of glaucoma patients (41 POAG, 3 PEXG, and 3 CNAG) and control subjects (n = 47), although no significant differences were found [57]. Due to the suggested role of natriuretic peptides in maintaining the neural and vascular integrity of the mature retina and the optic nerve, along with their possible involvement in the regulation of IOP and the development of glaucoma, the concentration of the N-terminal fragment of the proatrial natriuretic peptide (NT-proANP) was determined via ELISA in the aqueous humor and plasma of POAG (n = 58) and control (n = 32) subjects, showing significantly higher levels in both fluids of glaucoma patients [58]. Similarly, biomarkers of inflammatory neurodegeneration have been studied in the aqueous humor of POAG patients’ eyes in search of a link between neurodegenerative processes and trabecular meshwork injury, using multiplex immunoassays to identify the overexpression of the cytokines cathepsin D (CD, 1.2-fold), soluble neural cell adhesion molecule (sNCAM, 1.3-fold) and soluble vascular cell adhesion molecule-1 (sVCAM-1, 1.9-fold) in glaucoma patients [59].

Considering that topical treatments with antiglaucomatous drugs may contribute to local inflammation, a combined study by Burgos-Blasco et al. in 2020 [60] evaluated the concentrations of proinflammatory cytokines in both the tears and aqueous humor of topically treated POAG patients (n = 27) and controls (n = 27), using multiplexed immunoassay; while IL-5, IL-12, IL-15, interferon (IFN-γ), and macrophage inflammatory protein (MIP-1α) levels were significantly higher in the aqueous humor of eyes with glaucoma, an increase in IL-4, IL-12, IL-15, basic fibroblast growth factor (FGF-β), and VEGF, along with a decrease in MIP-1α, was observed in tear samples from POAG patients. Therefore, a poor correlation exists between cytokine levels in tears and aqueous humor, sharing only IL-15 overexpression, along with a distinct pattern for MIP-1α, and their concentrations may be poorly affected by topical treatment, supporting the role of glaucoma as an inflammatory pathology.

• Oxidative stress-related markers

In the early 2000s, studies focused on the role of oxidative stress in glaucoma began to emerge, with the analysis of nitric oxides and cyclic guanosine monophosphate (cGMP). While the quantitation of nitrate, nitrite, and cGMP in the aqueous humor and serum of glaucomatous and control Finnish subjects by spectrophotometry, chemiluminescence, or RIA methods showed no statistically significant differences, the levels of cGMP and nitrite in Italian POAG patients were lower than in the control subjects (see Table S1 of the Supplementary Materials) [61,62]. Furthermore, the activity of superoxide dismutase (SOD) and glutathione peroxidase (GPx) was found to be increased in POAG patients when compared with cataractous control patients, stating that despite some conflicting results, oxidative stress during glaucoma leads to an induction of antioxidant enzymes, these being candidate stress markers in the aqueous humor of glaucoma patients [63]. This hot topic has been widely explored throughout the first two decades of this century, with different approaches. For example, protein carbonyl, providing protein oxidation as an oxidative stress marker, was found to be upregulated in the aqueous humor and serum of PEX patients (n = 29) compared to controls (n = 27) [64]. Similarly, the pro-oxidant–antioxidant balance (PAB) and the hydrogen peroxide levels were significantly upregulated in both the aqueous humor and serum of PEXG patients, while the catalase (CAT) activity was found to be lower in PEXG and PES patients when compared to control individuals [65].

The activity of well-known antioxidant enzymes was also evaluated in the aqueous humor of POAG subjects, showing increased levels of GPx (2.9-fold), SOD (1.8-fold), and malondialdehyde (MDA, 8-fold) when compared with patients with cataracts [66]. Later, the use of antibody microarrays permitted the quantification of oxidative-stress-related proteins in 10 POAG and 10 control patients, showing lower SOD and glutathione transferase (GST), along with higher glutamine synthase (GS) and nitric oxide synthase (NOS), in the aqueous humor of glaucoma patients, leading to the hypothesis that the reduced expression of the antioxidant enzymes SOD and GST could aggravate the imbalance between the production and detoxification of oxygen- and nitrogen-derived free radicals [67]. The observed difference in levels or activities of antioxidants in glaucoma is a common and limiting issue. Goyal et al., in a spectrophotometric targeted analysis in 2011 [68], observed a significant increase in SOD and GPx activity in both POAG (n = 30) and PACG (n = 30) aqueous humor compared to patients with cataracts (n = 30), while vitamins C and E were significantly lower in glaucoma subjects. Hondur et al., in 2017 [69], explored the usefulness of oxidative-stress-related biomarkers—i.e., protein carbonyls and advanced glycation end-products (AGEs)—to discriminate POAG (n = 96) and control (n = 64) subjects. ELISA-based quantification showed aqueous humor and blood levels of protein carbonyls and AGEs to be significantly higher in glaucomatous samples, which may be promising for clinical predictions. Overall, oxidative stress may play a role in the pathogenesis of glaucoma, which might lead to the induction of antioxidant enzymes and contribute to decreased reactive antioxidant potential.

• Extracellular-matrix-related markers

The suggested role of altered extracellular matrix metabolism in glaucoma fostered the analysis of matrix metalloproteinases (MMPs) and their tissue inhibitors (TIMPs) in aqueous humor samples from POAG, PES, PEXG, and cataract control patients. The TIMP-2 levels were significantly elevated in both glaucoma (POAG and PEXG) and PES subjects, while MMP-2 was higher in PEXG and PES subjects, when compared to samples from controls [70]. Furthermore, the concentrations of cellular and plasma fibronectin (FN)—present in the extracellular matrix and determined by ELISA in PEXG, POAG, and control subjects—were higher in PEXG when compared with the other groups, which may be a consequence of disruption of the blood–aqueous barrier [71]. Conversely, lower levels of hyaluronic acid were detected in POAG patients when compared to control subjects [72]. Considering that the overproduction and accumulation of abnormal matrix components in PEXG mediated by MMPs can exert inflammatory and immunological reactions via various chemokines, cytokines, growth factors, and cell-surface receptors, MMP-2, connective tissue growth factor (CTGF), and TIMP-2 were quantified in the aqueous humor by ELISA. Increased levels of MMP-2, CTGF, and TIMP-2 were observed in 60 glaucoma patients (30 PEXG and 30 POAG) when compared to controls, which may involve changes during the pathogenesis of the disease [73]. On the other hand, the study of gelatinase A activity by zymography revealed significant differences in POAG subjects (3.9-fold, when compared to control subjects) [74].

Matricellular proteins—a group of nonstructural modular extracellular proteins—are widely expressed in eyes with glaucoma, suggesting that they may play an important role in the pathogenesis of the disease. Thereby, the levels of CTGF—a matricellular protein that may play a role in the deposition of elastic microfibrillar exfoliation material, and interacts with the cytokine TGF-β—determined by ELISA were significantly higher in the aqueous humor of patients with PEXG than that of both POAG and control subjects [75]. CTGF was recently evaluated again as a candidate biomarker of PEXG, but its concentration in aqueous humor did not differ between PEXG, PES, and control patients, while the CTGF and total protein contents in tear fluid were significantly higher in PEXG cases compared to controls. However, CTGF was not a good predictor for PEXG or PES in tears or aqueous humor, showing again the high variability and lack of reliability of the proposed candidate biomarkers [76]. Moreover, the concentrations of secreted protein acidic and rich in cysteine (SPARC), tenascin-C (TNC), thrombospondin-2 (TSP-2), and osteopontin (OPN) were determined by multiplexed immunoassays in patients diagnosed with PACG (n = 41) and controls (n = 22), showing significantly elevated matricellular protein levels in glaucoma patients [77]. A liquid chromatography tandem mass spectrometry (LC–MS/MS) proteomics analysis carried out in the aqueous humor of POAG (n = 90), PACG (n = 72), and control (n = 78) subjects showed the under-regulation of the extracellular matrix proteins OPN, CD, and cystatin C (CysC), which was further validated by Western blot (WB), with the exception of CD [78]. Recently, the levels of fibulin (FBLN)-7—an adhesion molecule that interacts with extracellular matrix molecules involved in the maintenance of the trabecular meshwork’s functionality—were found to be downregulated in the aqueous humor of PACG patients when compared with POAG and control subjects [79]. The same group also determined the concentration of clusterin (CLU) in the aqueous humor and tears of POAG, PEXG, and control subjects via ELISA, observing markedly higher aqueous humor CLU levels in patients with PEXG [80]. Finally, the matricellular proteins periostin (PN) and TNC were studied, in a target analysis, in the aqueous humor of NVG patients, obtaining significantly higher levels when comparing glaucoma patients with subjects diagnosed with proliferative diabetic retinopathy. Interestingly, significant expression of PN in the trabecular meshwork and Schlemm’s canal of patients with NVG was observed [81].

• Immune-response-, neurodegeneration-, and apoptosis-related markers

Growing evidence of an immunological component in the pathogenesis of glaucoma fostered the study of antibody patterns in the aqueous humor [82]. Joachim et al. found significant differences in the antibody profiles of glaucoma patients, observing upregulation of heat shock protein (HSP)70 and vimentin (VIM) in NTG subjects [83], in addition to HSP27 overexpression and α-enolase (ENO1), actin, and glyceraldehyde-3-phosphate dehydrogenase (GAPDH) downregulation in both POAG and PEXG patients, when compared to controls [84]. These differences in antibody patterns constitute further evidence of an autoimmune involvement in the pathogenesis of some glaucoma patients. In this line, in a recent work, the use of machine learning algorithms with data from 28 immune-mediator levels obtained from the aqueous humor of patients diagnosed with different ocular diseases—including POAG—provided higher POAG prediction (area under the curve (AUC) = 0.90), especially when using the markers MCP-1, IL-6, and angiogenin [85], although IL-6 was not consistent according to Takai et al. [46].

Earlier studies have applied, in an untargeted approach, differential proteomics to study aqueous humor samples from POAG patients (n = 52), identifying transthyretin (TTR) as a potential candidate biomarker, when compared to control subjects (n = 55) [86]. Specifically, in a first stage, SELDI-TOF-MS was conducted on 22 POAG and 24 control samples, while 2D electrophoresis was applied to 33 POAG and 31 control subjects, identifying by means of LC–MS/MS the upregulation of TTR (1.9-fold), further confirmed in a second stage by ELISA. This protein might play a role in the pathogenesis of glaucoma, causing amyloid deposition in the trabecular meshwork, which contributes to a mechanical barrier to aqueous fluid outflow that could consequently lead to this eye disease in some patients. Interestingly, a proteomic analysis based on 2D electrophoresis and LC–MS/MS, and carried out in five POAG patients with uncontrolled IOP—despite the use of well-tolerated medical therapy—and five control subjects showed significant upregulation of TTR, as well as prostaglandin H2 D-isomerase (PTGDS, validated by WB), caspase 14 precursor, CysC, albumin (ALB) precursor, and transferrin (TF), which could play roles in the apoptosis of the trabecular meshwork [87]. In a similar untargeted proteomic strategy, the aqueous humor was evaluated as a bodily fluid for biomarker discovery in PCG—an autosomal recessive disease caused by an abnormal development of the anterior chamber angle. The proteins apolipoprotein A4 (APOA4), ALB, and antithrombin 3 (SERPINC1) were found to be overexpressed in the aqueous humor of PCG patients, while TTR, PTGDS, opticin (OPT), and interphotoreceptor retinoid-binding protein (IRBP) were found at significantly lower levels, when compared to control subjects. These observations suggest that the retinoid pathways might be altered during the development of PCG, and highlight the differential regulation of TTR in this type of glaucoma contrary to previous observations in POAG [88].

Similarly, an untargeted pilot study conducted by Kaeslin et al. in 2016 [89], which involved the aqueous humor of five POAG patients and five age- and sex-matched controls undergoing cataract surgery, identified 34 upregulated and 53 downregulated proteins via shotgun proteomics LC–MS/MS. These significant differentially expressed proteins were involved in the cholesterol upregulation of angiotensinogen (AGT), apolipoprotein C-I (APOC1), and APOA4, inflammatory complement component C1q (C1q), complement component C8 beta chain (CO8B), complement C9 (CO9), and V-set and immunoglobulin domain-containing protein 4 (VSIG4), as well as metabolic-, antioxidant-, and proteolysis-related processes, indicating altered metabolic state, inflammatory response, and impaired antioxidant defense during glaucoma. Interestingly, from a total of 448 proteins identified, they found significant differences in 87 (accounting for ~20% of the proteins), showing high variability between both groups in a small cohort, which requires further validation by absolute quantitative proteomics in a larger population. In a similar untargeted approach, Kliuchnikova et al. [90] attempted in 2016 to define the aqueous humor proteome of PEXG, PES, and cataract patients via LC–MS/MS analysis, obtaining 36 proteins that were proposed to constitute the proteome of the fluid. Among them, the apolipoprotein D (APOD), quantified by label-free proteomics, was found to be decreased in PES patients, but no differences were observed in the POAG or PEXG groups. Later, the aqueous humor proteome of POAG and PACG, compared to patients with cataracts, was also explored via LC–MS/MS, obtaining 28 proteins as constitutive of the fluid proteome, of which the monocyte differentiation antigens CD14, CD59, complement factor D (CFD), APOA4, chromogranin A (CHGA), and MYB involved in immune response, the TIMP1 involved in the coagulation system, and the microfibril-associated glycoprotein 4 (MFAP4), agrin (AGRN), and apolipoprotein C-III (APOC3) involved in the response to light stimulus, were found to be upregulated [91].

In the same way, Adav et al. [92] carried out the proteomic characterization of the aqueous humor of POAG patients, observing significant alterations for 150 proteins (43 ± 18 upregulated and 105 ± 45 downregulated) when compared to control subjects with cataracts, most of which were related to the complement cascade, immune response, neural degeneration, and apoptosis. Moreover, the same group screened the aqueous humor proteome of PACG patients via LC–MS/MS and label-free quantitative proteomics, in a pilot study. Despite the limited number of samples (2 PACG and 3 controls), among the 1363 distinct proteins identified, more than 50% were differentially expressed in PACG (501 upregulated and 272 downregulated), which confirms the high intrinsic variability existing between individuals [93]. Recently, Sharma et al. [94] identified proteomics alterations in the aqueous humor of POAG patients (n = 15) and controls (n = 32) by means of LC–MS/MS, i.e., untargeted proteomics. The 33 overexpressed proteins were implicated in signaling, glycosylation, immune response, molecular transport, and lipid metabolism, highlighting Ig j chain C region (IGKC, 13.6-fold), inter-a-trypsin inhibitor heavy chain 4 (ITIH4, 4.1-fold), APOC3 (3.4-fold), and isocitrate dehydrogenase (NAD) subunit α (IDH3A, 3.1-fold) among the most upregulated ones, although no validation of these potential candidate biomarkers was carried out. A very interesting recent comprehensive review of the aqueous humor proteome of POAG patients again showed the great complexity of the proposed candidate markers, also depending on the type of surgery during aqueous humor sampling, i.e., glaucoma filtration surgery (GFS) or cataract surgery [95]. Hence, the untargeted studies carried out using LC–MS/MS in samples from POAG patients having undergone GFS identified up to 55 differentially expressed proteins (30 upregulated and 23 downregulated), while POAG patients having undergone cataract surgery provided up to 87 differentially altered proteins (30 upregulated and 57 downregulated). The roles of these proteins include activation of the acute immune response, dysregulation of folate metabolism, and alteration of the selenium micronutrient network. Interestingly, samples from POAG patients having undergone GFS showed enrichment in proteins of the complement system. Among the identified proteins significantly altered in glaucoma, eight were further studied using semi-targeted or targeted approaches, showing higher levels of ALB, APOC3, CysC, TIMP2, A2M, PGTDS, and ENPP2, and lower levels of SOD1, in POAG patients compared to control subjects. It should be noted that the aqueous humor proteome varies depending on POAG progression, and the complement system may affect glaucoma development.

Considering the small amount of samples available in the aqueous humor, targeted analyses using antibody microarrays against 1264 proteins were proposed to investigate the multiprotein expression levels in POAG patients and control subjects who underwent cataract extraction surgery. First, Izzotti et al. [96] identified 31 significantly altered proteins when comparing POAG (n = 10) patients and control (n = 14) subjects, of which 29 were upregulated (see Table 1) and related to oxidative and mitochondrial damage, neural degeneration, and apoptosis. Later, Saccà et al. [97] analyzed the aqueous humor obtained from 14 patients with POAG and 11 controls; it should be noted that 3 POAG cases refrained from pharmacological therapy for 72 h before sampling, showing no influence of therapy suspension in the aqueous humor proteome; hence, a total of 13 proteins were significantly increased (2.0–2.5-fold change) in glaucomatous patients compared with the expression levels of healthy controls, which reflects local damage to the anterior chamber, including the trabecular meshwork (see Table 1). However, both of these similarly designed studies provided very different results in terms of altered proteins as candidate biomarkers, showing very high heterogeneity and difficulty of normalization, although the same technology was used.

In a targeted quantitative proteomic study, several candidate biomarkers previously identified in patients diagnosed with Alzheimer’s disease were analyzed via multiplex immunoassays in the aqueous humor of POAG (n = 20) and PEXG (n = 32) patients and cataractous control subjects (n = 38). Specifically, upregulation of apolipoprotein A1 (APOA1), APOC3, apolipoprotein E (APOE), TTR, and α2-macroglobulin (α2M) was observed in both POAG (3.5-, 6.3-, 3.6-, 2.1-, and 7.0-fold, respectively) and PEXG (2.9-, 6.5-, 3.4-, 2.3-, and 7.5-fold, respectively) groups, compared with controls, which may reflect the severity of glaucoma [98]. The observed upregulation of α2M was previously reported after its quantification by WB in the aqueous humor of 12 patients with POAG, PEXG, and NVG and 9 controls, finding a 3.5-fold increase in glaucoma, probably exerting a neurotoxic effect by inhibiting the neuroprotective activity of nerve growth factor via TrkA receptors, and proposed as a potential biomarker or therapeutic target [99]. Finally, Doudevski et al., in 2014 [100], quantitated the levels of CLU, vitronectin (VTN), and the complement activation products C3a and soluble C5b-9 in the aqueous humor of PEXG patients (n = 67) and control subjects (n = 107), by means of ELISA. Significant upregulation was found for all of the studied proteins (see Table 1), evidencing local activation of the complement system and accumulation of sub-products with potent proinflammatory activity capable of triggering and chronically maintaining levels of subclinical inflammation, suggesting novel targets for therapeutic intervention.

#### 3.1.2. Metabolite-Based Biomarkers

Metabolomic profiling analysis of ocular fluids may contribute to a better understanding and earlier diagnosis of glaucoma, although the number of studies carried out to date is limited [101]. Most of the studies followed a targeted metabolomics approach, quantitating specific metabolic markers as candidates. For example, considering that the amino acid homocysteine (Hcy) may induce vascular injury and alterations to the extracellular matrix, Hcy levels were quantified in the aqueous humor of PEXG patients and control subjects by ELISA, observing two-fold elevation in the glaucoma group. This upregulation may contribute to the abnormal accumulation of the extracellular matrix, reflecting the impairment of the blood–aqueous barrier in glaucoma [102]. Another targeted analysis evaluated the presence and levels of diadenosine polyphosphate metabolites in the aqueous humor of POAG patients (n = 16) and controls (n = 10) by reverse phase LC–UV/Vis, observing increased concentration of the diadenosine tetraphosphate (Ap4A, 15-fold). The Ap4A may help to protect the autonomic innervation in the ciliary body/trabecular meshwork, reducing IOP by facilitating the drainage of the aqueous humor through the trabecular meshwork, and was therefore proposed as a possible glaucoma biomarker [103]. Later, the aqueous humor and blood levels of MDA and the blood adenosine triphosphate/adenosine diphosphate (ATP/ADP) were evaluated, in a targeted analysis by LC–photodiode-array detection (PDA), in 40 POAG patients and 26 age-matched controls. While MDA was observed to be upregulated in both the blood and aqueous humor of glaucoma patients, the ATP/ADP was found to be downregulated and, therefore, a decreased antioxidant defense capacity was expected [104].

Recent advances in metabolomics contributed to improve the understanding of the metabolic profile of the aqueous humor of glaucoma patients. A targeted quantitative approach by LC–MS/MS in 26 POAG subjects showed reduced concentrations of taurine and spermine and increased concentrations of creatinine, carnitine, three short-chain acylcarnitines, seven amino acids (glutamine, glycine, alanine, leucine, isoleucine, hydroxyl-proline, and acetyl-ornithine), seven phosphatidylcholines, one lysophosphatidylcholine, and one sphingomyelin in the glaucoma group when compared to controls (n = 26) [105]. Untargeted metabolomics by gas chromatography/time-of-flight mass spectrometry GC/TOF-MS in PCG [106] and POAG [107] patients revealed specific characteristics of each disease, with distinct metabolic profiles. In PCG patients, the identified amino acid glycine, urea, and phenylalanine were significantly different, proposing glycine as a potential biomarker for its earlier diagnosis [106]. For POAG patients, 14 candidate metabolic biomarkers were proposed, including the upregulated pelargonic acid and galactose 1, and the downregulated glucose-1 phosphate, sorbitol, and spermidine 2, with AUC from 0.62 to 0.86 [107]. Moreover, classical nuclear magnetic resonance (NMR) spectroscopy was used to determine the metabolic profiles of POAG (n = 30) and NTG (n = 30), showing similar profiles for both glaucomas when compared to controls. Interestingly, the glaucoma group showed a higher content for betaine, taurine, and glutamate, classifying groups with an AUC of 0.93, which may indicate increased oxidative stress, excitotoxicity, and the activation of neuroprotective mechanisms [108].

Overall, considering that the sampling of aqueous humor is highly invasive and limited to patients undergoing ocular surgery, any proposed biomarker will need an alternative non-invasive methodology for its determination. Furthermore, the potential of the hundreds of candidate biomarkers identified in the aqueous humor of glaucoma patients, as well as their relationships and the effects of medications on their levels, needs to be addressed, along with the urgent need to provide quantitative information beyond relative protein or metabolite concentrations.

**Table 1:** Candidate glaucoma biomarkers identified in aqueous humor (in addition to other fluids). The study, the strategy followed, the main analytical techniques used, the fold change of markers when specified, the number of subjects, and the disease of the patients analyzed are indicated.

Study	Fluid/Tissue	Strategy	Analytical Technique	List of Candidate Biomarkers (Fold Change vs. Controls) ^1^	Samples ^2^
Tripathi et al., 1994 [40]	Aqueous humor	Targeted proteomics	ELISA	Up ^3^: TGF-β2 (1.8-fold)	15 POAG, 10 CT
Tezel et al., 1997 [55]	Aqueous humor and plasma	Targeted proteomics	RIA	Up: ET (1.05-fold in aqueous humor)	31 POAG, 24 CT
Ferreira et al., 2004 [63]	Aqueous humor	Targeted quantitative analysis (activity assay)	Spectrophotometry	Up: SOD (1.7-fold), GPx (3.0-fold)	24 POAG, 24 CT
Määttä et al., 2005 [70]	Aqueous humor	Targeted proteomics	ELISA	Up: MMP-2 (2.1-fold PEXG vs. CT, 1.7-fold PEXG vs. POAG, 2.0-fold PES vs. CT), TIMP-2 (7.7-fold PEXG vs. CT, 3.0-fold POAG vs. CT, 6.0-fold PES vs. CT)	15 POAG, 16 PEXG, 15 PES, 10 CT
Min et al., 2006 [41]	Aqueous humor	Targeted proteomics	ELISA	Up: TGF-β2 (2.7-fold POAG vs. CT, 2.3-fold NVG vs. CT, 1.4-fold SOAG vs. CT)	43 glaucoma (14 POAG, 14 NVG, 15 SOAG), 20 CT
Yu et al., 2007 [42]	Aqueous humor	Targeted proteomics	ELISA	Up: TGF-β1 (control levels below detection limit), TGF-β2 (16-fold).	NVG, CT
Nolan et al., 2007 [43]	Aqueous humor	Targeted proteomics	ELISA	Up: sCD44 (2.2-fold)	90 POAG, 124 CT
Grus et al., 2008 [86]	Aqueous humor	Untargeted (discovery) and targeted proteomics (verification)	SELDI-TOF-MS, 2D electrophoresis, LC-MS/MS &ELISA	Up: TTR (1.9-fold)	52 POAG, 55 CT
Mokbel et al., 2010 [44]	Aqueous humor and plasma	Targeted proteomics	ELISA	Up: sCD44 (1.8-fold in aqueous humor), EPO (1,8-fold in aqueous humor)	39 POAG, 25 CT
Duan et al., 2010 [87]	Aqueous humor	Untargeted proteomics	2D electrophoresis and LC–MS/MS	Up: TTR (2.2-fold), CysC (5.2-fold), ALB (11.1-fold)	5 POAG, 5 CT
Ghanem et al., 2010 [66]	Aqueous humor	Targeted analysis	Spectrophotometric (enzymatic)	Up activity: GPx (2.9-fold), SOD (1.8-fold), MDA (8-fold)	30 POAG, 25 CT
Bai et al., 2011 [99]	Aqueous humor	Targeted proteomics	Quantitative WB	Up: α2M (3.5-fold)	12 glaucoma, 9 CT
Ghanem et al., 2011 [73]	Aqueous humor	Targeted proteomics	ELISA	Up: CTGF (3.1-fold PEXG vs. CT, 1.6-fold PEXG vs. POAG), TIMP-2 (4.8-fold PEXG vs. CT, 2.1-fold PEXG vs. POAG)	30 POAG, 30 PEXG, 25 CT
Browne et al., 2011 [75]	Aqueous humor	Targeted proteomics	ELISA	Up: CTGF (2.0-fold PEXG vs. CT, 1.9-fold PEXG vs. PES, 1.7-fold PEXG vs. POAG)	20 POAG, 18 PEXG, 15 PES, 21 CT
Takai et al., 2012 [46]	Aqueous humor	Targeted proteomics	Multiplex immunoassays	Up: IL-8 (2.3-fold POAG vs. CT, 4.0-fold PEXG vs. CT), TGF-β1 (5.0-fold POAG vs. CT, 12.5 PEXG vs. CT)	20 POAG, 23 PEXG, 21 CT
Bagnis et al., 2012 [67]	Aqueous humor	Targeted proteomics	Antibody microarray	Down ^4^: SOD (0.4-fold), GST (0.3-fold)	10 POAG, 10 CT
Saccà et al., 2012 [97]	Aqueous humor	Targeted proteomics	Antibody microarray	Up: APOE (2.1-fold)	14 POAG, 11 CT
Inoue et al., 2013 [98]	Aqueous humor	Targeted proteomics	Multiplex immunoassays	Up: APOC3 (6.3-fold POAG vs. CT, 6.5 PEXG, vs. CT), APOE (3.6-fold POAG vs. CT, 3.4-fold PEXG vs. CT), TTR (2.1-fold POAG vs. CT, 2.3-fold PEXG vs. CT), α2M (7.0-fold POAG vs. CT, 7.5-fold PEXG vs. CT)	20 POAG, 32 PEXG, 38 CT
Goyal et al., 2014 [68]	Aqueous humor	Targeted analysis	Spectrophotometric (enzymatic or biochemical)	Up activity: SOD (2.1-fold POAG vs. CT, 2.0-fold PACG vs. CT), GPx (2.5-fold POAG vs. CT, 2.3-fold PACG vs. CT)	30 POAG, 30 PACG, 30 CT
Doudevski et al., 2014 [100]	Aqueous humor	Targeted proteomics	ELISA	Up: CLU (1.8-fold)	68 PEXG, 107 CT
Ahoor et al., 2016 [56]	Aqueous humor and serum	Targeted analysis	ELISA	Up: ET-1 (1.2-fold PEXG vs. CT and 1.1-fold PES vs. CT in aqueous humor; 1.4-fold PEXG vs. CT and 1.4-fold PES vs. CT in serum)	15 PEXG, 15 PES, 15 CT
Ban et al., 2017 [53]	Aqueous humor	Targeted proteomics	ELISA	Up: Growth differentiation factor 15 (GDF15, 31.7-fold POAG vs. CT)	57 POAG, 23 CT
Wang et al., 2018 [77]	Aqueous humor	Targeted proteomics	Multiplex immunoassays	Up: OPN (1.2-fold)	41 PACG, 22 CT
Nikhalashree et al., 2019 [78]	Aqueous humor	Untargeted proteomics	LC–MS/MS	Up: OPN (unknown-fold, POAG vs. GT and PACG vs. CT), CysC (unknown-fold, POAG vs. CT, PACG vs. CT)	90 POAG, 72 PACG, 78 CT
Guo et al., 2019 [49]	Aqueous humor	Targeted proteomics	ELISA	Up: TGF-β2 (1.3-fold in POAG vs. CT)	25 POAG, 21 CACG, 9 PACS, 45 AACG, 26 CT
Can Demirdöğen et al., 2019 [76]	Aqueous humor and tears	Targeted proteomics	ELISA	Up: CTGF (1.6-fold PEXG vs. CT, 1.5-fold PES vs. CT, in tear)	Tear: 78 PEXG, 77 PES, 78 CT. Aqueous Humor: 8 PEXG, 17 PES, 23 CTs
ten Berge et al., 2019 [51]	Aqueous humor	Targeted proteomics	Multiplex immunoassays	Up: IL-8 (1.5-fold POAG vs. CT, 1.5-fold AMD vs. CT)	28 glaucoma(22 POAG, 1 NTG, 4 NAG, 1 SGPDS), 12 AMD, 25 RP, 22 CT
Can Demirdöğen, et al., 2020 [80]	Aqueous humor and tears	Targeted proteomics	ELISA	Up: CLU (2.0-fold PEXG vs. CT, 2.4 PEXG vs. PES, in aqueous humor)	12 PEXG, 22 OES, 22 CT
Sun et al., 2020 [52]	Aqueous humor	Targeted Proteomics	ELISA	Up: VEGF-A (1.4-fold Stable NVG vs. CT, 1.2-fold Stable-NVG vs. CRVO, 1.1-fold Stable-NVG vs. NPDR, 1.2-fold Stable-NVG vs. BRVO), IL-8 (1.4-fold Stable-NVG vs. CT, 1.1-fold Stable-NVG vs. CRVO), EPO (1.3-fold Stable-NVG vs. CT, 1.2-fold Stable-NVG vs. BRVO)	12 NVG, 26 Stable-NVG, 11 CRVO, 18 PACG, 25 PDR, 7 BRVO, 22 CT
Sun et al., 2020 [48]	Aqueous humor and vitreous body	Targeted Proteomics	ELISA	Up: VEGF-A (1.2-fold NVG vs. PDR in aqueous humor)	15 NVG, 17 PDR
Hubens et al., 2020 [95]	Aqueous humor	Targeted proteomics	LC–MS/MS	Up: ALB, APOC3, CysC, TIMP2, A2M, PGTDS, ENPP2	POAG vs. CT
				Down: SOD1	
Lin et al., 2020 [54]	Aqueous humor	Targeted proteomics	ELISA	Up: GDF15 (unknown-fold, POAG vs. CT, PEXG vs. CT)	6 POAG, 6 PEXG
Burgos-Blasco et al., 2020 [60]	Aqueous humor and tears	Targeted proteomics	Multiplex immunoassays	Up in aqueous humor: IFN-γ (1.7-fold), VEGF (2.3-fold).	27 POAG, 29 CT
Igarashi et al., 2021 [50]	Aqueous humor	Targeted proteomics	Immunoenzymatic assay and multiplex immunoassay	Up in aqueous humor: TGF-β1 (SOAG vs. CT, PEXG vs. CT, PEXG vs. SOAG, PEXG vs. POAG), TGF-β2 (POAG vs. CT, SOAG vs. CT, POAG vs. PEXG, SOAG vs. PEXG)	97 POAG, 48 SOAG, 48 PEXG, 88 CT
				Down in tear: TGF-β2 (PEXG vs. CT)	
Bleich et al., 2004 [102]	Aqueous humor and plasma	Targeted metabolomics	ELISA	Up: Hcy (2.0-fold in aqueous humor, 1.3-fold in plasma)	29 PEXG, 31 CT
Castany et al., 2011 [103]	Aqueous humor	Targeted metabolomics	HPLC ^6^–UV/Vis	Up: Ap4A (15-fold)	16 POAG, 16 CT
Chen, et al., 2019 [106]	Aqueous humor	Untargeted metabolomics	GC/TOF-MS	Up: Glycine-2 (8.9-fold PCG vs. CT, 3.9-fold PCG vs. POAG, 9.0-fold PCG vs. ARC), Phenylalanine-1 (1.8-fold PCG vs. CT, 1.5-fold PCG vs. ARC)	45 PCG, 10 CCs, 10 ARCs, 10 POAG
				Down: Phenylalanine-1 (0.9-fold PCG vs. POAG), Urea (0.9-fold PCG vs. POAG, 0.6-fold PCG vs. CT, 0.8-fold PCG vs. ARC)	

^1^ Comparison with other groups is indicated in brackets. ^2^ CT: control; PESL: pseudoexfoliation syndrome plus luxation (complications); PDR: proliferative diabetic retinopathy; CRVO: central retinal vein occlusion; NPDR: non-proliferative diabetic retinopathy; BRVO: branch retinal vein occlusion; CCs: congenital cataracts; ARCs: age-related cataracts. ^3^ Up: upregulated; ^4^ Down: downregulated.

### 3.2. Eye Tissues and Vitreous Body

Although more invasive in terms of sampling, the study of the trabecular meshwork offers the advantage of reflecting specific changes in the aqueous humor outflow dysfunction. Moreover, the proximity of the vitreous body and surrounding tissues to the RGCs makes them very attractive targets for biomarker discovery. However, sampling procedure requires the entry of a body cavity, the interruption of normal body functions, and tissue removal—i.e., biopsy—and therefore the candidate biomarkers are certainly very difficult to implement in clinical practice; Table 2 shows the most relevant studies with consistent candidate markers, while an extended bibliography is covered in Table S2 of the Supplementary Materials.

#### 3.2.1. Vitreous Body

• Protein-based biomarkers

The vitreous body is a hydrogel-like substance located between the lens and the retina, accounting for about 80% of the volume of the eyeball, and mainly consisting of water (98%), collagens, glycosaminoglycans, and amino acids. The proximity of the vitreous body to the RGCs fostered works aiming at local biomarker discovery, which may be a more attractive target than the tear film or aqueous fluids, but limited in terms of sampling accessibility. In this vein, in a targeted approach, a multiplexed immunoassay was used to characterize the angiogenic and inflammatory vitreous profiles in NVG and a set of ischemic retinopathies. Hence, numerous proteins were significantly elevated, with significant differences for PGF, VEGF-A, IL-6, and IL-8 [109]. Alterations of N-glycans were also identified in the vitreous fluid of patients with NVG secondary to PDR, observing upregulation of total and sialylated N-glycans [110]. Continuing with the suggested proinflammatory environment observed during glaucoma, the analyzed vitreous levels of the cytokines IL-2, IL-5, MCP-1, TNF-α, and IFN-γ-induced protein-10 (IP-10) were significantly higher in AACG, while IP-10 was also upregulated in both POAG and CAGG, when compared to controls, confirming the local inflammation and immune reaction during glaucoma [111]. Furthermore, a very interesting and comprehensive study of the vitreous fluid and retinas of POAG patients using multiplexed tandem mass tag-based proteomics (TMT-MS3) yielded 252 and 554 upregulated and 133 and 559 downregulated proteins in the retina and vitreous body, respectively, of which 122 were found to be linked to Alzheimer’s disease [112].

• Metabolite-based biomarkers

Metabolomics studies in tissues or biofluids from eye donors are very scarce at present. The potential role of glutamate in the excitotoxicity during glaucoma and consequent apoptosis fostered the determination of this metabolite—along with others including glycine, aminobutyric acid, alanine, etc.—in the vitreous body of glaucoma patients. Higher levels of glutamate (2.0.fold) and glutamate/glutamine–creatinine ratio were observed in this fluid from glaucoma patients compared to healthy controls, using single-voxel NMR spectroscopy or HPLC analysis, respectively [113,114]. However, these results were not replicated by Honkanen et al., who observed no changes in the levels of glutamate in vitreous samples from glaucoma patients, although the smaller cohort and their higher heterogeneity could have had an effect [115], and additional studies are therefore needed in order to fully elucidate the role of glutamate in the pathogenesis of glaucoma, and its usefulness as a candidate biomarker.

#### 3.2.2. Retina and Optic Nerve

• Protein-based biomarkers

The study of eye tissues and/or cells may provide valuable information regarding the identification of molecules altered in order to be further determined at the systemic level. For example, histopathological studies revealed an increased immunostaining for TNF-α and TNF-α receptor in the glaucomatous optic nerve head and retina sections compared to age-matched control eyes, suggesting that TNF-α-mediated cell death is involved in the neurodegeneration process of glaucoma, in agreement with studies performed in the aqueous humor and vitreous body [116,117,118]. On the other hand, the hypoxia-inducible factor 1α (HIF-1α) was identified as being upregulated in the optic nerve head and retina of glaucomatous donors compared to control subjects, showing local tissue hypoxia in relation to glaucoma [119]. Moreover, the enzyme peptidyl arginine deiminase 2 (PAD2), converting arginine to citrulline, was found only in POAG optic nerves after protein identification by LC–MS/MS, although no data of this enzyme at the systemic level have been published to date [120]. On the other hand, a preliminary study using immunohistochemistry determined lower expression of aquaporin (AQP)-9 in the optic nerve of a POAG patient compared to a control subject [121].

Likewise, the quantitative analysis by LC–MS/MS of human retinal protein samples from glaucoma (n = 10) and control (n = 10) donors, showed many upregulated retinal proteins in glaucoma involved in TNF-α/ tumor necrosis factor receptor 1 (TNFR1) signaling, which is implicated in death receptor-mediated caspase cascade, mitochondrial dysfunction, endoplasmic reticulum stress, calpains leading to apoptotic cell death, the nuclear factor-κB (NF-κB) and JAK/STAT pathways, and inflammasome-assembly-mediating inflammation [122]. Moreover, higher levels of hemoglobin (Hb) were observed in glaucomatous retinas with respect to control donors, appearing to be an intrinsic protective mechanism to facilitate cellular oxygenation and free radical scavenging [123]. In addition, the complement system was also observed via proteomic analysis to be dysregulated in glaucoma patients, detecting the expression and differential regulation of several complement components, including proteins involved in the classical and the lectin pathways of complement activation [124]. Funke et al., in 2016 [125], identified more than 600 proteins in retina samples, of which the altered candidates were involved in cellular development, stress, and cell death. Three of them were downregulated in glaucomatous retinas when compared to controls—the ADP/ATP translocase 3 (ANT3, 0.4-fold), the PC4 and SRFS1-interacting protein 1 (DFS70, 0.8-fold), and the methyl-CpG-binding protein 2 (MeCp2, 0.6-fold)—but the clinical phenotypes of glaucoma donors were not defined, this being a distinct limitation of the study.

#### 3.2.3. Trabecular Meshwork

• Protein-based biomarkers

Prior untargeted proteomic analysis in trabecular meshwork cells from POAG patients revealed the upregulation of the cochlin [126] and copine-1 [127] proteins, after comparison with age-matched control donors, suggesting that cochlin may disrupt the trabecular meshwork architecture and contribute to degradation of the extracellular matrix, resulting in cell aggregation, while copine-1 may play a role in the abnormal intracellular calcium signaling pathway in the glaucomatous trabecular meshwork. On the other hand, elevated levels of CAPN10 were found in the glaucomatous trabecular meshwork, but its activity was 0.5-fold lower in POAG patients than in controls [128]. Considering that during glaucoma extensive remodeling of the trabecular meshwork is observed, Micera et al. [129] aimed in 2016 to characterize proteins related to tissue remodeling, inflammation, and growth factor pathways in glaucomatous trabecular meshwork tissues using protein array analysis. Interestingly, they observed upregulation of IL-10, IL-6, IL-5, IL-7, IL-12, IL-3, MIP1δ/α, VEGF, TGF-β1, and soluble TNFR1, and downregulation of IL-16, IL-18, intercellular adhesion molecule 3 (ICAM3), MMP7, and TIMP1.

**Table 2:** Candidate glaucoma biomarkers identified in the retina, optic nerve, vitreous body, or trabecular meshwork. The study, the strategy followed, the main analytical techniques used, the fold change of markers when specified, the number of subjects, and the disease of the recruited patients are indicated.

Study	Fluid/Tissue	Strategy	Analytical Technique	List of Candidate Biomarkers (Fold Change vs. Controls) ^1^	Samples
Tezel, et al., 2001 [118]	Retina	Targeted proteomics	Immunohistochemistry	Up ^2^: TNF-α, TNFR1 (Not-applicable fold)	14 POAG (20 eyes), 10 CT (20 eyes)
Govindarajan et al., 2008 [128]	Trabecular meshwork	Targeted analysis	WB and spectrophotometric	Up: CAPN10 (unknown fold)	15 POAG, 15 CT
				Down ^3^: CAPN10-activity (0.5-fold)	
Tezel et al., 2010 [124]	Retina	Targeted proteomics	LC–MS/MS	Down: Complement factor H (CFH	10 glaucoma, 10 CT
Yang et al., 2011 [122]	Retina	Targeted proteomics	LC–MS/MS (label free) and WB	Up: TNF-α (3.1-fold), CAPN10 (2.0-fold).	10 glaucoma, 10 CT
Kovacs et al., 2015 [109]	Vitreous body	Targeted proteomics	Multiplex immunoassays	Up: VEGF-A (79.5-fold NVG vs. non-DM), IL-6 (164.9-fold NVG vs. non-DM), IL-8 (30.1-fold NVG vs. non-DM).	12 NVG, 29 PDR, 10 DM ^4^, 29 non-DM
Micera et al., 2016 [129]	Trabecular meshwork	Targeted proteomics	Multiplex immunoassays	Upregulated: IL-10 (23.8-fold), IL-6 (14.6-fold), IL-5 (13.3-fold), IL-7 (12.5-fold), IL-12p70 (8.7-fold), IL-12p40 (7.7-fold), IL-3 (4.4-fold), IL-21 (3.7-fold), IL-4 (3.7-fold), IL-33 (3.2-fold), TNFα (4.5-fold), IFN-γ (2.3-fold), IL-15 (2.2.fold), IL-2 (2.1-fold), IL-1β (1.7-fold), IL-17 (1.6-fold), IL-8 (1.4-fold), IL-34 (1.3-fold), VEGF (6.1-fold), TGF-β1 (6.1-fold), FGF-β (3.9-fold), nerve growth factor β (NGF-β, 3.8-fold), BDN (3.1-fold), MMP1 (2.0-fold), MMP2 (3.2-fold), TIMP2 (1.8-fold)	40 POAG, 23 CT
				Down: IL-18 (0.08-fold), IL-16 (0.02-fold), MMP7 (0.5-fold), TIMP4 (0.4-fold)	
Tong et al., 2017 [111]	Vitreous body	Targeted proteomics	Multiplex immunoassays (cytometric)	Up: IL-2 (3.4-fold AACG vs. CT), IL-5 (1.34 AACG vs. CT), MCP-1 (5.4-fold AACG vs. CT, 1.4-fold POAG vs. CT), TNF-α (1.8-fold AACG vs. CT), IP-10 (7.0-fold AACG vs. CT, 2.4-fold CAGG vs. CT, 2.8-fold POAG vs. CT)	29 glaucoma (8 AACG, 15 CACG, 6 POAG), 28 CT
Dreyer et al., 1996 [113]	Vitreous body	Targeted metabolomics	HPLC	Upregulated: Glutamate (2.0-fold)	26 Glaucoma, 21 CT
Doganay et al., 2012 [114]	Vitreous body	Targeted metabolomics	Magnetic resonance spectroscopy (MRS)	Up: Glutamate/glutamine–creatine ratio (Glx/Cr, 4.8-fold)	29 POAG, 13 CT

^1^ Comparison with other groups is indicated in brackets. ^2^ Up: upregulated; ^3^ Down: downregulated; ^4^ DM: diabetes mellitus.

### 3.3. Tear Film

The tear film, covering the ocular surface, is a very interesting thin fluid layer of the eye tissue for the discovery and implementation of glaucoma biomarkers, since it contains a comparatively simple proteome composed of a variety of molecules, some of which have historically been proposed as glaucoma- or drug-induced inflammatory molecules [130]. More importantly, this fluid permits a noninvasive procedure, not requiring incision into the body or tissue removal, and sampling by Schirmer’s test papers or glass microcapillaries is affordable. The tear film is a trilaminar and dynamic fluid covering the entire ocular surface, consisting of mucus, aqueous, and lipid layers with deep interaction between them. Tears nourish the ocular surface and remove local waste products, drugs, and disease-related media. Importantly, the medical therapy used in glaucoma profoundly disturbs the homeostasis of the ocular surface by altering components of the tear film. However, many studies targeted the tear film to explore the discovery of potential biomarkers of glaucoma [131] (see Table S3 of the Supplementary Materials); Table 3 summarizes those candidates already observed in more than one study.

#### 3.3.1. Protein-Based Biomarkers

Studies exploring the tear proteome and its possible alterations in relation to glaucoma showed inflammatory response as a common altered pathway. Pieragostino et al. [132] focused on characterizing protein patterns in the tears of patients with medically controlled POAG and PEXG. In this comparative proteomic analysis, carried out via label-free LC–MS/MS, altered expression of proteins related to inflammation pathways was observed, including the further validated lysozyme C (LYZ), LCN1, protein S100, immunoglobulins, prolactin-inducible protein, and phosphorylated cystatin-S (CST4). A key question arises as to whether the proposed biomarkers may be related to disease or induced by topical therapy. Several studies analyzed the tear proteome profiles of patients receiving glaucoma medications, in order to identify specific regulated pathways induced by eye drops. Thus, there seems to be common consensus on the increase in proinflammatory cytokine overexpression in response to the topical treatments for glaucoma. For example, the cytokines IL-1β, IL-6, IL-12, TNF-α, and several MMPs were increased while TIMPs were decreased in treated patients, while chronic treatments induced expression of the S100-A8, S100-A9, mammaglobin B, and 14–3-3 z/d proteins [133,134,135]. In this vein, the levels of cytokines, quantified by multiplex immunoassays, were significantly higher in the tears of POAG and PACG patients compared to their concentrations in aqueous humor, while tear concentrations of IFN-γ, granulocyte-macrophage colony-stimulating factor (GM-CSF), and IL-5 were significantly lower in patients who developed complications from glaucoma after one year. However, the authors suggested that the administration of different drugs did not modulate the levels of the analyzed cytokines [136]. A recent study evaluated the influence of topical preservatives used in antiglaucoma drugs, by comparing topical benzalkonium-chloride-preserved timolol or topical benzalkonium-chloride-preserved brimonidine with topical preservative-free timolol or control subjects without any treatment. The results showed that the use of benzalkonium-chloride-preserved topical medications induced oxidative stress in the tear film, with increased SOD, CAT, and GPx activities as well as higher levels of advanced oxidation protein products (AOPPs), total oxidant status (TOS), and oxidative stress index [137]. Furthermore, the use of topical drugs with preservatives had an impact on the tear cytokine expression levels, with significantly higher levels of IL-2, IL-5, IL-10, IL-12 (p70), IL-13, IL-15, IL-17, FGF-β, platelet-derived growth factor-BB (PDGF-BB), and TNF-α in patients receiving treatments with preservatives, compared to controls [138]. Therefore, the topical use of glaucoma medications resulted in specific changes of inflammatory or oxidative stress markers in the tear film.

In a targeted approach, the levels of the brain-derived neurotrophic factor (BDNF)—a vital component for the survival and differentiation of neurons—were determined by ELISA in the tears of NTG patients (n = 20) and control subjects (n = 20), observing a significant upregulation in glaucomas (3.2-fold), with potential use as a biochemical marker for early detection of NTG, in combination with other markers [139]. The differential expression of MMP2 and MMP9 was also evaluated in non-treated POAG, PACG, PEXG, PES, and control individuals via gelatin zymography. Increased tear MMP-9 activity was observed in all glaucoma groups at early stages of the disease, and in PES subjects was higher than in control and advanced glaucoma, suggesting activation of the extracellular matrix in the early stages of the disease [140]. However, a similar trend has been observed in POAG and NTG patients treated with prostaglandin analogs [141], where MMP-9 expression was higher in POAG subjects while MMP-2 expression was higher in NTG patients. Moreover, according to Reddy et al. [142], patients diagnosed with POAG showed marginally elevated tear levels of cytokines involved in tissue remodeling, while NTG showed elevated levels of cytokines regulating allergic pathways. Finally, the ciliary neurotrophic factor (CNTF), participating in oxygen consumption regulation, has been proposed as a glaucoma biomarker, since lower levels of CNTF were found in the tear film and aqueous humor of patients with POAG (n = 55) compared to control subjects (n = 61) [143].

Considering the existing influence of topical drugs, tears from patients with POAG naïve to therapy were analyzed following a shotgun proteomics approach by Pieragostino et al. [144]. The authors identified 25 upregulated and 2 downregulated proteins in non-medicated POAG patients (see Table 3 and Table S3), which also involved biochemical networks linked to inflammation. Interestingly, a subgroup of 12 upregulated proteins in naïve POAG patients was found to be downregulated in the medically controlled POAG groups treated with prostaglandin analogs, i.e., LCN1, LYZ, lactotransferrin (LTF), proline-rich protein 4 (PRR4), prolactin-inducible protein (PIP), AZGP1, polymeric immunoglobulin receptor (PIGR), CST4, IGKC, Ig alpha-2 chain C region (IGHA2), immunoglobulin J chain (IGJ), and Ig alpha-1 chain C region (IGHA1). Recently, a multiplexed analysis of proinflammatory cytokines in the tear film of eye-drop-naïve patients with newly diagnosed POAG and control subjects showed that mean concentrations of most cytokines were lower in the glaucoma group, with significant differences for IL-12p70 [145]. Still, contradictory observations regarding the inflammatory environment in the glaucomatous tear film are present, and therefore this biofluid merits additional research in terms of the origin of the inflammatory environment [146].

#### 3.3.2. Metabolite-Based Biomarkers

Limited studies have been published in the field of metabolomics in tears from glaucomatous patients. The Hcy concentrations were quantified by HPLC and fluorescence detection in the tear fluid and plasma of PEXG (n = 30) patients and healthy control (n = 30) subjects, observing significantly higher levels of this amino acid in both biofluids in patients with secondary glaucoma, and further studies are therefore warranted to investigate whether increased Hcy levels in tear fluid might be implicated in the failure of filtering blebs in PEXG eyes [147]. A very interesting work explored an integrative analysis of tears from POAG patients naïve to therapy and healthy subjects, using a targeted metabolomics and untargeted proteomics strategy [148]. The main results showed that POAG patients had lower levels of several tear amino acids and lysophospholipids compared with controls—i.e., alanine, arginine, glycine\lysine, leucine\isoleucine\proline-OH, methionine, phenylalanine, proline, valine, C2, C22:0-lisofosfatidilcolina(LPC), and C24:0-LPC—lower concentrations of thioredoxin (TXN), actin, and ACTG1, and higher levels of the proteins LYZ, junction plakoglobin (JUP), and protein PML. Overall, the low amount of acetylcarnitine in POAG patients seemed to correlate with the proteomics data.

**Table 3:** Candidate glaucoma biomarkers identified in tear film. The study, the strategy followed, the main analytical techniques used, the fold change of markers when specified, the number of subjects, and the disease of the recruited patients are indicated.

Study	Fluid/Tissue	Strategy	Analytical Technique	List of Candidate Biomarkers (Fold Change vs. Controls) ^1^	Samples
Ghaffariyeh et al., 2009 [139]	Tears	Targeted proteomics	ELISA	Up ^2^: BDNF (3.2-fold)	20 NTG, 20 CT
Pieragostino et al., 2012 [132]	Tears	Untargeted proteomics	LC–MS/MS (label free) and SDS-PAGE+MALDI-MS ^3^	Altered: LYZ, LCN1, immunoglobulins, PIP, CST4	Discovery: 4 POAG, 5 PEXG, 4 CTs. Validation: 9 POAG, 7 PEXG, 8 CT
Pieragostino et al., 2013 [144]	Tears	Shotgun proteomics	LC–MS/MS	Up: ALB (1.7-fold), CST4 (1.7-fold), ACTG1 (1.9-fold), TF (2.1-fold), PIP (2.4-fold), LTF (2.6-fold), LYZ (2.7-fold), proline-rich protein 1 (PROL1, 2.9-fold), LCN1 (2.9-fold)	9 POAG, 10 CT
				Down ^4^: IGHG3 (Unknown-fold)	
Gupta et al., 2017 [145]	Tears	Targeted proteomics	Multiplexed ELISA	Down: IL-12P70 (0.6-fold)	10 POAG, 9 CT
Sahay et al., 2017 [140]	Tears	Targeted proteomics	Gelatin zymography	Up: MMP-9 (2.5-fold POAG vs. CT, 2.2-fold PACG vs. CT, 2.1-fold PES vs. CT), MMP-2 (1.1-fold POAG vs. CT, 1.1-fold PES vs. CT)	27 POAG, 27 PACG, 22 PEXG, 40 PES, 35 CTs
				Down: MMP-2 (0.7-fold PACG vs. CT)	
Shpak et al., 2017 [143]	Tears, aqueous humor, and serum	Targeted proteomics	ELISA	Down: CNTF (0.7-fold in Aqueous Humor of POAG vs. Cataract, 0.6-fold in Tear of POAG vs. Cataract)	55 POAG, 61 Cataracts, 29 CT
Martinez-de-la-Casa et al., 2017 [138]	Tears	Targeted proteomics	Multiplexed immunoassay	Up: IL-2, IL-5, IL-10, IL-12 p70, IL-13, IL-15, IL-17, FGF basic, PDGF-BB, TNF-α in POAG (preservative vs. CTs)	20 POAG (preservative), 20 POAG (preservative-free), 39 CT
Reddy et al., 2018 [142]	Tears	Targeted Proteomics	Gelatin zymography, ELISA, and multiplex immunoassay	Up: MMP-9 (7.1-fold POAG vs. CT, 5.7-fold NTG vs. CT, 1.2-fold POAG vs. NTG), MMP-2 (2.6-fold POAG vs. CT, 3.3-fold NTG vs. CT, 0.8-fold POAG vs. NTG), TIMP-1 (1.3-fold POAG vs. CT, 1.2-fold POAG vs. NTG), IP-10 (1.8-fold POAG vs. NTG), macrophage derived chemokine (MDC, 1.9-fold POAG vs. NTG), platelet derived growth factor-AA (PDGF-AA, 3.8-fold POAG vs. NTG), IL-1α (1.2-fold POAG vs. NTG), IL-8 (1.6-fold POAG vs. NTG), IL-7 (1.3-fold NTG vs. POAG), MCP-1 (1.3-fold NTG vs. POAG), TNF-β (1.3-fold NTG vs. POAG)	30 POAG, 30 NTG, 30 CT
				Down: MMP-1 (0.8-fold POAG vs. CT, 0.8-fold POAG vs. NTG)	
Csősz et al., 2019 [136]	Tears and aqueous humor	Targeted proteomics	Multiplexed immunoassay	Down: IFN-γ, IL-5 in tears of patients who developed complications after one year	12 POAG, 8 PACG
Sedlak et al., 2020 [137]	Tears	Targeted analysis	Spectrophotometric (enzymatic and non-enzymatic)	Up: SOD (unknown-fold), CAT (unknown-fold), GPx (unknown-fold), AOPP (1.1 BR+BAC vs. CT or T, 1.1-fold T+BAV vs. CT or T), Total Oxidant Status (TOS, 1.2-fold BR+BAC vs. CT or T), 1.2-fold T+BAC vs. CT or T), Oxidative Stress Index (OSI, 1.1-fold BR+BAC vs. CT or T, 1.21 T+BAC vs. CT or T).	17 glaucoma-preservative-free, 24 glaucoma-BAC-preserved 0.5% timolol (T+BAC), 19 glaucoma-BAC-preserved brimonidine (BR+BAC), 25 CT
Roedl et al., 2007 [147]	Tears and plasma	Targeted metabolomics	HPLC-fluorescence	Up: Hcy (1.8-fold in tear fluid, 1.4-fold in plasma)	30 PEXG, 30 CT
Rossi et al., 2019 [148]	Tears	Targeted metabolomics and untargeted proteomics	Direct infusion UPLC–MS/MS (DIMS, metabolomics) and LC–MS/MS (label-free proteomics)	Up-proteins: LYZ	16 POAG, 17 CT
				Down-proteins: ACTG1	
				Down-metabolites: Alanine (0.7-fold), arginine (0.6-fold), glycine\lysine (0.7-fold), leucine\isoleucine\proline-OH (0.6-fold), methionine (0.7-fold), phenylalanine (0.6-fold), proline (0.7-fold), valine (0.7-fold), C2 (0.5-fold), C22:0-LPC (0.5-fold), C24:0-LPC (0.5-fold)	

^1^ Comparison with other groups is indicated in brackets. ^2^ Up: upregulated; ^3^ MALDI–MS, matrix-assisted laser desorption/ionization tandem mass spectrometry; ^4^ Down: downregulated.

### 3.4. Serum/Blood

Since the blood can be sampled easily, through a minimally invasive procedure, identification of altered molecules that can be measured in this fluid appears preferential, but may represent a diffuse way to identify candidate biomarkers of glaucoma. Blood, plasma, and serum are great reservoirs of signaling molecules, proteins, and metabolites secreted from different types of cells. Consequently, the proteins and metabolites detectable in serum or plasma have formed the basis of commonly used tests to screen and monitor disease biomarkers in various fields, being very attractive for the early diagnosis of glaucoma disease. However, similar to the above-discussed ocular fluids and eye tissues, plenty of candidate glaucoma biomarkers have been proposed, while their validation is limited today (see Table S4 of the Supplementary Materials). Accordingly, in the following subsections and in Table 4 we have tried to summarize the most noteworthy investigations that include common candidates.

#### 3.4.1. Protein-Based Biomarkers

The correlation of glaucoma biomarkers in circulating fluid may be made specific to the eye, although other conditions, including neurodegenerative disorders, can overlap possibly existing systemic changes. Altered pathways involved in glaucoma identified to date include stress, apoptosis, DNA repair, cell adhesion, tissue remodeling, transcription regulation, vascular factors, immune- and inflammatory-related factors, etc., systemic analysis of which may provide potential utility in the diagnosis and prognosis of glaucoma [21].

• Oxidative-stress-related markers

The oxidative injury and the altered antioxidant defense mechanisms observed in the pathophysiology of glaucomatous degeneration fostered the evaluation of systemic oxidative stress parameters and/or related antioxidants. One of the first works studying the systemic level of antioxidant enzymes determined the activities of myeloperoxidase, CAT, and plasma MDA in POAG (n = 40) and control (n = 60) subjects. While no significant differences were observed for CAT and MPO activities, the plasma MDA levels were significantly higher in glaucomatous patients than in the control subjects (2.3-fold) [149]. In the same year, patients with POAG exhibited low levels of circulating reduced glutathione (GSH) (0.7-fold), suggesting a systemic compromise of the antioxidant defense [150]. Later, serum oxidative-stress-related molecules were shown to be altered in glaucoma patients. In a targeted analysis, the determination of serum oxidative degradation products in 160 glaucoma patients with different etiology and 31 controls demonstrated that total antioxidant capacity (TAC), SOD, and GPx were all found to be decreased, while MDA, serine, TF, and vitamins A and E were increased in glaucoma patients, revealing systemic lipid oxidation on the basis of glaucoma, and vitamin E as a potential neuroprotective agent [151]. A similar trend was observed during the quantification of CAT, SOD, and GPx activity in the red blood cells of POAG (n = 20) subjects, with significant downregulation when compared to controls [152]. On the other hand, Zanon-Moreno et al. [153] studied the association of selected polymorphism in genes related to vitamin C and GPx in a large cohort of POAG (n = 250) and control subjects (n = 250), observing that, in POAG patients, the levels of vitamin E and C were lower, while the GPx activity was higher, when compared to control subjects. However, a later study showed a similar trend to previous studies carried out in the aqueous humor [68], once again indicating conflicting results, with high variability depending on the population.

In addition to broadly determined oxidative markers, lipofuscin fluorescence was analyzed in POAG and control individuals, both with cataracts. A significant increase in lipofuscin, MDA, and TOS were observed in POAGs, while total and mitochondrial SOD activity were lower, when compared to control subjects [154]. Moreover, in PEXG (n = 58) and PES (n = 47) patients the MDA and GSH levels were significantly higher than in controls (n = 134), while the activity of SOD and CAT were lower in both glaucoma and PES groups, with specific downregulation of nitric oxide concentration in PEXG, compared to PES and controls [155]. Tanito et al., in 2012 [156] evaluated the systemic levels of pro-oxidants and antioxidants in Japanese POAG (0 = 206), PEXG (n = 199) and control (n = 126) individuals via spectrophotometry, determining lower ferric-reducing activity in POAG patients when compared to control subjects, and lower thiol antioxidant activity in PEXG subjects with respect to POAG and control groups. In a prospective study carried out on POAG (n = 30), OHT (n = 30), and control (n = 30) individuals, the native thiol and total thiol levels were significantly lower in POAG and OHT patients than in control subjects, while the ischemia-modified ALB presented higher levels in both glaucoma groups when compared to controls [157]. In the same way, the levels of ischemia-modified ALB were higher in Turkish POAG patients compared to controls, while total thiol and native thiol levels were significantly lower in the glaucoma group [158], in contrast to the higher disulfide and the ratio of disulfide vs. total of native thiols observed.

Likewise, evaluating oxidative stress may help in understanding the course of PACG, and oxidative stress damage might be a relevant target for both prevention and therapy of this glaucoma subtype. Upregulation of oxidative stress parameters was observed in the serum of PACG patients (n = 50), although with a slightly increased fold change for MDA, conjugated diene, 4-hydroxynonenal, AOPP, 8-hydroxydeoxyguanosine (8-OHdG), and ischemia-modified ALB, further limiting classification of the disease [159]. However, in a recent prospective study, the serum levels of SOD and total antioxidant status (TAS) determined spectrophotometrically in PACG patients were significantly lower than those in the control group, while MDA and hydrogen peroxide were higher in individuals with glaucoma, with 5.5- and 2.2-fold change, respectively, suggesting that oxidative stress may be involved in the onset and development of PACG [160]. Moreover, the analysis of thiol/disulfide homeostasis may indirectly reflect the increase in oxidative stress and DNA damage, along with the ischemia-modified ALB.

Systemic antioxidant status has been spectrophotometrically studied by Abu-Amero et al. [161], showing lower plasma levels of TAS in POAG patients compared to control subjects, supporting once again the role of oxidative-stress-based mechanisms in the pathogenesis of POAG. The simultaneous study of aqueous humor and serum also revealed antioxidant alterations and DNA damage in glaucoma patients, according to Sorkhabi et al. [162]. Thus, while the TAS in bodily fluids was lower, the levels of the marker of oxidative DNA damage—8-OHdG—were significantly higher in both POAG and PEXG than in control subjects, indicating that the excessive production of oxidative species in the cells induces oxidative damage in the DNA. Similarly, 8-OHdG was recently determined via ELISA in the plasma of POAG (n = 50) and control subjects (n = 45), with significantly elevated levels in POAG cases, and specifically in male subjects, when compared to controls, with an AUC of 0.653, 78% sensitivity, and 53% specificity. This further evidences the role of systemic oxidative-stress-induced DNA damage in the pathogenesis of POAG, although plasma 8-OHdG seems poor as a potential biomarker [163]. Finally, it must be stressed that the meta-analysis carried out by Benoist d’Azy et al. in 2016 [164] demonstrated an increase in oxidative stress in both the serum and aqueous humor of chronic glaucoma patients, highlighting MDA as one of the best biomarkers of systemic oxidative stress in serum, and concluding that the increase in antioxidant markers may play a protective role within the eye against oxidative stress.

• Autoimmunity-related markers

In a pioneering 1998 study, Wax et al. [165] described the presence of elevated serum levels of autoantibodies to 60-kD bacterial and human heat-shock proteins in patients with glaucoma, motivating the search for alterations of the cellular immune system in patients with glaucoma. The analysis of subsets of T cells, in addition to inflammatory markers, in peripheral blood from POAG or NTG patients in comparison to age-matched control subjects showed an increase in CD8^+^HLA-DR^+^ and CD3^+^CD8^+^ lymphocytes in patients with NTG or NTG and POAG, respectively, when compared to controls. Moreover, higher levels of the soluble IL-2 receptor (sIL-2R) were determined in both POAG and NTG compared to non-glaucomatous individuals [166]. More recently, the analysis of T-cell subset distribution detected a glaucoma-related shift, since decreased frequency of CD4^+^ (or CD8^+^)/CD25^+^/FoxP3^+^ was determined in POAG patients when compared to controls. Likewise, CD4^+^ T cells presented a greater stimulation response (threefold) in glaucomatous samples, together with proinflammatory cytokine secretion, proposing the T-cell subset imbalance as a candidate biomarker of autoimmune susceptibility in glaucoma [167]. Similarly, other studies provided additional evidence for the involvement of the immune system in glaucoma, including the upregulation of γ-enolase [168], GST [169], anti-phosphatidylserine [170], and glycosaminoglycans [171] in serum.

The autoimmune role in the pathogenesis of glaucoma was later explored by comparing the antibody profiles against optic nerve antigens in patients from the USA and Germany with POAG (n = 40), with NTG (n = 40), and control subjects (n = 40). The α-fodrin was identified as a new candidate antibody biomarker, further confirmed by ELISA (1.2–1.4-fold-change in glaucoma) [172]. Therefore, the study of systemic antibodies and serum markers of optic nerve damage could potentially be used as indicators of glaucoma. Tezel et al., in 2012 [173], conducted an antibody-based proteomics approach, identifying disease-related antigens via LC–MS/MS. An additional validation step carried out via ELISA confirmed the upregulation in POAG patients, when compared to control subjects, of the candidate biomarkers apoptosis-inducing factor (AIF, 4.3-fold), cyclic AMP-responsive element-binding protein (CREB-binding protein, 4.3-fold), ephrin type-A receptor (7.8-fold), and huntingtin (4.1-fold). In the same year, using a targeted antigen microarray, Boehm et al. [174] were able to differentiate the sera of glaucoma patients with POAG from non-glaucomatous controls based on antibody profiles, with a sensitivity and specificity of 93%.

Beutgen et al., in 2019 [175], explored the alterations in the serological autoantibody profile by targeting antigens in the trabecular meshwork as biomarkers to support the early detection of POAG. The authors proposed a comprehensive approach based on serological proteome analysis for the discovery of autoantibodies (2D-PAGE, WB, immunoblot, and LC–MS/MS) and protein microarray analysis for their validation in an additional cohort (105 individuals). The candidates caldesmon (CALD1), phosphoglycerate mutase 1 (PGAM1), and voltage-dependent anion-selective channel protein 2 (VDAC2) were significantly higher in POAG patients when compared to control subjects, establishing a panel with the additional heat-shock protein 60 Kda (HSPD1) and VIM, reaching 81% sensitivity and 93% specificity in the diagnosis of glaucoma. Later, the same group carried out an exploratory analysis of pathways involving naturally immunogenic proteins and POAG-specific alterations, split into a discovery phase (30 POAG and 30 controls) by immunoprecipitation and LC–ESI–MS/MS, and a validation phase (120 POAG and 120 CT) by protein microarray, using serum and trabecular meshwork cell lines. Of the 106 potential autoantigens identified, the levels of antibodies to threonine-tRNA ligase (TARS), C1QBP, and paraneoplastic antigen Ma2 (PNMA2) were significantly higher in POAG patients compared to controls [176]. Finally, Shin et al., in 2020 [177], explored the serum of Korean OAG patients with NTG or HTG in a targeted approach using commercial enzyme immunoassays. Both glaucoma types showed higher levels of myelin basic protein (MBP), and NTG presented lower levels of anti-α-fodrin antibody, when compared with control individuals—which is contrary to the findings of a previous study [172]. Furthermore, the NTG group showed higher serum levels of anti-Sjögren’s-syndrome-related antigen A or antigen B (anti-SSA or anti-SSB) and lower levels of anti-α-fodrin IgG/IgA than the HTG group. The global data were used to determine the discriminating power between control and glaucoma subjects, proposing the MBP, anti-SSA, anti-SSB, and anti-α-fodrin IgG/IgA as useful candidate biomarkers for diagnosis and clinical differentiation within groups.

• Inflammatory-related markers

Novel inflammatory-related biomarkers are being evaluated at the systemic level in relation to glaucoma. For example, the nonapeptide thymulin—a hormone with immunoregulatory and anti-inflammatory functions—was found to be significantly elevated (2.6-fold) in the plasma of POAG patients when compared with the control group [178]. Similarly, the plasma levels of VEGF and the von Willebrand factor (vWf) were significantly elevated in patients with NTG and POAG compared to healthy controls, while the concentration of the soluble receptor for VEGF (sFlt-1) was significantly lower in the glaucomatous groups, which may be associated with abnormal vascular permeability and endothelial damage/dysfunction [179]. Moreover, acetylcholinesterase (AChE) was significantly increased (1.3-fold) in the red blood cells of POAG patients compared with control individuals, suggesting alterations in membrane integrity [180]. A group of POAG patients presented reduced levels of docosahexaenoic acid phosphatidylcholine (DHA-PC) and choline plasmalogens (PlsC), showing selective loss of some individual phospholipid species in red blood cell membranes [181]. Several vasoactive peptides have been proposed to be involved in glaucoma, including ET-1, the levels of which were previously observed to be increased in the aqueous humor [55]. Hence, the multiparametric analysis of plasma from POAG, NTG, and control subjects showed significantly higher levels of ET-1 and Hcy in both glaucomas, while lower vitamin E levels were found in NTG when compared with POAG or control subjects [182]. Similar results were recently obtained from the plasma of POAG patients for ET-1 (1.3-fold POAG vs. CT), indicating possible vascular endothelial dysfunction [183].

Just as in intraocular fluids, alterations of inflammatory cytokines have been observed in the peripheral sera of glaucoma patients. Significantly higher serum levels of IL-4, IL-6, and IL-12 (p70), along with lower TNF-α, were determined in POAG patients compared to controls, suggesting abnormal immune environments contributing to glaucoma [184]. Conversely, two recent studies obtained by ELISA found significantly elevated levels of TNF-α in the plasma of both POAG and PEXG patients when compared to control subjects, providing further evidence of systemic inflammation, but its potential value as a candidate biomarker of glaucoma needs further investigation [185,186]. In addition, the levels of neutrophil-to-lymphocyte ratio (NLR) and platelet-to-lymphocyte ratio (PLR)—commonly used as subclinical inflammation markers—were found to be upregulated in patients with POAG and OHT when compared to control subjects, although with low sensitivity (65%) and specificity (65%) when discriminating glaucoma disease [187]. Moreover, NLR, white blood cells, and neutrophils were upregulated in PACG (n = 771) patients, while the lymphocyte-to-monocyte ratio (LMR) was found to be downregulated in glaucoma patients when compared to control subjects (n = 770). These parameters provided AUCs of 0.719 and 0.699 for NLR and LMR, respectively, with elevated diagnostic potential for this subtype of glaucoma [188]. Recently, the monocyte-count-to-high-density-lipoprotein ratio (MHR)—a marker of inflammation and oxidative stress in cardiovascular diseases—has been studied in the blood of PEXG (n = 21) and PES (n = 21) patients, observing a significant increase in both groups when compared to control subjects, probably related to systemic inflammation [189]. Furthermore, a similar trend was observed in NVG patients, secondary to RVO or DR, in which upregulation of blood cells, neutrophils, and NLR and downregulation of LMR were observed when compared to control subjects. Overall, NLR seems to be specifically altered in several subsets of glaucoma, making it a potential inflammation biomarker [190].

Following the analysis of members of the neurotrophin family—which may be involved in the glaucomatous neurodegeneration—in ocular fluids, the participation of the neurotrophic factor BDNF in the regulation of blood flow was determined via ELISA in the sera of early-stage POAG patients. Thus, BDNF was significantly lower in glaucoma patients (0.7-fold) when compared with controls [191], with an opposite trend to that observed by the same authors in tear film [139]. Later, serum BDNF and nerve growth factor (NGF) were determined in POAG patients, observing significantly lower levels when compared to control subjects [192]. In a recent study, BDNF was analyzed via ELISA in the serum of Japanese POAG and NTG patients and control subjects, observing significantly lower levels in both glaucoma groups than in the controls [193], which merits further investigation of this factor as a potential circulating biomarker for the early detection of glaucoma. On the other hand, neuroglobin—a hypoxic sensor and initiator of signal transduction involving oxidative and hypoxic pathways—was determined in POAG patients, showing high upregulation (5.2-fold) when compared with control subjects, with an AUC of 0.82, suggesting neuroglobin as an interesting candidate biomarker for the diagnosis of glaucoma [194].

González-Iglesias et al., in 2014 [195], conducted a comprehensive study consisting of a two-step approach including a differential proteomic analysis and an ELISA screening. The authors identified alterations in serum proteins when comparing POAG and PEXG patients and healthy controls via differential proteomics analysis. A panel of the top 17 ranked distinct proteins, the signaling network of which was correlated with immunological and inflammatory response pathways, was confirmed via immunoassays (ELISA), with APOA4 classifying the groups with 81% correct assignment. Most of the proteins were identified as being overexpressed in the sera of glaucoma patients, with the exceptions of Ig gamma-2 chain C region (IGHG2) and C4A. The identified altered proteins support the finding that changes to immune and inflammatory pathways occur at a systemic level in glaucoma. In addition, systemic alterations of complement component 3 (C3) were also identified in PACG patients, with significantly lower plasma C3 levels in PACG when compared to controls, especially in older women, suggesting a role of this protein in the etiology and/or progression of glaucoma [196].

• Extracellular-matrix- and lipid-metabolism-related markers

The abnormal accumulation of the extracellular matrix in glaucomatous eyes attracted attention to the study of extracellular components at the systemic level. The extracellular matrix is modified by MMPs and their TIMPs, prompting an upregulation of membrane-type 1-MMP (MT1-MMP) in the NTG patients [197]. The composition of exfoliation materials includes the highly glycosylated and crosslinked glycoprotein/proteoglycan complex, which fostered the investigation of alterations to the glycosaminoglycans heparan sulfate (HS) and chondroitin sulfate (CS) in the sera of PEXG, PES, POAG, and control subjects via ELISA. Higher levels of HS and CS were observed in both the PEXG and PES groups compared to those without exfoliation material, although the AUC based on the ROC curves reached 0.62 for HS or CS (PEXG vs. CT), indicating low discriminating power [198]. Levels of 3α-hydroxysteroid dehydrogenase (3α-HSD) activity determined in leukocytes from POAG patients were lower than those of the controls [199]. Moreover, the multicatalytic endopeptidase 20S proteasome α-subunit was measured in the leukocytes of HTG, NTG, and control subjects, observing a 3.4-fold increase in the glaucomatous patients when compared to healthy controls, which eventually may be used as a prognostic marker for glaucomatous damage [200]. Finally, the serum lipid status has also been studied at the systemic level in glaucoma patients, considering its relationship with vascular alterations. Specifically, in a targeted approach carried out in PEXG, PES, and control subjects, the analysis of cholesterol, high-density lipoprotein (HDL), low-density lipoprotein (LDL), and triglyceride levels revealed a significantly higher concentration of LDL in both the PEXG and PES groups [201].

#### 3.4.2. Metabolite-Based Biomarkers

The study of the serum metabolite profiles of glaucomatous patients may contribute to the identification of important indicators of physiological and pathological states, to determine the mechanism of disease occurrence and progression, and to identify early and differential metabolic markers. In this vein, considering the suggested implication of Hcy in glaucoma, the quantification of this amino acid, along with vitamin B12 and folic acid, was carried out in the plasma of PEXG, POAG, and control subjects. Interestingly, higher levels of fasting plasma Hcy were observed in PEXG patients when compared with both POAG and control subjects, but no differences were obtained for vitamin B12 or folic acid [202]. Moreover, the plasma levels of hydrogen sulfide, Hcy, and L-cysteine (Cys) were determined via a targeted analysis in POAG (n = 42), NTG (n = 20), OHT (n = 52), and control (n = 78) individuals, showing lower hydrogen sulfide concentration in POAG than OHT or control subjects, and higher levels of Hcy and Cys in POAG and NTG when compared with the control group. However, the AUC obtained (0.642–0.721 POAG vs. CT) provided poor diagnostic values, without sufficient sensitivity to be used as reliable biomarkers for glaucoma [203].

The intermediate in the tricarboxylic acid cycle—citrate—was measured via ionic chromatography in the urine and plasma of glaucomatous individuals (n = 21), observing lower levels of this metabolite when compared to control subjects, with a sensitivity of 66.7% and a specificity of 71.4% in discriminating glaucoma [204]. Similarly, plasma citrate levels were significantly lower in school-aged children diagnosed with glaucoma, compared to control subjects, but not for urine citrate levels, suggesting the consideration of plasma citrate as a glaucoma biomarker in the pediatric population [205]. Considering that uric acid may exert a protective effect against oxidative damage, this metabolite was quantified in the sera of POAG (n = 163) and control (n = 103) subjects, observing significant downregulation of both uric acid and uric acid/creatinine ratio in glaucoma [206]. On the other hand, in view of the vascular component observed in glaucoma, a targeted study confirmed the significant elevation of the isomeric derivatives of l-arginine—i.e., asymmetric dimethylarginine (ADMA) and symmetric dimethylarginine (SDMA), acting as endogenous inhibitors of NOS—in the sera of advanced glaucoma subjects (n = 211), when compared to the control group (n = 295) [207]. Furthermore, the suggested role of lipids in glaucoma fostered the study of blood fatty acids in POAG patients (n = 10) and their respective healthy siblings (n = 8), observing lower plasma levels of eicosapentaenoic fatty acid (EPA), DHA, and total ω3 long-chain polyunsaturated fatty acid (LCPUFA) in the glaucoma group [208]. Later, the use of GC–MS for a targeted metabolomics analysis of serum samples from PACG subjects showed higher levels of palmitoleic acid (PA, 1.2-fold), gamma-linolenic acid (GLA, 1.7-fold), arachidonic acid (ARA, 0.8-fold), and adrenic acid (1.3-fold) compared to control subjects [209]. The analysis of oxidation products of linoleates—one of the most abundant polyunsaturated fatty acids—was accomplished by the serum quantification of hydroxylinoleate (HODE) and hydroxyarachidonate (HETE) isomers in POAG (n = 198, divided into NTG and HTG) and control subjects (n = 119), observing significantly higher levels of both metabolites in glaucomatous patients, with potential clinical significance for the screening of the disease [210].

The comparison analysis of blood plasma between POAG (n = 72) and healthy control patients (n = 72), using LC–MS/MS, found significant differences in specific metabolic processes involving palmitoylcarnitine, hydroxyergocalciferol, sphingolipids, vitamin-D-related compounds, and terpenes, probably indicative of mitochondrial dysfunction and energy metabolism changes, although the obtained fold changes were not indicated [211]. Leruez et al., in 2018 [212], applied a targeted quantitative metabolomics approach to determine the plasma metabolomics signatures of POAG patients. Among the 150 metabolites quantified, 18 discriminated both groups, belonging to the carbohydrate, acyl-carnitine, phosphatidylcholine, amino acid, and polyamine families. Moreover, specific deficiency of the polyamines involved in the protection of RGCs—spermidine and spermine—was detected in POAG, similar to previous observations in aqueous humor [105]. Furthermore, specific analysis of amino acids and lactate determined systemic alterations in NTG patients compared to age-matched controls, with reduced lactate and total amino acids and elevated valine and ornithine in the glaucoma cohort [213]. Recently, an untargeted metabolomics study using LC–(high-resolution)-MS (LC–HRMS) carried out in the plasma of POAG (n = 34) and control (n = 30) individuals reported the dysregulation of nine metabolites in POAG patients when compared to control subjects, including decreased concentrations of nicotinamide, hypoxanthine, xanthine, and 1-methyl-6,7-dihydroxy-1,2,3,4-tetrahydroisoquinoline, and increased levels of N-acetyl-L-leucine, arginine, r-glycerol 1-myristate, 1-oleoyl-RAC-glycerol, and cystathionine. The prediction accuracy reached 93.01% for controls and 82.43% for glaucoma patients, showing mitochondrial, nucleotide, and amino acid impairment as contributing factors to the pathogenesis of glaucoma [214].

**Table 4:** Candidate glaucoma biomarkers identified in blood, serum, or plasma (in addition to other fluids). The study, the strategy followed, the main analytical techniques used, the fold change of markers when specified, the number of subjects, and the disease of the recruited patients are indicated.

Study	Fluid/Tissue	Strategy	Analytical Technique	List of Candidate Biomarkers (Fold-Change vs. Controls) ^1^	Samples
Tezel et al., 1999 [171]	Serum	Targeted proteomics	WB and ELISA	Up ^2^: HS (1.8-fold NTG vs. CT, 1.5-fold NTG vs. POAG), CS (2.2 NTG vs. CT, 1.5-fold NTG vs. POAG)	60 NTG, 36 POAG, 20 CT
Yang et al., 2001 [169]	Serum	Untargeted analysis (discovery) and targeted analysis (validation)	WB, 2DGE, and LC–ESI–MS (discovery) and ELISA (validation)	Up: anti-GST antibody (1.4-fold POAG vs. CT, 1.3-fold NTG vs. CT)	40 NTG, 25 POAG, 25 CT
Lip et al., 2002 [179]	Plasma	Targeted proteomics	ELISA	Up: VEGF (1.8-fold POAG vs. CT, 2.7-fold NTG vs. CT, 1.5-fold NTG vs. POAG)	24 POAG, 26 NTG, 26 CT
				Down ^3^: sFlt-1 (0.2-fold POAG vs. CT, 0.6-fold NTG vs. CT)	
Golubnitschaja et al., 2004 [197]	Blood (leukocytes)	Targeted proteomics	WB	Up: MT1-MMP (Unknown-fold)	6 NTG, 6 CT
Emre et al., 2005 [183]	Plasma	Targeted proteomics	Radioimmunoassay	Up: ET-1 (1.3-fold)	16 POAG, 15 CT
Gherghel et al., 2005 [150]	Blood	Targeted proteomics	Spectrophotometric (enzymatic)	Down: GSH (0.7-fold)	21 POAG, 34 CT
Yildirim et al., 2005 [149]	Blood	Targeted analysis	Spectrophotometric (analysis of activity)	Up: Plasma MDA (2.3-fold)	40 POAG, 60 CT
Grus et al., 2006 [172]	Serum	Untargeted analysis (discovery) and targeted analysis (validation)	WB (discovery) and ELISA (validation)	Up: α-fodrin (1.4-fold NTG vs. CT, 1.2-fold NTG vs. POAG)	40 POAG, 40 NTG, 40 CT
Acar et al., 2009 [181]	Red blood cells	Targeted proteomics	LC–ESI–MS/MS	Down: DHA-PC	31 POAG, 16 CT
Huang et al., 2010 [184]	Serum	Targeted proteomics	ELISA	Up: IL-4 (1.5-fold), IL-6 (1.5-fold), IL-12p70 (1.4-fold)	32 POAG, 26 CT
				Down: TNF-α (0.9-fold)	
Engin et al., 2010 [151]	Serum	Targeted analysis	Spectrophotometric (Enzymatic) and HPLC–UV	Up: MDA (1.2.fold), serine (1.2-fold), TF (1.1-fold), vitamin A (1.2-fold), vitamin E (1.5-fold)	160 glaucoma (type non-indicated), 31 CT
				Down: TAC (0.9-fold), SOD (0.9-fold), GPx (0.8-fold)	
Sorkhabi et al., 2011 [162]	Serum and aqueous humor	Targeted analysis	ELISA and spectrophotometric	Up: 8-OHdG (2.3-fold in aqueous humor, 1.3-fold in serum)	15 POAG, 13 PEXG, 27 CT
				Down: TAS (0.7-fold in aqueous humor, 0.8-fold in serum)	
Chang et al., 2011 [159]	Serum	Targeted analysis	Spectrophotometric	Up: MDA (1.2-fold), conjugated diene (1.1-fold), AOPP (1.1-fold), protein carbonyl (1.2-fold), ischemia-modified ALB (1.05-fold), 8-OHdG (1.1-fold).	50 PACG, 50 CT
Majsterek et al., 2011 [152]	Red blood cells	Targeted analysis	Spectrophotometric (analysis of activity)	Down: CAT (0.6-fold), SOD (0.6-fold), GPx (0.8-fold).	20 POAG, 20 CT
Ghaffariyeh et al., 2011 [191]	Serum	Targeted proteomics	ELISA	Down-regulated: BDNF (0.7-fold)	25 POAG, 25 CT
Zanon-Moreno et al., 2013 [153]	Plasma	Targeted analysis	LC–UV, LC(RP)–electrochem, and spectrophotometric	Up: GPx (1.5-fold)	250 POAG, 250 CT
				Down: vitamin E (0.9-fold)	
Abu-Amero et al., 2013 [161]	Plasma	Targeted analysis	Spectrophotometric (enzymatic)	Down: TAS (0.5-fold)	139 POAG, 148 CT
López-Riquelme et al., 2014 [182]	Plasma	Targeted analysis	ELISA, chemiluminescence immunoassay, HPLC–UV	Up: ET-1 (1.9-fold POAG vs. CT, 1.4-fold NTG vs. CT), Hcy (1.3-fold POAG vs. CT, 1.1-fold NTG vs. CT)	48 POAG, 15 NTG, 75 CT
				Down: Vitamin E (0.7-fold NTG vs. CT, 0.7-fold NTG vs. POAG)	
González-Iglesias et al., 2014 [195]	Serum	Untargeted proteomics (discovery) and targeted proteomics (validation)	2D-DIGE, LC–MS/MS, and MALDI-TOF/TOF (discovery) and ELISA (validation)	Up: APOA4 (2.7-fold POAG vs. CT, 1.5-fold PEXG vs. CT, 1.8-fold POAG vs. CT), C3 (1.5-fold POAG vs. CT, 1.4 fold POAG vs. PEXG), TTR (1.8-fold POAG vs. CT, 1.5-fold POAG vs. PEXG), TF (1.7-fold POAG vs. CT, 1.5-fold POAG vs. PEXG), VTN (2.2-fold POAG vs. CT, 1.6-fold PEXG vs. CT), fibulin-1 (FBLN1, 1.9-fold POAG vs. CT, 1.5-fold PEXG vs. CT), APOA1, 1.3-fold POAG vs. CT), alpha-1 antitrypsin (SERPINA1, 1.5-fold POG vs. CT, 1.3-fold POAG vs. PEXG), CFH (1.3-fold POAG vs. CT), apolipoprotein L1 (APOL1, 1–4-fold POAG vs. CT), ficolin-3 (FCN3, 1.3-fold POAG vs. CT, 1.3-fold POAG vs. PEXG)	Discovery: 53 POAG, 45 PEXG, 41 CT. Validation: 20 POAG, 14 PEXG, 17 CT.
				Down: IGHG2 (0.7-fold POAG vs. CT, 0.7-fold PEXG vs. CT), C4A (0.8-fold POAG vs. CT)	
Ozgonul et al., 2016 [187]	Blood	Targeted analysis	Spectroscopy (hematology and chemistry analyzers)	Up: NLR (1.2-fold POAG vs. CT, 1.1-fold OHT vs. CT)	84 POAG, 94 OHT, 80 CT
Li et al., 2017 [196]	Plasma	Targeted proteomics	Immunoturbidimetry	Down: C3 (0.9-fold PACG vs. CT, 0.9-fold female PACG vs. female CT)	237 PACG, 158 CT
Oddone et al., 2017 [192]	Serum	Targeted proteomics	ELISA	Down: BDNF (0.8-fold), NGF (0.7-fold)	45 POAG, 15 CT
Li et al., 2017 [188]	Blood	Targeted analysis	Biochemical analyzer	Up: White blood cell (1.05.fold), neutrophil (1.2-fold), NLR (1.4-fold).	771 PACG, 770 CT
				Down: LMR (0,7-fold)	
Rokicki et al., 2017 [154]	Serum	Targeted proteomics	Spectrophotometric	Up: Lipofuscin (1.2-fold), MDA (1.5-fold), TOS (2.8-fold).	30 POAG, 25 CT
				Down: Total SOD activity (0.8-fold), mitochondrial SOD (0.8-fold)	
Kondkar et al., 2018 [185]	Plasma	Targeted proteomics	ELISA	Up: TNF-α (2.0-fold)	51 POAG, 88 CT
Kondkar et al., 2018 [186]	Plasma	Targeted proteomics	ELISA	Upregulated: TNF-α (6.0-fold)	49 PEXG, 88 CT
Yaz et al., 2019 [155]	Serum	Targeted analysis	Spectrophotometric	Up: MDA (5.0-fold PEXG vs. CT, 2.1-fold PES vs. CT, 1.3-fold PEXG vs. PES), GSH (1.6-fold PEXG vs. CT, 1.6-fold PES vs. CT)	58 PEXG, 47 PES, 134 CT
				Down: SOD activity (0.3-fold PEXG vs. CT, 0.3-fold PES vs. CT), CAT activity (0.6-fold PEXG vs. CT, 0.5-fold PES vs. CT), nitric oxide (0.8-fold PEXG vs. CT, 0.7-fold PEXG vs. PES)	
Yang et al., 2019 [167]	Blood	Targeted analysis	Flow cytometry and ELISA	Up: IL-1β (unknown-fold), IFN-γ (unknown-fold), TNF-α (unknown-fold)	32 POAG, 21 CT
Karakurt et al., 2019 [158]	Serum	Targeted analysis	Bioanalyzer and spectrophotometric	Up: Ischemia-modified ALB (1.2-fold), disulfide (1.3-fold), disulfide/native thiol (1.1-fold), disulfide/total thiol (1.1-fold)	70 POAG, 87 CT
				Down: Total thiol (0.8-fold), native thiol (0.8-fold)	
Maric et al., 2019 [198]	Serum	Targeted analysis	ELISA	Up: Serum HS (1.2-fold PEXG vs. CT, 1.5-fold PEXG vs. POAG), CS (1.2-fold PEXG vs. CT)	47 PEXG, 43 POAG, 22 PES, 53 CT
Igarashi et al., 2020 [193]	Serum	Targeted proteomics	ELISA	Down: BDNF (0.6.fold POAG vs. CT, 0.5-fold NTG vs. CT, 1.3-fold POAG vs. NTG)	16 POAG, 11 NTG, 51 CT
Shin et al., 2020 [177]	Serum	Targeted proteomics	ELISA	Down: Anti-α-fodrin antibody (IgG, 0.6-fold NTG vs. CT, 0.4-fold NTG vs. HTG), Anti-α-fodrin antibody (IgA, 0.6-fold NTG vs. HTG)	17 NTG (OAG), 7 HTG (OAG), 17 CT
Li et al., 2020 [160]	Serum	Targeted analysis	Spectrophotometric (enzymatic)	Up: MDA (5.5-fold PACG vs. CT), hydrogen peroxide (2.2-fold PCAG vs. CT)	94 PACG, 89 CT
				Down: SOD (0.8.fold PACG vs. CT), TAS (0.8-fold PACG vs. CT)	
Kondkar et al., 2020 [163]	Plasma	Targeted analysis	ELISA	Up: 8-OHdG (1.4-fold)	50 POAG, 45 CT
Gulpamuk et al., 2020 [157]	Serum	Targeted proteomics	Spectrophotometric (enzymatic)	Up: Ischemia-modified ALB (1.1-fold POAG vs. CT)	30 POAG, 30 OHT, 30 CT
				Down: Native thiol (0.9-fold POAG vs. CT, 0.9-fold OHT vs. CT), total thiol (0.9-fold POAG vs. CT, 0.9-fold OHT vs. CT)	
Zhang et al., 2021 [190]	Blood	Targeted analysis	Bioanalyzer	Up: White blood cell (1.3-fold NVG-RVO vs. CT, 1.2-fold NVG-DR vs. CT), neutrophil (1.4-fold NVG-RVO vs. CT, 1.3-fold NVG-DR vs. CT), NLR (1.3-fold NVG-RVO vs. CT, 1.3-fold NVG-DR vs. CT).	38 NVG (secondary to RVO), 46 NVG (secondary to DR), 59 CT
				Down: LMR (0.7-fold NVG-RVO vs. CT, 0.7-fold NVG-DR vs. CT)	
Ren et al., 2006 [208]	Plasma and red blood cells	Targeted metabolomics	GC–MS and spectrophotometry	Down: DHA (0.8-fold in red cell colline phosphoglycerides, 0.7-fold in plasma)	10 POAG, 8 CT
Fraenkl et al., 2011 [204]	Plasma and urine	Targeted metabolomics	Ion chromatography	Down: Citrate (0.8-fold in plasma)	12 NTG, 8 POAG, 1 PEXG, 21 CT
Tranchina et al., 2011 [202]	Plasma	Targeted metabolomics	Competitive chemiluminescent enzyme immunoassay	Up: Hcy (1.3-fold PEXG vs. CT, 1.2-fold PEXG vs. POAG).	36 PEXG, 40 POAG, 40 CT
Michalczuk et al., 2017 [205]	Plasma and urine	Targeted metabolomics	Enzymatic	Down: Citrate (0.8-fold in plasma, 0.6-fold urine)	34 glaucoma, 34 CT
Lin et al., 2020 [203]	Plasma	Targeted metabolomics	Spectroscopy (Spectrophotometry or LC-fluorimeter)	Up: Hcy (1.1-fold POAG vs. CT, 1.2-fold NTG vs. CT), Cys (1.1-fold POAG vs. CT, 1.2-foldNTG vs. CT)	42 POAG, 20 NTG, 52 OHT, 78 CT
Nzoughet et al., 2020 [214]	Plasma	Untargeted metabolomics	LC–HRMS	Up: N-acetyl-L-leucine (1.8-fold), 1-oleoyl-rac-glycerol (1.6-fold), arginine (1.3-fold), rac-glycerol 1-myristate (1.3-fold), cystathionine (1.6-fold)	34 POAG, 30 CT
				Down: Nicotinamide (0.6-fold), hypoxanthine (0.6-fold), 1-methyl-6,7-dihydroxy- 1,2,3,4-tetrahydroisoquinoline (0.5-fold), xanthine (0.7-fold)	

^1^ Comparison with other groups is indicated in brackets; ^2^ Up: upregulated; ^3^ Down: downregulated.

## 4. Outlook and Perspectives

**Figure 2:**
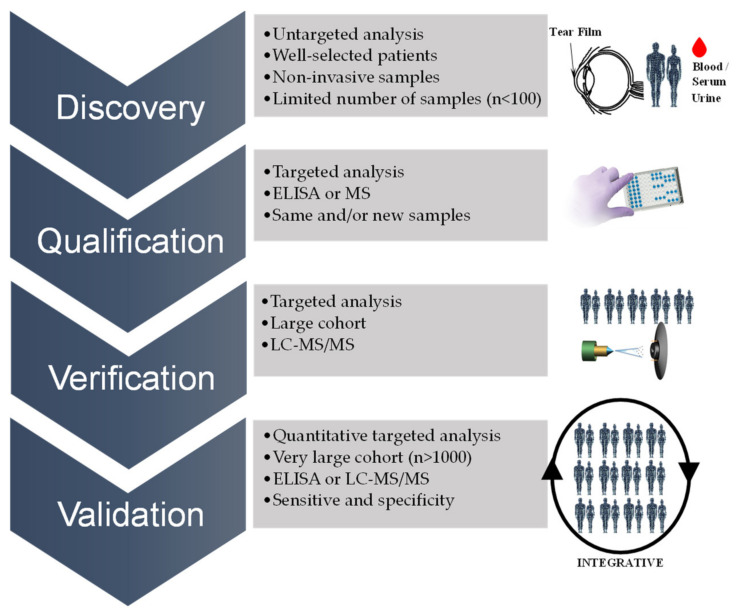
Pipeline of discovery and validation of glaucoma biomarkers prior to their clinical implementation.

Glaucoma is a leading cause of blindness worldwide, which encompasses a complex group of disorders that are multigenic and multifactorial in origin. Improving the follow-up and treatment of glaucoma is crucial to develop a much more sensitive and specific method of early detection based in molecular biomarkers, especially bearing in mind that this disease might even be asymptomatic, and that the clinical diagnosis is usually realized at advanced stages, when important and irreversible loss of visual field occurs. From our point of view, considering the revised literature, the conventional workflow in the identification and validation of glaucoma biomarkers must be strictly designed, as shown in Figure 2. The biomarker development pipeline must involve four differential steps, i.e., discovery, qualification, verification, and validation phases. The discovery phase, intended to identify proteins as potential biomarkers, should include a limited number of samples (n < 100) from exhaustively phenotyped patients and subjected to untargeted analysis using cutting-edge analytical platforms. The qualification phase is critical to confirm the differential abundance of altered molecules, requiring an alternative targeted analysis based on ELISA or preferentially quantitative MS approaches. The verification (or proof-of-concept) phase aims to evaluate the characteristics of the candidate proteins in a larger cohort of patients by means of targeted proteomics or metabolomics of high accuracy and sensitivity (e.g., LC–MS/MS), establishing a limited panel of disease markers. Finally, the validation of biomarkers pursues their clinical accreditation through the use of robust, reproducible, and quantitative analytical technologies on a high number of samples (n > 1000) from different cohorts. Those molecules that have been successfully accredited by this multistep approach are eligible for transfer to large-scale qualifying clinical studies.

Sample selection is a key feature during the discovery of candidate biomarkers. The analysis in readily available biological fluids of glaucoma patients—i.e., those that do not require invasive surgery—provides a preferred alternative to the high risk involved in the extraction of fluids or tissues through invasive surgery of the eye. Moreover, a non-invasive test in tears, sera, or urine from patients with glaucoma could have a significant impact on the early diagnosis, prognosis, clinical prediction, and management of this disease. An interesting strategy for the discovery of glaucoma biomarkers would include proteomics and/or metabolomics studies on the sera of families with Mendelian inheritance of glaucoma, including those with myocilin mutations causing 3–4% of POAG. This type of study may shed light on the specific molecules altered and common pathways affected by the onset of the disease. Additionally, in this step—and as previously discussed—glaucoma medications induce alterations in eye fluids or tissues, which may mask specific changes in the proposed biomarkers, such as inflammatory- or immune-response-related molecules. Considering that this is probably most dramatic in long-term treated patients, specific considerations must be addressed in the experimental design of discovery, verification, and validation studies. A preferred alternative should tackle the selection of untreated patients, once diagnosed, although this is certainly problematic. On the other hand, it must be stressed that a single biomarker seems very unlikely to be of much help in the detection of glaucoma due to the etiological heterogeneity of the disease, the existence of different subtypes, and the genetic variability among patients. Rather, a panel of biomarkers may provide more useful information for clinical prediction, including better sensitivity and specificity.

The accreditation of candidate biomarkers, from the qualification to validation phases, requires the use of quantitative and reproducible analytical techniques in large-scale multiplexed screenings permitting the simultaneous quantitation of several proteins or metabolites in a biological sample, with traceability assurance and proper normalization. Accreditation studies involve the application of advanced analytical technologies capable of rapidly and accurately measuring the absolute concentration of candidate markers in readily accessible biological samples [215]. Although, in the past, antigen/antibody assays (e.g., ELISA, multiplexed immunoassays, etc.) have been commonly used for the quantitative determination of one target at a time, this approach is expensive, unreliable, and unpractical for the simultaneous validation of multiple markers in hundreds of samples. In recent years, a powerful technology based on mass spectrometry analysis—known as multiple-reaction monitoring (MRM-MS)—has emerged and been applied successfully for the simultaneous quantification of manifold proteins in a single assay without the need to use protein-specific antibodies [216,217]. The application of this analytical technique, or other approaches preferentially based in molecular mass spectrometry, may contribute to the verification of any proposed panel of candidate glaucoma biomarkers. Nevertheless, there still remains the final clinical validation phase to eliminate false positivity and calculate the biomarker sensitivity and specificity through the targeted analysis of candidate molecules in large and heterogeneous populations [18]. Thus, recruitment of a new cohort of glaucoma patients with different origins, types, stages, and treatments will improve the statistical power and contribute to reducing false positivity and increasing sensitivity and specificity for clinical diagnosis.

Ultimately, the great abundance of data yielded from the spectrum of the “omics cascade” and the development of new bioinformatics tools opens new perspectives in order to achieve an integrative approach to decipher their biological meaning in relation to glaucoma. In addition, the establishment of more complete databases from populations around the world will benefit the advances in this field. However, data integration is still under development due to the different formalisms contained in each of the omics platforms, to which the different timescales of gene expression, protein synthesis, and metabolite production must be added. Future studies should undertake a multiomics approach at the genomic, transcriptomic, proteomic, and/or metabolomic levels in individual subjects in order to further integrate combined data using cutting-edge bioinformatics tools (data processing, clustering, dynamics, integration of the various omics levels, etc.) and systems biology [218].

## 5. Conclusions

The identification of molecular biomarkers of glaucoma may contribute to its early diagnosis and prediction of its prognosis, help to evaluate the therapeutic effectiveness of drugs, and also foster new therapeutic interventions. There is a strong need for a safe biomarker that is easy to measure, reproducible, and virtually useful in different cohorts. However, hundreds of candidate biomarkers (>450) have been proposed to date, but none of them have been validated in large cohorts from populations of different origin, so they have not yet reached the clinic. Overall, although much progress has been accomplished to date, years of research are still needed in order to truly translate candidate glaucoma biomarkers into clinical practice.

## Data Availability

Not applicable.

## Supplementary Materials

The following are available online at https://www.mdpi.com/article/10.3390/biology10080763/s1: Table S1: Candidate glaucoma biomarkers identified in aqueous humor (in addition to other fluid); Table S2: Candidate glaucoma biomarkers identified in the retina, optic nerve, vitreous body, or trabecular meshwork; Table S3: Candidate glaucoma biomarkers identified in tear film; Table S4: Candidate glaucoma biomarkers identified in blood, serum, or plasma (in addition to other fluids).
