# Supplementary material for: Candidate Glaucoma Biomarkers: From Proteins to Metabolites, and the Pitfalls to Clinical Applications
Source: Biology (Basel). 2021 Aug 10;10(8):763. doi: 10.3390/biology10080763 (PMC8389649; doi:10.3390/biology10080763)
Supplement: Supplementary file 1 [file biology-10-00763-s001.zip › biology-1321259-supplementary.pdf]

biology

MDPI

Review – Supplementary Material

Candidate Glaucoma Biomarkers: Proteins to Metabolites and the Pitfalls to Clinical Applications

Andrés Fernández-Vega Cueto [1,2], Lydia Álvarez [2], Montserrat García [1,2], Ana Álvarez-Barrios [2], Enol Artime [2], Luis Fernández-Vega Cueto [1,2,*], Miguel Coca-Prados [3] and Héctor González-Iglesias [1,2,*]

1. Instituto Oftalmológico Fernández-Vega, Avda. Dres. Fernández-Vega, 34, 33012 Oviedo, Spain; afvega89@gmail.com (A.F.V.C.); mgarciadiaz@fio.as (M.G.); h.gonzalez@fio.as (H.G.I.)
2. Instituto Universitario Fernández-Vega (Fundación de Investigación Oftalmológica, Universidad de Oviedo), 33012 Oviedo, Spain; l.alvarez@fio.as (L.A.); ana.alvarezbarrios1@gmail.com (A.A.B.); enol.artime@fio.as (E.A.)
3. Department of Ophthalmology and Visual Science, Yale University School of Medicine, New Haven, CT. 06510, USA; miguel.coca-prados@yale.edu (M.C.P.)

* Correspondence: h.gonzalez@fio.as (H.G.I.); lfvc@fernandez-vega.com (LF.V.C.), Tel.: +34-985-24-0141

Supplementary Material Description: The supplementary material summarizes the main results obtained in proteomics and metabolomics-based studies on glaucoma biomarkers, with specificity to eye tissue or fluid as well the analytical approach and the identified altered molecules. The fold-change, when possible, has been also determined. Table S1 illustrates the candidate markers observed in the analysis of aqueous humor samples from glaucoma patients. Table S2 contains the list altered molecules identified in retina, optic nerve, vitreous body or trabecular meshwork from glaucoma patients. Table S3 shows the list of proteins or metabolites obtained during the analysis of tear samples from patients with glaucoma. Table S4 recapitulates the candidate glaucoma biomarkers identified in blood, serum or plasma (in addition to other fluid).

Citation: Fernández-Vega Cueto, A.; Álvarez, L.; García, M.; Álvarez-Barrios, A.; Artime, E.; Fernández-Vega Cueto, L.; Coca-Prados, M.; González-Iglesias, H. Candidate Glaucoma Biomarkers: From Proteins to Metabolites, and the Pitfalls to Clinical Applications. *Biology* **2021**, *10*, 763. https://doi.org/10.3390/biology10080763

Academic Editors: Jacqueline Reinhard and Stephanie C. Joachim

Received: 15 July 2021
Accepted: 8 August 2021
Published: 10 August 2021

Publisher's Note: MDPI stays neutral with regard to jurisdictional claims in published maps and institutional affiliations.

Copyright: © 2021 by the authors. Licensee MDPI, Basel, Switzerland. This article is an open access article distributed under the terms and conditions of the Creative Commons Attribution (CC BY) license (http://creativecommons.org/licenses/by/4.0/).

1. Aqueous Humor

Table S1. Candidate glaucoma biomarkers identified in aqueous humor (in addition to other fluid). The study, the followed strategy, the main analytical techniques used, the fold-change of markers when specified, the number of subjects and the disease of recruited analyzed have been indicated.

Study	Fluid/Tissue	Strategy	Analytical Technique	List of candidate biomarkers (fold-change vs controls)[1]	Samples[2]
Tripathi et al., 1994 [1]	Aqueous Humor	Targeted Proteomics	ELISA	Up[3]: TGFβ2 (1.8-fold)	15 POAG, 10 CT
Tezel et al., 1997 [2]	Aqueous Humor and Plasma	Targeted Proteomics	RIA	Up: ET (1.05-fold in aqueous humor)	31 POAG, 24 CT
Vesaluom et al., 1998 [3]	Aqueous Humor	Targeted Proteomics	ELISA	Up: Plasma FN (3,1-fold PEXG vs CT, 2.1-fold PEXG vs POAG), cellular FN (∞-fold PEXG vs CT, ∞-fold PEXG vs POAG)	29 POAG, 26 PEXG, 13 CT
Kee et al., 1999 [4]	Aqueous Humor	Targeted Analysis	Zymography	Up: Gelatinase A (3.9-fold in POAG vs CT)	6 POAG, 4 CACG, 4 NTG, 14 CT
Ferreira et al., 2004 [5]	Aqueous Humor	Targeted Quantitative Analysis (Activity assay)	Spectrophotometry	Up: SOD (1.7-fold), GPx (3.0-fold)	24 POAG, 24 CT
Navajas et al., 2005 [6]	Aqueous Humor	Targeted Analysis	Noncompetitive fluorescence-based assay	Down[4]: Hyaluronic acid (0.5-fold)	22 POAG, 22 CT
Määttä et al., 2005 [7]	Aqueous Humor	Targeted Proteomics	ELISA	Up: MMP-2 (2.1-fold PEXG vs CT, 1.7-fold PEXG vs POAG, 2.0-fold PES vs CT), TIMP-2 (7.7-fold PEXG vs CT, 3.0-fold POAG vs CT, 6.0-fold PES vs CT)	15 POAG, 16 PEXG, 15 PES, 10 CT
Min et al., 2006 [8]	Aqueous Humor	Targeted-Proteomics	ELISA	Up: TGFβ2 (2.7-fold POAG vs CT, 2.3-fold NVG vs CT, 1.4-fold SOAG vs CT)	43 glaucoma (14 POAG, 14 NVG, 15 SOAG), 20 CT
Yu et al. 2007 [9]	Aqueous Humor	Targeted-Proteomics	ELISA	Up: TGFβ1 (control levels below detection limit), TGFβ2 (16-fold).	NVG, CT
Nolan et al. 2007 [10]	Aqueous Humor	Targeted-Proteomics	ELISA	Up: sCD44 (2.2-fold)	90 POAG, 124 CT
Joachim et al., 2007 [11]	Aqueous Humor	Untargeted Proteomics	SDS & LC-MS/MS	Up: VIM, HSP70.<br>Down: αB-crystallin	20 NTG, 20 CT
Joachim et al., 2007 [12]	Aqueous Humor	Untargeted Proteomics	WB & MALDI-MS/MS[5]	Up: HSP27<br>Down: GAPDH, actin, ENO1	15 POAG, 14 PEXG, 15 CT
Koliakos et al., 2008 [13]	Aqueous Humor and Serum	Targeted Analysis	ELISA and Activity assays	Up: PAB (1.1-fold PEXG vs CT in aqueous humor, 1.1-fold PEXG vs CT in serum, 1.2-fold PES vs CT in serum), hydrogen peroxide (2.3-fold PES vs CT in aqueous humor, 2.8-fold PES vs CT in aqueous humor,	20 PEXG, 20 PES, 20 CT

				1.3-fold PEXG vs CT in serum, 1.7-fold PES vs CT in serum).	
				Down: CAT activity (0.8-fold PEXG vs CT in aqueous humor, 0.7-fold PES vs CT in aqueous humor, 0.6-fold PEXG vs CT in serum, 0.5-fold PES vs CT in serum).	
R. Yagci et al., 2008 [14]	Aqueous Humor and Serum	Targeted Analysis	Spectro-photometric (enzymatic)	Up: Protein carbonyl (1.7-fold in aqueous humor; 1.1-fold in serum)	29 PEXG, 27 CT
Grus et al. 2008 [15]	Aqueous Humor	Untargeted (discovery) and Targeted proteomics (verification)	SELDI-TOF-MS, 2D-electrophoresis, LC-MS/MS &ELISA	Up: TTR (1.9-fold)	52 POAG, 55 CT
Mokbel et al., 2010 [16]	Aqueous Humor and Plasma	Targeted-Proteomics	ELISA	Up: sCD44 (1.8-fold in aqueous humor), EPO (1,8-fold in aqueous humor)	39 POAG, 25 CT
Izzotti et al., 2010 [17]	Aqueous Humor	Targeted Proteomics	Antibody microarray	Up: ATPase, Na^+/K^+ transporting beta 3 polypeptide (ATP1B3, 2.8-fold), GS (2.6-fold), A kinase anchor protein 1 (AKAP1, 2.1- fold), calcium-binding mitochondrial carrier protein Aralar1 (Aralar 1, 2.1-fold), HSP60 (2.2-fold), Translocase of inner mitochondrial membrane 23 (TIM23, 2.1-fold), Cytochrome c (2.2-fold), BCL2-associated X protein (BAX, 2.2-fold), BCL2-interacting killer (BIK, 2.3-fold), Caspase 8 (2.2-fold), Caspase 9 (2.0-fold), TNF receptor associated factor 2 (TRAF2, 2.1-fold), Fas (TNFRSF6)-associated via death domain (FADD, 2.3-fold), Cadherin 3 (2.1-fold), Cadherin 5 (2.1-fold), Calnexin (2.1-fold), Catenin alpha (2.1-fold), Junction plakoglobin (JUP, 2.1-fold), Ankyrin-2 (ANK2, 2.3-fold), Chondroitin sulfate proteoglycan 5 (CSPG5, 2.3-fold), Optineurin (OPTN, 2.0-fold), Neural precursor cell expressed, developmentally down-regulated 4 (NEDD4, 2.2. fold), Protein kinase C, epsilon (PKCε, 2.0-fold), Protein kinase C delta (PKCδ, 2.0-fold), Protein kinase cAMP-dependent catalytic alpha (PRKACA, 2.1-fold), Protein kinase C theta (PKC-θ, 2.1-fold), (NOS2, 2.0-fold), Dynein cytoplasmic light polypeptide 1 (DYNLL1, 2.2-fold)	10 POAG, 14 CT
				Down: SOD1/2 (0.4-fold), Microsomal glutathione S transferase 1 (MGST1, 0.4-fold)	
Duan et al., 2010 [18]	Aqueous Humor	Untargeted Proteomics	2D-electrophoresis & LC-MS/MS	Up: PTGDS (13.1-fold), caspase 14 precursor (5.9-fold), TTR (2.2-fold), ALB precursor (6.0-fold), CysC (5.2-fold), ALB (11.1-fold), TF (5.1-fold).	5 POAG, 5 CT
Ghanem et al., 2010 [19]	Aqueous Humor	Targeted Analysis	Spectro-photometric (enzymatic)	Up activity: GPx (2.9-fold), SOD (1.8-fold), MDA (8-fold)	30 POAG, 25 CT
Bai et al., 2011 [20]	Aqueous Humor	Targeted Proteomics	Quantitative WB	Up: α2M (3.5-fold)	12 glaucoma, 9 CT
Ghanem et al., 2011 [21]	Aqueous Humor	Targeted Proteomics	ELISA	Up: CTGF (3.1-fold PEXG vs CT, 1.6-fold PEXG vs POAG), TIMP-2 (4.8-fold PEXG vs CT, 2.1-fold PEXG vs POAG) MMP-2 (2.8-fold fold PEXG vs CT, 1.5-fold PEXG vs POAG)	30 POAG, 30 PEXG, 25 CT
Balaiya et al., 2011	Aqueous Humor	Targeted Proteomics	Singleplex bead im-	Up: TNF-α (1.7-fold)	32 POAG, 32

[22]			munoassay		CT
Bouhenni et al., 2011 [23]	Aqueous Humor	Untargeted Proteomics	LC-MS/MS	Up: APOA4 precursor (8.6-fold), ALB precursor (unknwon-fold), SERPINC1 precursor (unknwon-fold)<br>Down: TTR precursor (unknwon-fold), PTGDS (0.1-fold), OPT (0.2-fold), IRBP (0.1-fold)	7 PCG, 4 CT
Browne et al. 2011 [24]	Aqueous Humor	Targeted-Proteomics	ELISA	Up: CTGF (2.0-fold PEXG vs CT, 1.9-fold PEXG vs PES, 1.7-fold PEXG vs POAG)	20 POAG, 18 PEXG, 15 PES, 21 CT
Takai et al., 2012 [25]	Aqueous Humor	Targeted Proteomics	Multiplex Immunoassays	Up: IL-8 (2.3-fold POAG vs CT, 4.0-fold PEXG vs CT), TGFβ1 (5.0-fold POAG vs CT, 12.5 PEXG vs CT), SAA (11.9-fold POAG vs CT, 18.3-fold PEXG vs CT).<br>Down: IL-6 (0.2-fold POAG vs CT)	20 POAG, 23 PEXG, 21 CT
Bagnis et al., 2012 [26]	Aqueous Humor	Targeted Proteomics	Antibody microarray	Up: GS (2.0-fold), NOS (2.0-fold)<br>Down: SOD (0.4-fold), GST (0.3-fold)	10 POAG, 10 CT
Saccà et al., 2012 [27]	Aqueous Humor	Targeted Proteomics	Antibody microarray	Up: Apolipoprotein B (APOB, 2.4-fold), APOE (2.1-fold), myotrophin (MTPN, 2,0-fold), myoblast determination protein 1 (MYOD1, 2.0-fold), myogenin (MYOG, 2.0-fold), Vasodilator-stimulated phosphoprotein (VASP, 2.2-fold), HSP60 (2.2-fold), HSP90 (2.0-fold), ubiquitin fusion degradation 1-like (UFD1L, 2.4-fold), Phospholipase C beta (PLCB, 2.5-fold), Phospholipase C gamma (PLCG, 2.1-fold), ANK2 (2.2-fold)	14 POAG, 11 CT
Inoue et al., 2013 [28]	Aqueous Humor	Targeted Proteomics	Multiplex Immunoassays	Up: APOA1 (3.5-fold POAG vs CT, 2.9-fold PEXG vs CT), APOC3 (6.3-fold POAG vs CT, 6.5 PEXG, vs CT), APOE (3.6-fold POAG vs CT, 3.4-fold PEXG vs CT), TTR (2.1-fold POAG vs CT, 2.3-fold PEXG vs CT), α2M (7.0-fold POAG vs CT, 7.5-fold PEXG vs CT)	20 POAG, 32 PEXG, 38 CT
Goyal et al., 2014 [29]	Aqueous Humor	Targeted Analysis	Spectrophotometric (enzymatic or biochemical)	Up activity: SOD (2.1-fold POAG vs CT, 2.0-fold PACG vs CT), GPx (2.5-fold POAG vs CT, 2.3-fold PACG vs CT)<br>Down: Vitamin C (0.6-fold POAG vs CT, 0.6-fold PACG vs CT), vitamin E (0.7-fold POAG vs CT, 0.7-fold PACG vs CT)	30 POAG, 30 PACG, 30 CT
Doudevski et al., 2014 [30]	Aqueous Humor	Targeted Proteomics	ELISA	Up: CLU (1.8-fold), VTN (3.0-fold), C3a (1.7-fold), C5b-9 (4.1-fold).	68 PEXG, 107 CT
Ahoor et al., 2016 [31]	Aqueous Humor and Serum	Targeted Analysis	ELISA	Up: ET-1 (1.2-fold PEXG vs CT and 1.1-fold PES vs CT in aqueous humor; 1.4-fold PEXG vs CT and 1.4-fold PES vs CT in serum)<br>Down: Klotho (0.6-fold PEXG vs CT and 0.9-fold PES vs CT in aqueous humor; 0.8-fold PEXG vs CT and 0.9-fold PES vs CT in serum)	15 PEXG, 15 PES, 15 CT
Kliuchnikova et al., 2016 [32]	Aqueous Humor	Untargeted Proteomics	LC-MS/MS & Label-free protein quantification	Down: APOD (PES vs CT)	7 POAG, 6 PEXG, 5 Pseudoexfoliative cataract, 11 CT
Kaeslin et al., 2016 [33]	Aqueous Humor	Shot-gun Proteomics	LC-MS/MS	Up: Left-right determination factor 1 (LFTY1, 7.0-fold), V-set and immunoglobulin domain-containing protein 4 (VSIG4, 6.2-fold), alpha-1-microglobulin/bikunin precursor (AMBP, 5.0-fold), corticosteroid-binding globulin (CBG, 4.2-fold), matrix Gla protein (MGP, 3.7-fold), scavenger receptor cysteine-rich type 1 protein M130	5 POAG, 5 CT

(CD163, 3.5-fold), MMP2 (3.2-fold), APOC1 (3.2-fold), C1q subunit B (C1QB, 3.2-fold), FGF-binding protein 2 (FGFP2, 3.2 fold), chitotriosidase-1 (CHIT1, 3.2-fold), CD14 (3.2-fold), CO9 (3.1-fold), beta-2-microglobulin (B2MG, 3.1-fold), inter-alpha-trypsin inhibitor heavy chain H2 (ITIH2, 3.0-fold), neutrophil gelatinase-associated lipocalin (NGAL, 2.9.fold), APOD (2.9-fold), keratin type I cytoskeletal 16 (K1C16, 2.9-fold), ITIH4 (2.8-fold), dedicator of cytokinesis protein 10 (DOC10, 2.8-fold), inter-alpha-trypsin inhibitor heavy chain H1 (ITIH1, 2.7-fold), angiotensinogen (ANGT, 2.6-fold), alpha-1-antichymotrypsin (AACT, 2.3-fold), retbindin (RTBDN, 2.2-fold), vitamin D-binding protein (VTDB, 2.2-fold), alpha-2-HS-glycoprotein (FETUA, 2.2-fold), prothrombin (THRB, 2.2-fold), celsolin (GELS, 2.2-fold), APOA4 (2.2-fold), VTN (2.2-fold), IgGFc-binding protein (FCGBP, 2.1-fold), N-acetylmuramoyl-L-alanine amidase (PGRP2, 2.1-fold), thyroxine-binding globulin (THBG, 2.0-fold), CO8B (2.0-fold), alpha-2-antiplasmin (A2AP, 2.0-fold).
Down: N-acetylglucosamine-6-sulfatase (GNS, 0.5-fold), neuronal cell adhesion molecule (NRCAM, 0.5-fold), secretogranin-3 (SCG3, 0.4-fold), semaphorin-4B (SEM4B, 0.4-fold), coagulation factor V (FA5, 0.4-fold), TIMP2 (0.4-fold), peroxiredoxin-2 (PRDX2, 0.4-fold), Ig gamma-3 chain C region (IGHG3, 0.4-fold), amyloid-like protein 2 (APLP2, 0.4-fold), beta-actin-like protein 2 (ACTBL, 0.4-fold), keratin type II cytoskeletal 5 (K2C5, 0.4-fold), carbonic anhydrase 1 (CAH1, 0.4-fold), interphotoreceptor matrix proteoglycan 2 (IMPG2, 0.4-fold), retinal dehydrogenase 1 (AL1A1, 0.4-fold), ankyrin-1 (ANK1, 0.4-fold), tripeptidyl-peptidase 1 (TPP1, 0.4-fold), testican-2 (TICN2, 0.4-fold), testican-1 & testican-3 (TICN1, TICN3, 0.4-fold), fatty acid-binding protein epidermal (FABP5, 0.3-fold), SODC (0.3-fold), pleiotrophin (PTN, 0.3-fold), immunoglobulin lambda-like polypeptide 5 & Ig lambda-1 chain C regions (IGLL5, LAC1, 0.3-fold), fructose-bisphosphate aldolase A (ALDOA, 0.3-fold), peroxiredoxin-6 (PRDX6, 0.3-fold), arginase-1 (ARGI1, 0.3-fold), hemoglobin subunit gamma-1 & hemoglobin subunit gamma-2 (HBG1, HBG2, 0.3-fold), Ig heavy chain V-III region BRO & Ig heavy chain V-III region TEI (HV305, HV316, 0.3-fold), CAT (0.3-fold), ubiquitin-60S ribosomal protein L40 & ubiquitin-40S ribosomal protein S27a & polyubiquitin-B & polyubiquitin-C (RL40, RS27A, UBB, UBC, 0.3-fold), alpha-crystallin A chain (CRYAA, 0.2-fold), ENO1, 0.2-fold), haptoglobin-related protein & haptoglobin (HPTR, HPT, 0.2-fold), heat shock 70 kDa protein 1-like & heat shock 70 kDa protein 1A/1B & heat shock-related 70 kDa protein 2 & heat shock 70 kDa protein 6 & putative heat shock 70 kDa protein 7& heat shock cognate 71 kDa protein (HS71L, HSP71, HSP72, HSP76, HSP77, HSP7C, 0.2-fold), neurotrimin (NTRI, 0.2-fold), pyruvate kinase isozymes M1/M2 & pyruvate kinase isozymes R/L (KPYM, KPYR, 0.2-fold), peptidyl-glycine alpha-amidating monooxygenase (AMD, 0.2-fold), neuro-

				serpin (NEUS, 0.2.fold), Triosephosphate isomerase (TRIS, 0.2-fold), macrophage migration inhibitory factor (MIF, 0.2-fold), aldehyde dehydrogenase, dimeric NADP-preferring (AL3A1, 0.2-fold), phosphatidylethanolamine-binding protein 1 (PEBP1, 0.2-fold), band 3 anion transport protein (B3AT, 0.2-fold), glutathione S-transferase P (GSTP1, 0.2-fold), L-lactate dehydrogenase A chain (LDHA, 0.1-fold), beta-crystallin S (CRBS, 0.1-fold), alpha-enolase & beta-enolase & gamma-enolase (ENOA, ENOB, ENOG, 0.1-fold), phosphoglycerate kinase 1 (PGK1, 0.1-fold), transketolase (TKT, 0.1-fold), heat shock protein beta-1 (HSPB1, 0.1-fold), actin, aortic smooth muscle & actin, cytoplasmic 1 & actin, alpha cardiac muscle 1 & actin, cytoplasmic 2 & actin, gamma-enteric smooth muscle & actin, alpha skeletal muscle & POTE ankyrin domain family member E & POTE ankyrin domain family member F & POTE ankyrin domain family member I (ACTA, ACTB, ACTC, ACTG, ACTH, ACTS, POTEE, POTEF, POTEI, 0.1-fold), TGFβ2 (0.1-fold), protein CREG1 (CREG1, 0.1-fold), peroxiredoxin-1 (PRDX1, 0.1-fold), villin-like protein (VILL, 0.1-fold), aldehyde dehydrogenase, dimeric NADP-preferring & fatty aldehyde dehydrogenase & aldehyde dehydrogenase family 3 member B1 & aldehyde dehydrogenase family 3 member B2 (AL3A1, AL3A2, AL3B1, AL3B2, 0.03-fold).	
Baumane et al., 2017 [34]	Aqueous Humor and Plasma	Targeted Analysis	ELISA	Up: NT-proANP (1.5-fold in plasma, 5.2-fold in aqueous humor)	58 POAG, 32 CT
Hondur et al., 2017 [35]	Aqueous Humor and Blood	Targeted Proteomics	ELISA	Up: Protein carbonyls (2.6-fold in aqueous humor, 1.9-fold in serum), AGEs (3.1-fold in aqueous humor, 1.9-fold in serum)	96 POAG, 64 CT
Garweg et al., 2017 [36]	Aqueous Humor	Targeted Proteomics	Multiplex Immunoassays	Up: CXCL13, CCL24, IL-16, IL-4, CCL13, CCL22, CCL15, CXCL16 (PEXG vs CTs, PES vs CTs, PESL vs CTs)	30 PEXG, 33 PES, 10 PESL, 20 CT
				Down: CCL25	
Ban et al., 2017[37]	Aqueous Humor	Targeted Proteomics	ELISA	Up: Growth differentiation factor 15 (GDF15, 31.7-fold POAG vs CT)	57 POAG, 23 CT
Zhang et al., 2017 [38]	Aqueous Humor	Targeted Proteomics	Multiplex Immunoassays	Up: CD (1.2-fold), sNCAM (1.3-fold), sVCAM-1 (1.9-fold)	34 POAG, 24 CT
Sharma et al., 2018 [39]	Aqueous Humor	Untargeted Proteomics	LC-MS/MS	Up: IGKC (13.6-fold), ITIH4 (4.1-fold), APOC3 (3.4-fold), IDH3A (3.1-fold), cDNA FLJ42083 fis clone TCERX2000613 (LOC105369216, 3.0-fold), Serine/cysteine proteinase inhibitor clade F (SERPINF2, 2.9-fold), Niemann-Pick disease type C2 (NPC2, 2.9-fold), Succinate-CoA ligase subunit β (SUCLG2, 2.7-fold), KIAA0100 (2.3-fold), CCR4-NOT transcription complex subunit 4 (CNOT4, 2.2-fold), aquaporin 4 (AQP4, 2.1-fold), collagen, type XVIII, α 1 (COL18A1, 2.1-fold), NACHT and WD repeat domain containing 1 (NWD1,2.1-fold), transmembrane protein 120B (TMEM120B, 2.1-fold), leucine rich repeat containing 34 (LRRC34, 2.0-fold), FCGBP (1.9-fold), Na/K Transporting ATPase Interacting 2(NKAIN2, 1.9-fold), solute carrier family 35 member C2 (SLC35C2, 1.9-fold), tetraspanin 14	15 POAG, 32 CT

				(TSPAN14, 1.9-fold), family with sequence similarity 171, member B (FAM171B, 1.9-fold), fetuin-B (FETUB, 1.9-fold), LDL receptor-related protein-5 (LRP5, 1.8-fold), fibrinogen b chain FGB (D6REL8, 1.8-fold), hydroxysteroid (1.7-fold), dehydrogenase 10 (HSD17B10, 1.8-fold), phosphatidylinositol glycan class C (PIGC, 1.8-fold), protein cornichon homolog 1 (CNIH1, 1.7-fold), Torsin family 3, member A (TOR3A, 1.7-fold), mediator complex subunit (MED23, 1.7-fold), succinate dehydrogenase assembly factor 2 (SDHAF2, 1.7-fold), protein COMMD3-BMI1 (COMMD3-BMI1, 1.6-fold), inositol polyphosphate 1-phosphatase (INPP1, 1.6-fold), cDNA FLJ57526 (1.5-fold), theumatoid factor D5 light chain (V-kappa-3, 1.5-fold).	
Adav et al., 2018 [40]	Aqueous Humor	Untargeted Proteomics (discovery) & Targeted Proteomics (validation)	SDS-PAGE & LC-MS/MS and MRM-LC-MS/MS	Up: APOA4 (unknown-fold), complement C1s subcomponent (C1s, 1.6-fold), CO9 (2.1-fold), CFI (1.9-fold), C-type lectin domain family 3 (CLEC3A, 1.3-fold), CLU Isoform 4 (1.7-fold), galectin-3-binding protein (GAL3bp 4.9-fold).<br>Down: C1QB (0.6-fold), C1q subunit C (C1QC, 0.4-fold), C3 (0.7-fold), complement C4-A (C4A, 0.7-fold), complement C4-B (C4B, 0.7-fold), complement C5 (C5, 0.3-fold), C8B (0.2-fold), complement component C6 (C6 0.5-fold), complement component C8 alpha chain (C8A, 0.7-fold), complement component C8 gamma chain (C8G, 0.3-fold), complement factor H-related protein 1 (CFHR1, 0.4-fold), isoform of complement factor H-related protein 2 (CFHR2, 0.2-fold), VTN (0.9-fold)	10 POAG, 5 CT
Wang et al., 2018 [41]	Aqueous Humor	Targeted Proteomics	Multiplex Immunoassays	Up: SPARC (1.3-fold), THBS2 (1.6-fold), OPN (1.2-fold)	41 PACG, 22 CT
Kaur et al., 2019 [42]	Aqueous Humor	Untargeted Proteomics	LC-MS/MS	Up: CD14, CD59, CFD, RIRREL3, APOA4, CHGA, MYB, TIMP1, CFD, CD59, MFAP4, AGRN, APOC3	9 POAG, 9 PACG, 9 CT
Nikhalashree et al., 2019 [43]	Aqueous Humor	Untargeted Proteomics	LC-MS/MS	Up: OPN (unknown-fold, POAG vs GT and PACG vs CT), CD (unknown-fold, POAG vs CT), CysC (unknown-fold, POAG vs CT, PACG vs CT)	90 POAG, 72 PACG, 78 CT
Guo et al., 2019 [44]	Aqueous Humor	Targeted Proteomics	ELISA	Up: TGFβ2 (1.3-fold in POAG vs CT)	25 POAG, 21 CACG, 9 PACS, 45 AACG, 26 CT
Can Demirdöğen et al., 2019 [45]	Aqueous Humor and Tear	Targeted Proteomics	ELISA	Up: CTGF (1.6-fold PEXG vs CT, 1.5-fold PES vs CT, in tear)	Tear: 78 PEXG, 77 PES, 78 CT. Aqueous Humor: 8 PEXG, 17 PES, 23 CTs
ten Berge et al., 2019 [46]	Aqueous Humor	Targeted Proteomics	Multiplex Immunoassays	Up: IL-8 (1.5-fold POAG vs CT, 1.5-fold AMD vs CT)	28 glaucoma(22 POAG, 1 NTG, 4 NAG, 1 SGPDS), 12 AMD, 25 RP, 22 CT

Basu et al., 2019 [47]	Aqueous Humor and Trabecular Meshwork	Targeted Proteomics	ELISA (Aqueous Humor), SDS-PAGE & MALDI-TOF (Trabecular Meshwork)	Down: FBLN-7 (0.4-fold PACG vs CTs, 0.3-fold PACG vs POAG, in aqueous Humor)	Aqueous Humor: 8 POAG, 8 PACG, 8 CT. Trabecular Meshwork: 3 POAG, 3 PACG, 3 CT.
Can Demirdöğen, et al. 2020 [48]	Aqueous Humor and Tear	Targeted Proteomics	ELISA	Up: CLU (2.0-fold PEXG vs CT, 2.4 PEXG vs PES, in aqueous humor)	Tear: 80 PEXG, 80 PES, 80 CT. Aqueous Humor: 12 PEXG, 22 OES, 22 CT
Sun et al., 2020 [49]	Aqueous Humor	Targeted Proteomics	ELISA	Up: VEGF-A (1.4-fold Stable NVG vs CT, 1.2-fold Stable-NVG vs CRVO, 1.1-fold Stable-NVG vs NPDR, 1.2-fold Stable-NVG vs BRVO), IL-8 (1.4-fold Stable-NVG vs CT, 1.1-fold Stable-NVG vs CRVO), EPO (1.3-fold Stable-NVG vs CT, 1.2-fold Stable-NVG vs BRVO)	12 NVG, 26 Stable-NVG, 11 CRVO, 18 PACG, 25 PDR, 7 BRVO, 22 CT
Sun et al., 2020 [50]	Aqueous Humor and Vitreous Body	Targeted Proteomics	ELISA	Up: VEGF-A (1.2-fold NVG vs PDR in aqueous humor)	15 NVG, 17 PDR
Ishikawa et al., 2020 [51]	Aqueous Humor	Targeted Proteomics	ELISA	Up: PN (38.5-foldNVG vs PDR), TNC (4.0-fold NVG vs PDR)	11 POAG, 8 NVG secondary to PDR, 9 PDR,4 CT
Hubens et al., 2020 [52]	Aqueous Humor	Untargeted Proteomics	LC-MS/MS	Up: Carboxypeptidase B2 (CPB2), target of Nesh-SH3 (ABI3BP), TIMP2, C1QB, alpha-1B-glycoprotein (A1BG), alpha-1-acid glycoprotein 2 (ORM2), CFI, AGT, Ig kappa chain V-III region POM (IGKV3D-15), tripeptidyl-peptidase 1 (TPP1), alpha-1-antichymotrypsin (SERPINA3), haptoglobin-related protein (HPR), collagen alpha-2(IX) chain (COL9A2), alpha-1-acid glycoprotein 1 (ORM1), triosephosphate isomerase (TPI1), lumican (LUM), SPARC-like protein 1 (SPARCL1), CO9, vitamin D-binding protein (GC), SERPINC1, B2M, histidine-rich glycoprotein (HRG), alpha-2-HS-glycoprotein (AHSG), Ig kappa chain V-III region VG (IGKV3D-11), aldehyde dehydrogenase dimeric NADP-preferring (ALDH3A1), hemoglobin subunit delta (HBD), CysC, VTN, ALB, C8A	POAG with GFS (glaucoma filtration surgery) vs CT
				Down: FN1, ATP synthase subunit alpha mitochondrial (ATP5F1 A), calpain-1 (CAPN10), keratin type II cytoskeletal 2 epidermal (KRT2), fibrinogen alpha chain (FGA), C4b-binding protein alpha chain (C4BPA), vitamin K-dependent protein S (PROS1), keratin type I cytoskeletal 1 (KRT10), CFH, C7, ACTB, Ig heavy chain V–I region HG3 (IGHV1-3), fibrinogen gamma chain (FGG), APLP2, calsyntenin-1 (CLSTN1), C6, zinc-alpha-2-glycoprotein (AZGP1), C5, HPT, Ig kappa chain V-II region FR (IGKV2D-28),	

				1-phosphatidylinositol 3-phosphate 5-kinase (IGKV2D-28), 1-phosphatidylinositol 3-phosphate 5-kinase (PIKFYVE), Wnt inhibitory factor 1 (WIF1), keratinocyte proline-rich protein (KPRP).	
				Up: C1QB, APOC3, A1BG,S ERPINF2, SERPINA3, APOA4), serum amyloid A-4 protein (SAA4), SERPINC1, leucine-rich alpha-2-glycoprotein (LRG1), N-acetylmuramoyl-L-alanine amidase (PGLYRP2), plasminogen (PLG), IgGFc-binding protein (FCGBP), ITIH1, plasma protease C1 inhibitor (SERPING1), ITIH4, AZGP1, keratin type II cytoskeletal 1 (KRT1), APOC1, extracellular matrix protein 1 (ECM1), fetuin-B (FETUB), heat shock 70 kDa protein 1A/1 B (HSPA1A), insulin-like growth factor-binding protein 2 (IGFBP2), neuroblastoma suppressor of tumorigenicity 1 (NBL1), NTRI, reelin (RELN), sex hormone-binding globulin (SHBG), IGKC, Ig gamma-4 chain C region (IGHG4), Ig mu chain C region (IGHM).	POAG with cataracts vs CT
				Down: APLP2, FGG, WIF1, KRT5, alpha-2-macroglobulin (A2M), CLSTN1, C1S, ectonucleotide pyrophosphatase/phosphodiesterase family member 2 (ENPP2), proactivator polypeptide (PSAP), insulin-like growth factor-binding protein 6 (IGFBP6), hemoglobin subunit beta (HBB), FBLN1, NEUS, CFHR1, Dickkopf-related protein 3 (DKK3), LCN1, Fibrillin-1 (FBN1), galectin-3-binding protein (LGALS3BP), beta-crystallin S (CRYGS), SPARCL1, ALDH3A1, triosephosphate isomerase (TPI1), tripeptidyl-peptidase 1 (TPP1), collagen alpha-2(IX) chain (COL9A2), HPR, cathepsin L1 (CTSL), CA1, cell adhesion molecule 1 (CADM1), cadherin-2 (CDH2), di-N-acetylchitobiase (CTBS), protein FAM3C (FAM3C), N-acetylglucosamine-6-sulfatase (GNS), Ig lambda constant 7 (IGLC7), Immunoglobulin lambda-like polypeptide 5 (IGLL5), Ig kappa variable 1D-33 (IGKV1D-33), Ig kappa chain V-III region Ti (IGKV3-20), Ig gamma-1 chain C region (IGHG1), Ig kappa chain V-III region VG (IGKV3D-11), Ig kappa variable 2D-28 (IGKV2D-28), Ig heavy variable 4-34 (IGHV4-34), Ig lambda chain V-III region LOI (IGLV3-21), interphotoreceptor matrix proteoglycan 2 (IMPG2), inter-alpha-trypsin inhibitor heavy chain H5 (ITIH5), vesicular integral-membrane protein VIP36 (LMAN2), low-density lipoprotein receptor-related protein 2 (LRP2), limbic system-associated membrane protein (LSAMP), microfibril-associated glycoprotein 4 (MFAP4), macrophage migration inhibitory factor (MIF), MMP2, out at first protein homolog (OAF), pyruvate kinase isozymes M1/M2 (PKM), peptidyl-prolyl cis-trans isomerase A (PPIA), secretogranin-3 (SCG3), neuroendocrine protein 7B2 (SCG5), SOD1, TICN1, neurosecretory protein VGF (VGF).	
		Targeted Proteomics	LC-MS/MS	Up: ALB, APOC3, CysC, TIMP2, A2M, PGTDS, ENPP2	POAG vs CT
				Down: SOD1	
Lin et al., 2020 [53]	Aqueous Humor	Targeted Proteomics	ELISA	Up: GDF15 (unknown-fold, POAG vs CT, PEXG vs CT)	6 POAG, 6 PEXG
Bur-	Aqueous Humor	Targeted	Multiplex	Up in aqueous humor: IL-5 (2.2-fold), IL-12 (2.0-fold),	27 POAG, 29

gos-Blasco et al., 2020 [54]	mor and Tear	Proteomics	Immunoassays	IL-15 (1.4-fold), IFN-γ (1.7-fold), MIP-1α (3.9-fold). Up in tear: IL-4 (3.4-fold), IL-12 (1.6-fold), IL-15 (2.6-fold), eotaxin, FGF-β (1.8-fold), VEGF (2.3-fold). Down in tear: MIP-1α (0.8-fold)	CT
Nezu et al., 2021 [55]	Aqueous Humor	Targeted Proteomics	Cytometric Immunoassays	Up: MCP-1,IL-6, angiogenin (for predicting POAG)	42 Acute retinal necrosis, 22 Endophthalmitis, 24 Virus-induced anterior uveitis, 20 Sarcoidosis, 33 Uveitis of unknown etiology, 30 Vitreoretinal lymphoma, 26 Uveal melanoma, 52 Rhegmatogenous retinal detachment, 34 PDRy, 21 RVO, 18 RP, 27 Macular hole, 35 Epiretinal membrane, 37 AMD, 41 POAG, 20 atopic dermatitis, 30 CT (cataracts)
Igarashi et al., 2021 [56]	Aqueous Humor	Targeted Proteomics	Immuno-enzymatic assay and multiplex immunoassay	Up in aqueous humor: ATX (POAG vs CT, SOAG vs CT, PEXG vs CT, SOAG vs POAG, PEXG vs SOAG, PEXG vs POAG), TGF-β1 (SOAG vs CT, PEXG vs CT, PEXG vs SOAG, PEXG vs POAG), TGF-β2 (POAG vs CT, SOAG vs CT, POAG vs PEXG, SOAG vs PEXG), TGF-β3 (POAG vs CT, SOAG vs CT, PEXG vs CT, PEXG vs SOAG, PEXG vs POAG, SOAG vs POAG)<br>Down in tear: TGF-β2 (PEXG vs CT)	97 POAG, 48 SOAG, 48 PEXG, 88 CT
Kotikoski, et al., 2002 [57]	Aqueous Humor and Serum	Targeted Quantitative Analysis	Chemiluminescence and RIA	Up: Nitric, nitrate and cGMP without statistical significant differences	38 glaucoma (28 POAG, 6 PEXG and 4 ACG), 38 CT
Galassi et al., 2004 [58]	Aqueous Humor and Serum	Targeted Quantitative Analysis	Spectrophotometry and RIA	Down: Nitrite (0.7-fold in serum, 0.6-fold in aqueous humor), cGMP (0.8-fold in serum, 0.5-fold in aqueous humor)	38 POAG, 46 CT

Bleich et al., 2004 [59]	Aqueous Humor and Plasma	Targeted Metabolomics	ELISA	Up: Hcy (2.0-fold in aqueous humor, 1.3-fold in plasma)	29 PEXG, 31 CT
Castany et al., 2011 [60]	Aqueous Humor	Targeted Metabolomics	HPLC[6]-UV-vis	Up: Ap4A (15-fold)	16 POAG, 16 CT
Nucci et al., 2013 [61]	Aqueous Humor and Blood	Targeted Metabolomics	HPLC-PDA	Up: MDA (2.1-fold in blood, 2.4-fold in aqueous humor)	40 POAG, 26 CT
				Down: ATP-ADP (0.9-fold in blood)	
Buisset et al., 2019 [62]	Aqueous Humor	Targeted Metabolomics	LC-MS/MS	Up: creatinine, carnitine, three short-chain acylcarnitines, glutamine, glycine, alanine, leucine, isoleucine, hydroxyl-proline, and acetyl-ornithine, phosphatidylcholines (PC aa 30:2, 32:1, 34:1, 34:2, 36:2, 36:4, and 38:2), lysophosphatidylcholine, sphingomyelin	26 POAG, 26 CT
				Down: taurine, spermine	
Chen, et al., 2019 [63]	Aqueous Humor	Untargeted Metabolomics	GC/TOF MS	Up: Glycine-2 (8.9-fold PCG vs CT, 3.9-fold PCG vs POAG, 9.0-fold PCG vs ARC), Phenylalanine-1 (1.8-fold PCG vs CT, 1.5-fold PCG vs ARC)	45 PCG, 10 CCs, 10 ARCs, 10 POAG
				Down: Phenylalanine-1 (0.9-fold PCG vs POAG), Urea (0.9-fold PCG vs POAG, 0.6-fold PCG vs CT, 0.8-fold PCG vs ARC)	
Pan et al., 2020 [64]	Aqueous Humor	Untargeted Metabolomics	GC/TOF MS	Up: Pelargonic acid, 2-mercaptoethanesulfonic acid 2, galactose 1, mannose 1, D-erythronolactone 2, dehydroascorbic acid 2, ribitol, D-Talose 1	16 POAG, 24 CT
				Down: Glucose-1-phosphate, methylmalonic acid, spermidine 2, N-cyclohexylformamide 1, sorbitol, biotin	
Barbosa Breda et al., 2020 [65]	Aqueous Humor	Untargeted Metabolomics	1H NMR	Up: Glutamine & glutamate (1.7.fold POAG vs CT, 1.6-fold NTG v CT), glutamine α-ketoglutarate (1.4-fold POAG vs CT, 1.4-fold NTG vs CT), lysine & creatine & phospho-creatine & creatinine α-ketoglutarate (1.5-fold POAG vs CT, 1.5-fold NTG vs CT), glucose & taurine & betaine (1.8-fold POAG vs CT, 1.5-fold NTG vs CT), glucose & Hα of amino acids (1.7-fold POAG vs CT, 1.6-fold NTG vs CT)	30 POAG, 30 NTG, 30 CT
				Down: Valine & β-hydroxybutyrate (0.08-fold POAG vs CT, 0.08-fold NTG vs CT)	

[1]Comparison with other groups is indicated in brackets. [2]CT, control; PESL, pseudoexfoliation syndrome plus luxation (complications); PDR, proliferative diabetic retinopathy; PACS, primary angle-closure suspects; CRVO, central retinal vein occlusion; NPDR, non-proliferative diabetic retinopathy; BRVO, branch retinal vein occlusion; CCs, congenital cataracts; ARCs, aged-related cataracts. [3]Up, up-regulated; [4]Down, down-regulated; [5]MALDI-MS, matrix-assisted laser desorption/ionization tandem mass spectrometry. [6]HPLC, high-performance liquid chromatography

2. Eye tissues and vitreous body

Table S2. Candidate glaucoma biomarkers identified in retina, optic nerve, vitreous body or trabecular meshwork. The study, the followed strategy, the main analytical techniques used, the fold-change of markers when specified, the number of subjects and the disease of recruited patients have been indicated.

Study	Fluid/Tissue	Strategy	Analytical Technique	List of candidate biomarkers (fold-change vs controls)[1]	Samples
Tezel, et al., 2001 [66]	Retina	Targeted Proteomics	Immunohistochemistry	Up[2]: TNF-α, TNFR1 (Not applicable-fold)	14 POAG (20 eyes), 10 CT (20 eyes)
Tezel et al., 2004	Optic Nerve and Retina	Targeted Analysis	Immunostaining	Up: HIF-1α (5.2-fold in optic nerve, 4.7-fold in retina)	28 glaucoma, 20 CT

[67]					
Bhattacharya et al., 2005 [68]	Trabecular Meshwork	Untargeted Proteomics	SDS-PAGE, LC-MS & WB	Up-regulated: Cochlin (unknown-fold)	5 POAG, 35 CT
Bhattacharya et al., 2006 [69]	Optic Nerve	Untargeted Proteomics	LC-MS/MS	Up: PAD2 (not detected in control optic nerves)	8 POAG, 8 CT
Zhang et al., 2008 [70]	Trabecular Meshwork	Untargeted Proteomics	2D-GE, MALDI-TOF-MS/MS & WB	Up: Copine 1 (1.8-fold)	5 POAG, 5 CT
Govindarajan et al., 2008 [71]	Trabecular Meshwork	Targeted Analysis	WB and Spectrophotometric	Up: CAPN10 (unknown fold)	15 POAG, 15 CT
				Down[3]: CAPN10-activity (0.5-fold)	
Tezel et al., 2010 [72]	Retina	Untargeted and Targeted Proteomics	2D-PAGE & LC-MS/MS and WB	Up: Hb (2.0-fold)	38 glaucoma, 30 CT
Tezel et al., 2010 [73]	Retina	Targeted Proteomics	LC-MS/MS	Up: C1s (∞-fold), complement component 1r (C1r, ∞-fold), C1q (∞-fold), C3 (∞-fold), complement component 7 (CO7, 81.2-fold), C8A (∞-fold), C8B (1.1-fold), CO9 (1.4-fold), complement component (3b/4b) receptor-1 (CD35, 1.1-fold), complement component receptor 2 (CR2, 1.2-fold), complement component 5a receptor (C5AR1, ∞-fold), mannan-binding lectin serine protease 1 (MASP1, ∞-fold), mannan-binding lectin serine protease 2 (MASP2, ∞-fold), C-type lectin superfamily member 1 (CLC3A, 2.9-fold), lectin galactoside-binding (Gal_Lectin, 2.6-fold), C1 inhibitor, SERPING 1 (1.6-fold)	10 glaucoma, 10 CT
				Down: Complement factor H (CFH, 0.5-fold), complement component 4b (C4b, 0.9-fold), complement factor H-related 4 (CFHR4, 0.4-fold), complement component 4 binding protein alpha (C4BPA, 0.9-fold), CLU isoform 1 (0.7-fold)	
Yang et al., 2011 [74]	Retina	Targeted Proteomics	LC-MS/MS (label free) & WB	Up: TNF-α (3.1-fold), tumor necrosis factor receptor superfamily member 1A (TNFRSF1A, 2.1-fold), TNFRSF1A-associated death domain-containing protein (TRADD, 2.3-fold), MAP kinase-activating death domain-containing protein isoform a (MADD, 1.9-fold), TNFR-associated factor 1 (TRAF1, 2.4-fold), TNFR-associated factor 2 (TRAF2, 3.6-fold), TNFR-associated factor 4-associated factor 2 (TFAF2, 1.8-fold), TNFR-associated factor 4 isoform 2 (TRAF4 variant, 1.9-fold), TNFR-associated factor 6 (TRAF6, 2.8-fold), TNF-induced protein 1-like adaptor protein (KCTD13, 2.1-fold), nuclear factor-kappa B subunit p105 (NFKB1/p50, 1.9-fold), TNF-induced protein 3-interacting protein 1 (TNIP1, 2.5-fold), inhibitor of kappa-B kinase epsilon (IKKE, 1.9-fold), NF-B essential modulator inhibitor of kappa-B kinase gamma (IKKG, 3.4-fold), receptor (TNFRSF)-interacting serine/threonine kinase 1 (RIPK1, 2.2-fold), rho-associated protein kinase 1 (ROCK1, 2.3-fold), serine/threonine kinase 17a (apoptosis-inducing)	10 glaucoma, 10 CT

				(STK17A, 1.9-fold), mitogen-activated protein kinase 2 (MAP4K2, 6.4-fold), mitogen-activated protein kinase 5 (MAP3K5, 2.2-fold), mitogen-activated protein kinase 14 (MAP3K14, 2.3-fold), mitogen-activated protein kinase 18 (MAP3K18, 2.4-fold), mitogen-activated protein 6 (MAP2K6, 2.8-fold), mitogen-activated protein kinase 1 (variant 1) (MAPK1, 4.4-fold), mitogen-activated protein kinase 3 (MAPK3, 3.0-fold), mitogen-activated protein kinase (MAPK8, 2.9-fold), P21-activated kinase 2 (PAK2, 2.6-fold), nucleotide-binding oligomerization domain-containing protein 1 (NOD1, 2.1-fold), NACHT, LRR and PYD domains-containing protein 3 isoform b (NLRP3, 2.2-fold), caspase 1 isoform alpha (CASP1, 3.1-fold), Bcl2-like 1 (BCL(X)L, 1.9-fold), Bcl2-associated protein (BAX, 4.3-fold), apoptosis-inducing factor 1 (AIFM1, 2.9-fold), endonuclease G (ENDOG, 3.0-fold), caspase 9 (2.1-fold), programmed cell death 10; apoptosis-related protein 15 (PDCD10, 2.7-fold), THAP domain-containing nuclear proapoptotic factor (THAP1, 2.0-fold), prostate apoptosis response protein 4 (PAR4, 2.2-fold), DNA fragmentation factor alpha (DFFA, 2.7-fold), calpain small subunit 1 (CAPNS1, 3.8-fold), calpain 6 (CAPN6, 1.8-fold), CAPN10 (2.0-fold), calpastatin isoform c calpain inhibitor (CAST, 2.3-fold), 78 kDa glucose-regulated protein, heat shock 70kDa protein 5 (GRP78, 5.4-fold), DnaJ (Hsp40) homolog C 10 (DNAJC10, 2.2-fold), endoplasmic reticulum oxidoreductin 1-like beta (ERO1LB, 2.8-fold), protein disulfide isomerase A6 (ERP5) (PDIA6, 3.6-fold), endoplasmic reticulum protein 29 (ERP29, 3.3-fold), serine/threonine-protein kinase/endoribonuclease IRE1 (ERN1, 2.5-fold), activating transcription factor 6 (ATF6, 2,7-fold)	
Mizokami et al., 2011 [75]	Optic Nerve	Targeted Proteomics	Immunohistochemistry	Down-regulated: AQP-9 (POAG vs CT)	1 POAG, 1 CT
Kovacs et al., 2015 [76]	Vitreous Body	Targeted Proteomics	Multiplex Immunoassays	Up: PGF (88.6-fold NVG vs nonDM), VEGF-A (79.5-fold NVG vs nonDM), IL-6 (164.9-fold NVG vs nonDM), IL-8 (30.1-fold NVG vs nonDM).	12 NVG, 29 PDR, 10 DM[4], 29 non-DM
Inafuku et al., 2016 [77]	Vitreous Body	Targeted Proteomics	Glycoblotting (and MALDI-MS) & ELISA	Up: Total N-glycans (1.3-fold NVG vs PDR, 4-fold NVG vs CT), sialylated N-glycans (1.3-fold NVG vs PDR, 4-fold NVG vs CT)	7 NVG (with PDR), 11 PDR, 17 CTs
Funke et al., 2016 [78]	Retina	Untargeted Proteomics	LC-MS/MS	Down-regulated: ANT3(0.4-fold) DFS70 (0.8-fold), MeCp2 (0.6-fold)	5 glaucoma (not defined phenotype), 5 CT
Micera et al., 2016 [79]	Trabecular Meshwork	Targeted Proteomics	Multiplex Immunoassays	Up-regulated: IL-10 (23.8-fold), IL-6 (14.6-fold), IL-5 (13.3-fold), IL-7 (12.5-fold), IL-12p70 (8.7-fold), IL-12p40 (7.7-fold), IL-3 (4.4-fold), IL-21 (3.7-fold), IL-4 (3.7-fold), IL-33 (3.2-fold), TNFα (4.5-fold), IFNγ (2.3-fold), IL-15 (2.2.fold), IL-2 (2.1-fold), IL-1β (1.7-fold), IL-17 (1.6-fold), IL-8 (1.4-fold), IL-34 (1.3-fold), VEGF (6.1-fold), TGFβ1 (6.1-fold), neuro-	40 POAG, 23 CT

				trophin 3 (NT3, 4.7-fold), FGF-β (3.9-fold), nerve growth factor β (NGF-β, 3.8-fold), BDN (3.1-fold) NT4 (2.9-fold), MMP1 (2.0-fold), MMP2 (3.2-fold), TIMP2 (1.8-fold), vascular cell adhesion molecule (VCAM, 7.2-fold), macrophage inflammatory protein 1δ (MIP1δ, 7.0-fold), MIP1α (5.5-fold), Eoxtaxin 1 (2.6-fold), TNF-α converting enzyme (TACE, 2.0-fold), regulated on activation normal T cell expressed and secreted (RANTES, 1.9-fold), intercellular adhesion molecule 2 (ICAM2, 1.9-fold), MIP3β (1.5-fold), neural cell adhesion molecule (NCAM1, 1.4-fold)	
				Down: IL-18 (0.08-fold), IL-16 (0.02-fold), MMP7 (0.5-fold), TIMP4 (0.4-fold), macrophage inflammatory protein 1β (MIP1β, 0.6.fold), ICAM3 (0.1-fold), toll-like receptor 2(TLR2, 0.8-fold)	
Tong et al., 2017 [80]	Vitreous Body	Targeted Proteomics	Multiplex Immunoassays (Cytometric)	Up: IL-2 (3.4-fold AACG vs CT), IL-5 (1.34 AACG vs CT), MCP-1 (5.4-fold AACG vs CT, 1.4-fold POAG vs CT), TNF-α (1.8-fold AACG vs CT), IP-10 (7.0-fold AACG vs CT, 2.4-fold CAGG vs CT, 2.8-fold POAG vs CT)	29 glaucoma (8 AACG, 15 CACG, 6 POAG), 28 CT
Mirzaei et al., 2017 [81]	Vitreous Body and Retina	Targeted Proteomics	Multiplexed Tandem Mass Tag based proteomics (TMT-MS3)	Up: 252 proteins in retina, 554 proteins in vitreous body	10 POAG, 10 CT
				Down: 133 in retina, 559 in vitreous body	
Dreyer et al. 1996 [82]	Vitreous Body	Targeted Metabolomics	HPLC	Up-regulated: Glutamate (2.0-fold)	26 Glaucoma, 21 CT
Doganay et al., 2012 [83]	Vitreous Body	Targeted Metabolomics	Magnetic resonance spectroscopy (MRS)	Up: Glutamate–glutamine/creatine ratio (Glx/Cr, 4.8-fold)	29 POAG, 13 CT

[1]Comparison with other groups is indicated in brackets. [2]Up, up-regulated; [3]Down, down-regulated; [4] DM, diabetes mellitus.

3.Tear film

Table S3. Candidate glaucoma biomarkers identified in tear film. The study, the followed strategy, the main analytical techniques used, the fold-change of markers when specified, the number of subjects and the disease of recruited patients have been indicated.

Study	Fluid/Tissue	Strategy	Analytical Technique	List of candidate biomarkers (fold-change vs controls)[1]	Samples
Ghaffariyeh et al., 2009 [84]	Tear	Targeted Proteomics	ELISA	Up[2]: BDNF (3.2-fold)	20 NTG, 20 CT
Pieragostino et al., 2012 [85]	Tear	Untargeted Proteomics	LC-MS/MS (label free) & SDS-PAGE+MALDI-MS	Altered: LYZ, LCN1, protein S100, Immunoglobulins, PIP, phosphorylated, CST4	Discovery: 4 POAG, 5 PEXG, 4 CTs. Validation: 9 POAG, 7 PEXG, 8 CT
Pieragostino et al., 2013 [86]	Tear	Shotgun Proteomics	nLC-MS/MS	Up: IGHA1 (1.4-fold), IGJ (1.5-fold), IGHA2 (1.6-fold), IGKC (1.6-fold), ALB (1.72-fold), HSPB1 (1.7-fold), CST4 (1.73-fold), PIGR (1.8-fold), PRDX1 (1.8-fold), ACTB (1.9-fold), anti-gamma-actin (ACTG1 (1.9-fold), POTEE/POTEF (2.0-fold), POTEI (2.0-fold), B2M (2.0-fold), POTE ankyrin domain family member J (POTEJ, 2.0-fold), AZGP1 (2.0-fold), TF	9 POAG, 10 CT

				(2.1-fold), PIP (2.4-fold), PRR4 (2.5-fold), LTF (2.6-fold), LYZ (2.7-fold), proline-rich protein 1 (PROL1, 2.9-fold), LCN1 (2.9-fold), zymogen granule protein 16 homolog B (ZG16B, 3.0-fold)	
				Down[3]: IGHG3 (Unknown-fold), Keratin type II cytoskeletal 1 (KRT1, Unknown-fold)	
Gupta et al., 2017 [87]	Tear	Targeted Proteomics	Multiplexed ELISA	Down: IL-12P70 (0.6-fold)	10 POAG, 9 CT
Sahay et al., 2017 [88]	Tear	Targeted Proteomics	Gelatin Zymography	Up: MMP-9 (2.5-fold POAG vs CT, 2.2-fold PACG vs CT, 2.1-fold PES vs CT), MMP-2 (1.1-fold POAG vs CT, 1.1-fold PES vs CT) Down: MMP-2 (0.7-fold PACG vs CT)	27 POAG, 27 PACG, 22 PEXG, 40 PES, 35 CTs
Shpak et al., 2017 [89]	Tear, Aqueous Humor and Serum	Targeted Proteomics	ELISA	Down: CNTF (0.7-fold in Aqueous Humor of POAG vs Cataract, 0.6-fold in Tear of POAG vs Cataract)	55 POAG, 61 Cataracts, 29 CT
Martinez-de-la-Casa et al., 2017 [90]	Tear	Targeted Proteomics	Multiplexed Immunoassay	Up: IL-2, IL-5, IL-10, IL-12 p70, IL-13, IL-15, IL-17, FGF basic, PDGF-BB, TNF-α in POAG (preservative vs CTs)	20 POAG (preservative), 20 POAG (preservative-free), 39 CT
Reddy et al., 2018 [91]	Tear	Targeted Proteomics	Gelatine zymography, ELISA and Multiplex Immunoassay	Up: MMP-9 (7.1-fold POAG vs CT, 5.7-fold NTG vs CT, 1.2-fold POAG vs NTG), MMP-2 (2.6-fold POAG vs CT, 3.3-fold NTG vs CT, 0.8-fold POAG vs NTG), TIMP-1 (1.3-fold POAG vs CT, 1.2-fold POAG vs NTG), GM-CSF (1.5-fold POAG vs NTG), fractalkine (1.2-fold POAG vs NTG), IP-10 (1.8-fold POAG vs NTG), macrophage derived chemokine (MDC, 1.9-fold POAG vs NTG), platelet derived growth factor-AA (PDGF-AA, 3.8-fold POAG vs NTG), IL-1α (1.2-fold POAG vs NTG), interleukin 1 receptor antagonist (IL-1Ra, 1.6-fold POAG vs NTG), IL-8 (1.6-fold POAG vs NTG), CXCL-1 (1.1-fold POAG vs NTG), epidermal growth factor (EGF, 1.2-fold PEXG vs NTG), eotaxin (1.9-fold NTG vs POAG, interferon-α2 (IFN-α2, 1.6-fold NTG vs POAG), IL-7 (1.3-fold NTG vs POAG), MCP-1 (1.3-fold NTG vs POAG), TNF-β (1.3-fold NTG vs POAG)	30 POAG, 30 NTG, 30 CT
				Down: MMP-1 (0.8-fold POAG vs CT, 0.8-fold POAG vs NTG)	
Csősz et al., 2019 [92]	Tear and Aqueous Humor	Targeted Proteomics	Multiplexed Immunoassay	Down: IFN-γ, GMCSF, IL-5 in tear of patients who developed complications after one year	12 POAG, 8 PACG
Sedlak et al., 2020 [93]	Tear	Targeted Analysis	Spectrophotometric (enzymatic and non-enzymatic)	Up: SOD (unknown-fold), CAT (unknown-fold), GPx (unknown-fold), AOPP (1.1 BR+BAC vs CT or T, 1.1-fold T+BAV vs CT or T), Total Oxidant Status (TOS, 1.2-fold BR+BAC vs CT or T), 1.2-fold T+BAC vs CT or T), Oxidative Stress Index (OSI, 1.1-fold BR+BAC vs CT or T, 1.21 T+BAC vs CT or T).	17 glaucoma treated with preservative-free 0.5% timolol (T), 24 glaucoma treated with BAC-preserved 0.5% timolol (T+BAC), 19 glaucoma treated with BAC-preserved

					brimonidine (BR+BAC), 25 CTs
Roedl et al., 2007 [94]	Tear and Plasma	Targeted Metabolomics	HPLC-fluorescence	Up: Hcy (1.8-fold in tear fluid, 1.4-fold in plasma)	30 PEXG, 30 CT
Rossi et al., 2019 [95]	Tear	Targeted Metabolomics & Untargeted Proteomics	Direct Infusion UPLC-MS/MS (DIMS, metabolomics) & LC-MS/MS (label free Proteomics)	Up-proteins: LYZ, JUP, Protein PML<br>Down-proteins: TXN, Actin, ACTG1<br>Down-metabolites: Alanine (0.7-fold), arginine (0.6-fold), glycine\lysine (0.7-fold), leucine\isoleucine\proline-OH (0.6-fold), methionine (0.7-fold), phenylalanine (0.6-fold), proline (0.7-fold), valine (0.7-fold), C2 (0.5-fold), C22:0-LPC (0.5-fold), C24:0-LPC (0.5-fold)	16 POAG, 17 CT

[1]Comparison with other groups is indicated in brackets. [2]Up, up-regulated; [3]Down, down-regulated.

4. Serum/Blood

Table S4. Candidate glaucoma biomarkers identified in blood, serum or plasma (in addition to other fluid). The study, the followed strategy, the main analytical techniques used, the fold-change of markers when specified, the number of subjects and the disease of recruited patients have been indicated.

Study	Fluid/Tissue	Strategy	Analytical Technique	List of candidate biomarkers (fold-change vs controls)[1]	Samples
Weinstein et al., 1996 [96]	Blood (Lymphocytes)	Targeted Proteomics	Immunoblot	Down[2]: 3α- HSD (0.4-fold)	17 POAG, 22 CT
Tezel et al., 1999 [97]	Serum	Targeted Proteomics	WB and ELISA	Up[3]: HS (1.8-fold NTG vs CT, 1.5-fold NTG vs POAG), CS (2.2 NTG vs CT, 1.5-fold NTG vs POAG)	60 NTG, 36 POAG, 20 CT
Zabala et al., 1998 [98]	Red Blood Cell	Targeted Proteomics	Spectrophotometric (Enzymatic activity)	Up: AChE (1.3-fold)	19 POAG, 20 CT
Maruyama et al., 2000 [99]	Serum	Untargeted analysis	WB, 2DGE and LC-ESI-MS	Up: γ-enolase	56 POAG, 23 NTG, 60 CT
Yang et al., 2001 [100]	Serum and peripheral blood mononuclear cells	Targeted Proteomics	ELISA	$CD3^+CD8^+$ lymphocytes (1.9-fold POAG vs CT, 1.4-fold NTG vs CT), $CD8^+$HLA-DR^+ lymphocytes (1.7-fold NTG vs CT), $CD5^+$ lymphocytes (1.4-fold POAG vs CT), sIL-2R (1.8-fold POAG vs CT, 1.7-fold NTG vs CT)	18 POAG, 20 NTG, 18 CT
Yang et al., 2001 [101]	Serum	Untargeted analysis (discovery) and Targeted analysis (validation)	WB, 2DGE and LC-ESI-MS (Discovery) & ELISA (validation)	Up: anti-GST antibody (1.4-fold POAG vs CT, 1.3-fold NTG vs CT)	40 NTG, 25 POAG, 25 CT
Kremmer et al., 2001 [102]	Serum	Targeted Proteomics	ELISA	Up: Phosphatidylserine IgG (2.7-fold NTG vs CT, 2.3-fold NTG vs POAG), phosphatidylserine IgM (1.9-fold NTG vs CT, 2.2-fold NTG vs POAG)	43 NTG, 40 POAG, 40 CT
Lip et al., 2002 [103]	Plasma	Targeted Proteomics	ELISA	Up: VEGF (1.8-fold POAG vs CT, 2.7-fold NTG vs CT, 1.5-fold NTG vs POAG), vWf (2.1-fold POAG vs CT, 2.0-fold NTG vs CT)<br>Down: sFlt-1 (0.2-fold POAG vs CT, 0.6-fold NTG vs CT)	24 POAG, 26 NTG, 26 CT
Wunderlich et al., 2002 [104]	Leukocytes	Targeted metabolomics	WB	Up: 20S proteasome α-subunit (3.4-fold Glaucoma vs CT)	6 HTG, 6 NTG, 6 CT

Golubnitschaja et al., 2004 [105]	Blood (Leukocytes)	Targeted Proteomics	WB	Up: MT1-MMP (Unknown-fold)	6 NTG, 6 CT
Emre et al., 2005 [106]	Plasma	Targeted Proteomics	Radioimmunoassay	Up: ET-1 (1.3-fold)	16 POAG, 15 CT
Gherghel et al., 2005 [107]	Blood	Targeted Proteomics	Spectrophotometric (Enzymatic)	Down: GSH (0.7-fold)	21 POAG, 34 CT
Yildirim et al., 2005 [108]	Blood	Targeted analysis	Spectrophotometric (Analysis of activity)	Up: Plasma MDA (2.3-fold)	40 POAG, 60 CT
Noureddin et al., 2006 [109]	Plasma	Targeted Proteomics	ELISA	Up: Thymulin (3.6-fold)	28 POAG (non-treated), 28 CT
Grus et al., 2006 [110]	Serum	Untargeted analysis (discovery) and Targeted analysis (validation)	WB (discovery) and ELISA (validation)	Up: α-fodrin (1.4-fold NTG vs CT, 1.2-fold NTG vs POAG)	40 POAG, 40 NTG, 40 CT
Acar et al., 2009 [111]	Red Blood Cell	Targeted Proteomics	LC-ESI-MS/MS	Down: PlsC, DHA-PC	31 POAG, 16 CT
Huang et al., 2010 [112]	Serum	Targeted Proteomics	ELISA	Up: IL-4 (1.5-fold), IL-6 (1.5-fold), IL-12p70 (1.4-fold)	32 POAG, 26 CT
				Down: TNF-α (0.9-fold)	
Engin et al., 2010 [113]	Serum	Targeted analysis	Spectrophotometric (Enzymatic) and HPLC-UV	Up: MDA (1.2.fold), serine (1.2-fold), TF (1.1-fold), vitamin A (1.2-fold), vitamin E (1.5-fold)	160 glaucoma (type non-indicated), 31 CT
				Down: TAC (0.9-fold), SOD (0.9-fold), GPx (0.8-fold)	
Sorkhabi et al., 2011 [114]	Serum and Aqueous Humor	Targeted analysis	ELISA and spectrophotometric	Up: 8-OHdG (2.3-fold in aqueous humor, 1.3-fold in serum)	15 POAG, 13 PEXG, 27 CT
				Down: TAS (0.7-fold in aqueous humor, 0.8-fold in serum)	
Chang et al., 2011 [115]	Serum	Targeted analysis	Spectrophotometric	Up: MDA (1.2-fold), conjugated diene (1.1-fold), AOPP (1.1-fold), protein carbonyl (1.2-fold), ischemia-modified ALB (1.05-fold), 8-OHdG (1.1-fold).	50 PACG, 50 CT
Majsterek et al., 2011 [116]	Red Blood Cell	Targeted analysis	Spectrophotometric (Analysis of activity)	Down: CAT (0.6-fold), SOD (0.6-fold), GPX (0.8-fold).	20 POAG, 20 CT
Ghaffariyeh et al., 2011 [117]	Serum	Targeted Proteomics	ELISA	Down-regulated: BDNF (0.7-fold)	25 POAG, 25 CT
Tanito et al., 2012 [118]	Plasma	Targeted analysis	Spectrophotometric	Down: Ferric-reducing activity (0.96-fold POAG vs CT), thiol antioxidant activity (0.9-fold PEXG vs CT, 0.9-fold PEXG vs POAG).	206 POAG, 199 PEXG, 126 CT
Tezel et al., 2012 [119]	Serum	Untargeted Proteomics (discovery) and targeted analysis (validation)	IgG elutes and LC-ESI-MS/MS (discovery) & ELISA (validation)	Up: AIF (4.3-fold), (CREB)-binding protein (4.3-fold), ephrin type-A receptor (7.8-fold), huntingtin (4.1-fold).	111 POAG, 49 CT

Zanon-Moreno et al., 2013 [120]	Plasma	Targeted analysis	LC-UV, LC(RP)-electrochemical detector & Spectrophotometric (activity)	Up: GPx (1.5-fold)	250 POAG, 250 CT
				Down: vitamin E (0.9-fold), vitamin C (1.2-fold)	
Abu-Amero et al., 2013 [121]	Plasma	Targeted analysis	Spectrophotometric (enzymatic)	Down: TAS (0.5-fold)	139 POAG, 148 CT
López-Riquelme et al., 2014 [122]	Plasma	Targeted analysis	ELISA, Chemiluminescence immunoassay, HPLC-UV	Up: ET-1 (1.9-fold POAG vs CT, 1.4-fold NTG vs CT), Hcy (1.3-fold POAG vs CT, 1.1-fold NTG vs CT)	48 POAG, 15 NTG, 75 CT
				Down: Vitamin E (0.7-fold NTG vs CT, 0.7-fold NTG vs POAG)	
González-Iglesias et al., 2014 [123]	Serum	Untargeted Proteomics (discovery) and targeted proteomics (validation)	2D-DIGE, nLC-MS/MS and MALDI-TOF/TOF (discovery) & ELISA (validation)	Up: APOA4 (2.7-fold POAG vs CT, 1.5-fold PEXG vs CT, 1.8-fold POAG vs CT), C3 (1.5-fold POAG vs CT, 1.4 fold POAG vs PEXG), TTR (1.8-fold POAG vs CT, 1.5-fold POAG vs PEXG), TF (1.7-fold POAG vs CT, 1.5-fold POAG vs PEXG), VTN (2.2-fold POAG vs CT, 1.6-fold PEXG vs CT), fibulin-1 (FBLN1, 1.9-fold POAG vs CT, 1.5-fold PEXG vs CT), APOA1, 1.3-fold POAG vs CT), alpha-1 antitrypsin (SERPINA1, 1.5-fold POG vs CT, 1.3-fold POAG vs PEXG), CFH (1.3-fold POAG vs CT), apolipoprotein L1 (APOL1, 1-4-fold POAG vs CT), ficolin-3 (FCN3, 1.3-fold POAG vs CT, 1.3-fold POAG vs PEXG)	Discovery: 53 POAG, 45 PEXG, 41 CT. Validation: 20 POAG, 14 PEXG, 17 CT.
				Down: IGHG2 (0.7-fold POAG vs CT, 0.7-fold PEXG vs CT), C4A (0.8-fold POAG vs CT)	
Ozgonul et al., 2016 [124]	Blood	Targeted Analysis	Spectroscopy (Hematology and Chemistry analyzers)	Up: NLR (1.2-fold POAG vs CT, 1.1-fold OHT vs CT), PLR (1.1-fold POAG vs CT, 1.1-fold OHT vs CT)	84 POAG, 94 OHT, 80 CT
Kurtul et al., 2017 [125]	Serum	Targeted Proteomics	Biochemical analyzer	Up: LDL (1.2-fold PEXG vs CT, 1.1-fold PES vs CT).	20 PEXG, 52 PES, 47 CT
Li et al., 2017 [126]	Plasma	Targeted Proteomics	Immunoturbidimetry	Down: C3 (0.9-fold PACG vs CT, 0.9-fold female PACG vs female CT)	237 PACG, 158 CT
Oddone et al., 2017 [127]	Serum	Targeted Proteomics	ELISA	Down: BDNF (0.8-fold), NGF (0.7-fold)	45 POAG, 15 CT
Li et al., 2017 [128]	Blood	Targeted Analysis	Biochemical analyzer	Up: White blood cell (1.05.fold), neutrophil (1.2-fold), NLR (1.4-fold).	771 PACG, 770 CT
				Down: LMR (0,7-fold)	
Rokicki et al., 2017 [129]	Serum	Targeted Proteomics	Spectrophotometric	Up: Lipofuscin (1.2-fold), MDA (1.5-fold), TOS (2.8-fold).	30 POAG, 25 CT
				Down: Total SOD activity (0.8-fold), mitochondrial SOD (0.8-fold)	
Kondkar et al. 2018 [130]	Plasma	Targeted Proteomics	ELISA	Up: TNF-α (2.0-fold)	51 POAG, 88 CT
Kondkar et al., 2018 [131]	Plasma	Targeted Proteomics	ELISA	Up-regulated: TNF-α (6.0-fold)	49 PEXG, 88 CT
Yaz et al., 2019 [132]	Serum	Targeted analysis	Spectrophotometric	Up: MDA (5.0-fold PEXG vs CT, 2.1-fold PES vs CT, 1.3-fold PEXG vs PES), GSH (1.6-fold PEXG vs CT, 1.6-fold	58 PEXG, 47 PES,

				PES vs CT)	134 CT
				Down: SOD activity (0.3-fold PEXG vs CT, 0.3-fold PES vs CT), CAT activity (0.6-fold PEXG vs CT, 0.5-fold PES vs CT), nitric oxide (0.8-fold PEXG vs CT, 0.7-fold PEXG vs PES)	
Yang et al., 2019 [133]	Blood	Targeted analysis	Flow cytometry and ELISA	Up: $CD4^+$ T cells (3.0-fold), IL-1β (unknown-fold), IFN-γ (unknown-fold), TNF-α (unknown-fold)	32 POAG, 21 CT
				Down: $CD4^+/CD25^+/FoxP3^+$ (unknown-fold), $CD8^+/CD25^+/FoxP3^+$ (unknown-fold)	
Abessolo et al., 2019 [134]	Blood	Targeted Proteomics	Unknown	Up: Neuroglobin (5.2-fold)	64 POAG, 64 CT
Karakurt et al., 2019 [135]	Serum	Targeted analysis	Bioanalyzer and spectrophotometric	Up: Ischemia-modified ALB (1.2-fold), disulfide (1.3-fold), disulfide/native thiol (1.1-fold), disulphide/total thiol (1.1-fold)	70 POAG, 87 CT
				Down: Total thiol (0.8-fold), native thiol (0.8-fold)	
Beutgen et al., 2019 [136]	Serum	Untargeted Proteomics and Targeted Quantitative Proteomics (protein microarrays)	Serological proteome analysis (2D-PAGE, WB, Immunoblot and LC-MS/MS) & Multiplexed Immunoassays	Up: CALD1 (2.0-fold), PGAM1 (1.2-fold), VDAC2 (1.4-fold)	Discovery: 6 POAG, 6 CT. Validation: 60 POAG, 45 CT
Maric et al., 2019 [137]	Serum	Targeted analysis	ELISA	Up: Serum HS (1.2-fold PEXG vs CT, 1.5-fold PEXG vs POAG), CS (1.2-fold PEXG vs CT)	47 PEXG, 43 POAG, 22 PES, 53 CT
Igarashi et al., 2020 [138]	Serum	Targeted Proteomics	ELISA	Down: BDNF (0.6.fold POAG vs CT, 0.5-fold NTG vs CT, 1.3-fold POAG vs NTG)	16 POAG, 11 NTG, 51 CT
Shin et al., 2020 [139]	Serum	Targeted Proteomics	ELISA	Up: MBP (4.7-fold NTG vs CT, 5.7-fold HTG vs CT), anti-SSA (1.5-fold NTG vs HTG), anti-SSB (1,1-fold NTG vs CT, 1.1-fold NTG vs HTG)	17 NTG (OAG), 7 HTG (OAG), 17 CT
				Down-regulated: Anti-α-fodrin antibody (IgG, 0.6-fold NTG vs CT, 0.4-fold NTG vs HTG), Anti-α-fodrin antibody (IgA, 0.6-fold NTG vs HTG)	
Li et al., 2020 [140]	Serum	Targeted analysis	Spectrophotometric (enzymatic)	Up: MDA (5.5-fold PACG vs CT), hydrogen peroxide (2.2-fold PCAG vs CT)	94 PACG, 89 CT
				Down: SOD (0.8.fold PACG vs CT), TAS (0.8-fold PACG vs CT)	
Beutgen et al., 2020 [141]	Serum	Untargeted Proteomics and Targeted Quantitative Proteomics (protein microarrays)	Immunoprecipitation and LC-ESI-MS/MS (Discovery) &Multiplex Microarray Analysis (Validation)	Up: PNMA2 autoantibody (1.2-fold), TARS autoantibody (1.4-fold), C1QBP autoantibody (1.2-fold), HSPD1 autoantibody (1.4-fold)	Discovery: ,30 POAG, 30 CT. Validation: 120 POAG, 120 CT
Mirza et al., 2020 [142]	Blood	Targeted analysis	Spectroscopy (Hematology and Chemistry analyzers)	Up: MHR (1.5-fold PEXG vs CT, 1.5-fold PES vs CT)	21 PEXG, 21 PES, 21 CT
Kondkar	Plasma	Targeted	ELISA	Up: 8-OHdG (1.4-fold)	50 POAG,

et al., 2020 [143]		analysis			45 CT
Gulpamuk et al., 2020 [144]	Serum	Targeted Proteomics	Spectrophotometric (enzymatic)	Up: Ischemia-modified ALB (1.1-fold POAG vs CT)<br>Down: Native thiol (0.9-fold POAG vs CT, 0.9-fold OHT vs CT), total thiol (0.9-fold POAG vs CT, 0.9-fold OHT vs CT)	30 POAG, 30 OHT, 30 CT
Zhang et al., 2021 [145]	Blood	Targeted analysis	Bioanalyzer	Up: White blood cell (1.3-fold NVG-RVO vs CT, 1.2-fold NVG-DR vs CT), neutrophil (1.4-fold NVG-RVO vs CT, 1.3-fold NVG-DR vs CT), NLR (1.3-fold NVG-RVO vs CT, 1.3-fold NVG-DR vs CT).<br>Down: LMR (0.7-fold NVG-RVO vs CT, 0.7-fold NVG-DR vs CT)	38 NVG (secondary to RVO), 46 NVG (secondary to DR), 59 CT
Ren et al., 2006 [146]	Plasma and red blood cells	Targeted metabolomics	GC -MS & Spectrophotometry	Up: Total LCPUFA (1.2-fold in red cell colline phosphoglycerides, 1.1-fold in ethanolamine phosphoglycerides).<br>Down: EPA (0.5-fold in red cell colline phosphoglycerides, 0.6-fold in ethanolamine phosphoglycerides, 0.5-fold in serine phosphoglycerides, 0.4-fold in plasma), DHA (0.8-fold in red cell colline phosphoglycerides, 0.7-fold in plasma), total LCPUFA (0.6-fold in plasma).	10 POAG, 8 CT
Fraenkl et al., 2011 [147]	Plasma and urine	Targeted metabolomics	Ion Chromatography	Down: Citrate (0.8-fold in plasma)	21 glaucoma (12 NTG, 8 POAG, 1 PEXG), 21 CT
Tranchina et al., 2011 [148]	Plasma	Targeted metabolomics	Competitive chemiluminescent enzyme immunoassay	Up: Hcy (1.3-fold PEXG vs CT, 1.2-fold PEXG vs POAG).	36 PEXG, 40 POAG, 40 CT
Javadiyan et al., 2012 [149]	Serum	Targeted metabolomics	LC-MS/MS	Up: ADMA (1.1-fold), SDMA (1.2-fold)	211 POAG, 295 CT
Burgess et al., 2015 [150]	Plasma	Untargeted metabolomics	LC-MS/MS	Up (unknown-fold): Palmitoylcarnitine, monoglyceride (22:5), triglyceride (12:0/18:4/22:6), cholane tetrol C17 sphinganine, C19 sphinganine, C17 sphingosine, heptadecanedione pentadecanone, heptadecanone heptadecanediol, hydroxytetradecanedioic acid, fluorohydroxyD3 (triterpene), DG(24:0/22:6), HODE-cholesteryl ester, hydroxyergocalciferol; DG(24:0/22:6), diapo-zeta-carotene (terpene), dihydroergocalciferol, cholestanediol, azacholecalciferol, decaprenol (terpene), ergostanol, heptadecylbenzenediol, HODE-methyl ester; monoglyceride, ergosterol.<br>Down (unknown-fold): Multiple vitamin D analogs, TG (12:0/16:0/20:5), C19 sphingosine 1-phosphate, MG(24:1), TG (18:0/18:0/20:0), complex glycosphingolipid	72 POAG, 72 CT
Michalczuk et al., 2017 [151]	Plasma and urine	Targeted metabolomics	Enzymatic	Down: Citrate (0.8-fold in plasma, 0.6-fold urine)	34 glaucoma, 34 CT
Rong et al., 2017 [152]	Serum	Targeted metabolomics	GC-MS	Up: PA (1.2-fold), GLA (1.7-fold), ARA (0.8-fold), adrenic acid (1.3-fold)<br>Down: Linoleic acid (0.9-fold)	38 PACG, 48 CT
de la Barca	Plasma	Targeted	FIA-MS/MS &	Up: Methionine sulfoxide (1.3-fold), tyrosine (1.2-fold),	36 POAG,

et al., 2018 [153]		metabolomics	LC-MS/MS	methionine (1.3-fold), propionyl-carnitine C3 (1.3-fold), hexose (1.1-fold), PC aa 34:2 (1.1-fold), PC aa 36:4 (1.1-fold), PC aa 34:4 (1.2-fold), PC ae 40:1 (1.2-fold)	27 CT
				Down: Spermine (0.97-fold), octadecadienyl-carnitine (C18:2, 0.8-fold), spermidine (0.9-fold), octadecenoyl-carnitine (C18:1, 0.8-fold).	
Vohra et al., 2019 [154]	Serum	Targeted metabolomics	HPLC-Fluorescence	Up: Valine (1.2-fold), ornithine (1.2-fold)	12 NTG, 11 CT
				Down: Lactate (0.6-fold), total amino acids (0.9-fold)	
Umeno et al., 2019 [155]	Serum	Targeted metabolomics	LC-MS/MS	Up: HODE (1,3-fold), HETE (1.4-fold)	198 POAG (98 NTG, 114 HTG), 119 CT
Li et al., 2019 [156]	Serum	Targeted metabolomics	Spectrophotometric (enzymatic)	Down: Uric acid (0.8-fold), uric acid/creatinine (0.9-fold)	163 POAG, 103 CT
Lin et al., 2020 [157]	Plasma	Targeted metabolomics	Spectroscopy (Spectrophotometry or LC-Fluorimeter)	Up: Hcy (1.1-fold POAG vs CT, 1.2-fold NTG vs CT), Cys (1.1-fold POAG vs CT, 1.2-foldNTG vs CT)	42 POAG, 20 NTG, 52 OHT, 78 CT
				Down: Hydrogen sulfide (0.8-fold POAG vs CT, 0.8-fold POAG vs OHT)	
Nzoughet et al., 2020 [158]	Plasma	Untargeted metabolomics	LC-HRMS	Up: N-acetyl-L-leucine (1.8-fold), 1-oleoyl-rac-glycerol (1.6-fold), arginine (1.3-fold), rac-glycerol 1-myristate (1.3-fold), cystathionine (1.6-fold)	34 POAG, 30 CT
				Down: Nicotinamide (0.6-fold), hypoxanthine (0.6-fold), 1-methyl-6,7-dihydroxy- 1,2,3,4-tetrahydroisoquinoline (0.5-fold), xanthine (0.7-fold)	

[1]Comparison with other groups is indicated in brackets. [2]Down, down-regulated; [3]Up, up-regulated

References

1. Tripathi, R.C.; Li, J.; Chan, W.F.; Tripathi, B.J. Aqueous humor in glaucomatous eyes contains an increased level of TGF-beta 2. *Exp. Eye Res.* **1994**, *59*, 723–727, doi:10.1006/exer.1994.1158.
2. Tezel, G.; Kass, M.A.; Kolker, A.E.; Becker, B.; Wax, M.B. Plasma and aqueous humor endothelin levels in primary open-angle glaucoma. *J. Glaucoma* **1997**, *6*, 83–89.
3. Vesaluoma, M.; Mertaniemi, P.; Mannonen, S.; Lehto, I.; Uusitalo, R.; Sarna, S.; Tarkkanen, A.; Tervo, T. Cellular and plasma fibronectin in the aqueous humour of primary open-angle glaucoma, exfoliative glaucoma and cataract patients. *Eye* **1998**, *12*, 886–890, doi:10.1038/eye.1998.224.
4. Kee, C.; Son, S.; Ahn, B.H. The relationship between gelatinase A activity in aqueous humor and glaucoma. *J. Glaucoma* **1999**, *8*, 51–55.
5. Ferreira, S.M.; Lerner, S.F.; Brunzini, R.; Evelson, P.A.; Llesuy, S.F. Oxidative stress markers in aqueous humor of glaucoma patients. *Am. J. Ophthalmol.* **2004**, *137*, 62–69, doi:10.1016/s0002-9394(03)00788-8.
6. Navajas, E.V.; Martins, J.R.M.; Melo, L.A.S.; Saraiva, V.S.; Dietrich, C.P.; Nader, H.B.; Belfort, R. Concentration of hyaluronic acid in primary open-angle glaucoma aqueous humor. *Exp. Eye Res.* **2005**, *80*, 853–857, doi:10.1016/j.exer.2004.12.016.
7. Määttä, M.; Tervahartiala, T.; Harju, M.; Airaksinen, J.; Autio-Harmainen, H.; Sorsa, T. Matrix metalloproteinases and their tissue inhibitors in aqueous humor of patients with primary open-angle glaucoma, exfoliation syndrome, and exfoliation glaucoma. *J. Glaucoma* **2005**, *14*, 64–69, doi:10.1097/01.ijg.0000145812.39224.0a.
8. Min, S.H.; Lee, T. Il; Chung, Y.S.; Kim, H.K. Transforming growth factor-beta levels in human aqueous humor of

glaucomatous, diabetic and uveitic eyes. *Korean J. Ophthalmol.* **2006**, *20*, 162–165, doi:10.3341/kjo.2006.20.3.162.

9. Yu, X.-B.; Sun, X.-H.; Dahan, E.; Guo, W.-Y.; Qian, S.-H.; Meng, F.-R.; Song, Y.-L.; Simon, G.J. Ben Increased levels of transforming growth factor-betal and -beta2 in the aqueous humor of patients with neovascular glaucoma. *Ophthalmic surgery, lasers imaging Off. J. Int. Soc. Imaging Eye* **2007**, *38*, 6–14, doi:10.3928/15428877-20070101-01.
10. Nolan, M.J.; Giovingo, M.C.; Miller, A.M.; Wertz, R.D.; Ritch, R.; Liebmann, J.M.; Rand Allingham, R.; Herndon, L.W.; Wax, M.B.; Smolyak, R.; et al. Aqueous humor sCD44 concentration and visual field loss in primary open-angle glaucoma. *J. Glaucoma* **2007**, *16*, 419–429, doi:10.1097/IJG.0b013e318050ab4b.
11. Joachim, S.C.; Bruns, K.; Lackner, K.J.; Pfeiffer, N.; Grus, F.H. Antibodies to α B-crystallin, vimentin, and heat shock protein 70 in aqueous humor of patients with normal tension glaucoma and IgG antibody patterns against retinal antigen in aqueous humor. *Curr. Eye Res.* **2007**, *32*, 501–509, doi:10.1080/02713680701375183.
12. Joachim, S.C.; Wuenschig, D.; Pfeiffer, N.; Grus, F.H. IgG antibody patterns in aqueous humor of patients with primary open angle glaucoma and pseudoexfoliation glaucoma. *Mol. Vis.* **2007**, *13*, 1573–1579.
13. Koliakos, G.G.; Befani, C.D.; Mikropoulos, D.; Ziakas, N.G.; Konstas, A.G.P. Prooxidant-antioxidant balance, peroxide and catalase activity in the aqueous humour and serum of patients with exfoliation syndrome or exfoliative glaucoma. *Graefe's Arch. Clin. Exp. Ophthalmol.* **2008**, *246*, 1477–1483, doi:10.1007/s00417-008-0871-y.
14. Yağci, R.; Ersöz, I.; Erdurmuş, M.; Gürel, A.; Duman, S. Protein carbonyl levels in the aqueous humour and serum of patients with pseudoexfoliation syndrome. *Eye* **2008**, *22*, 128–131, doi:10.1038/sj.eye.6702751.
15. Grus, F.H.; Joachim, S.C.; Sandmann, S.; Thiel, U.; Bruns, K.; Lackner, K.J.; Pfeiffer, N. Transthyretin and complex protein pattern in aqueous humor of patients with primary open-angle glaucoma. *Mol. Vis.* **2008**, *14*, 1437–1445.
16. Mokbel, T.H.; Ghanem, A.A.; Kishk, H.; Arafa, L.F.; El-Baiomy, A.A. Erythropoietin and soluble CD44 levels in patients with primary open-angle glaucoma. *Clin. Exp. Ophthalmol.* **2010**, *38*, 560–565, doi:10.1111/j.1442-9071.2010.02318.x.
17. Izzotti, A.; Longobardi, M.; Cartiglia, C.; Saccà, S.C. Proteome alterations in primary open angle glaucoma aqueous humor. *J. Proteome Res.* **2010**, *9*, 4831–4838, doi:10.1021/pr1005372.
18. Duan, X.; Xue, P.; Wang, N.; Dong, Z.; Lu, Q.; Yang, F. Proteomic analysis of aqueous humor from patients with primary open angle glaucoma. *Mol. Vis.* **2010**, *16*, 2839–2846.
19. Ghanem, A.A.; Arafa, L.F.; El-Baz, A. Oxidative stress markers in patients with primary open-angle glaucoma. *Curr. Eye Res.* **2010**, *35*, 295–301, doi:10.3109/02713680903548970.
20. Bai, Y.; Sivori, D.; Woo, S.B.; Neet, K.E.; Fabian Lerner, S.; Uri Saragovi, H. During glaucoma, α2-macroglobulin accumulates in aqueous humor and binds to nerve growth factor, neutralizing neuroprotection. *Investig. Ophthalmol. Vis. Sci.* **2011**, *52*, 5260–5265, doi:10.1167/iovs.10-6691.
21. Ghanem, A.A.; Arafa, L.F.; El-Baz, A. Connective tissue growth factor and tissue inhibitor of matrix metalloproteinase-2 in patients with exfoliative glaucoma. *Curr. Eye Res.* **2011**, *36*, 540–545, doi:10.3109/02713683.2011.565541.
22. Balaiya, S.; Edwards, J.; Tillis, T.; Khetpal, V.; Chalam, K. V. Tumor necrosis factor-alpha (TNF-α) levels in aqueous humor of primary open angle glaucoma. *Clin. Ophthalmol.* **2011**, *5*, 553–556, doi:10.2147/OPTH.S19453.
23. Bouhenni, R.A.; Al Shahwan, S.; Morales, J.; Wakim, B.T.; Chomyk, A.M.; Alkuraya, F.S.; Edward, D.P. Identification of differentially expressed proteins in the aqueous humor of primary congenital glaucoma. *Exp. Eye Res.* **2011**, *92*, 67–75, doi:10.1016/j.exer.2010.11.004.
24. Browne, J.G.; Ho, S.L.; Kane, R.; Oliver, N.; Clark, A.F.; O'Brien, C.J.; Crean, J.K. Connective tissue growth factor is increased in pseudoexfoliation glaucoma. *Investig. Ophthalmol. Vis. Sci.* **2011**, *52*, 3660–3666, doi:10.1167/iovs.10-5209.
25. Takai, Y.; Tanito, M.; Ohira, A. Multiplex cytokine analysis of aqueous humor in eyes with primary open-angle glaucoma, exfoliation glaucoma, and cataract. *Investig. Ophthalmol. Vis. Sci.* **2012**, *53*, 241–247, doi:10.1167/iovs.11-8434.
26. Bagnis, A.; Izzotti, A.; Centofanti, M.; Saccà, S.C. Aqueous humor oxidative stress proteomic levels in primary open angle

glaucoma. *Exp. Eye Res.* **2012**, *103*, 55–62, doi:10.1016/j.exer.2012.07.011.

27. Saccà, S.C.; Centofanti, M.; Izzotti, A. New proteins as vascular biomarkers in primary open angle glaucomatous aqueous humor. *Investig. Ophthalmol. Vis. Sci.* **2012**, *53*, 4242–4253, doi:10.1167/iovs.11-8902.
28. Inoue, T.; Kawaji, T.; Tanihara, H. Elevated levels of multiple biomarkers of alzheimer's disease in the aqueous humor of eyes with open-angle glaucoma. *Investig. Ophthalmol. Vis. Sci.* **2013**, *54*, 5353–5358, doi:10.1167/iovs.13-12245.
29. Goyal, A.; Srivastava, A.; Sihota, R.; Kaur, J. Evaluation of oxidative stress markers in aqueous humor of primary open angle glaucoma and primary angle closure glaucoma patients. *Curr. Eye Res.* **2014**, *39*, 823–829, doi:10.3109/02713683.2011.556299.
30. Doudevski, I.; Rostagno, A.; Cowman, M.; Liebmann, J.; Ritch, R.; Ghiso, J. Clusterin and complement activation in exfoliation glaucoma. *Investig. Ophthalmol. Vis. Sci.* **2014**, *55*, 2491–2499, doi:10.1167/iovs.13-12941.
31. Ahoor, M.H.; Ghorbanihaghjo, A.; Sorkhabi, R.; Kiavar, A. Klotho and endothelin-1 in pseudoexfoliation syndrome and glaucoma. *J. Glaucoma* **2016**, *25*, 919–922, doi:10.1097/IJG.0000000000000553.
32. Kliuchnikova, A.A.; Samokhina, N.I.; Ilina, I.Y.; Karpov, D.S.; Pyatnitskiy, M.A.; Kuznetsova, K.G.; Toropygin, I.Y.; Kochergin, S.A.; Alekseev, I.B.; Zgoda, V.G.; et al. Human aqueous humor proteome in cataract, glaucoma, and pseudoexfoliation syndrome. *Proteomics* **2016**, *16*, 1938–1946, doi:10.1002/pmic.201500423.
33. Kaeslin, M.A.; Killer, H.E.; Fuhrer, C.A.; Zeleny, N.; Huber, A.R.; Neutzner, A. Changes to the aqueous humor proteome during glaucoma. *PLoS One* **2016**, *11*, 1–15, doi:10.1371/journal.pone.0165314.
34. Baumane, K.; Ranka, R.; Laganovska, G. Association of NT-proANP Level in Plasma and Humor Aqueous with Primary Open-Angle Glaucoma. *Curr. Eye Res.* **2017**, *42*, 233–236, doi:10.1080/02713683.2016.1180397.
35. Hondur, G.; Göktaşş, E.; Yang, X.; Al-Aswad, L.; Auran, J.D.; Blumberg, D.M.; Cioffi, G.A.; Liebmann, J.M.; Suh, L.H.; Trief, D.; et al. Oxidative stress–related molecular biomarker candidates for glaucoma. *Investig. Ophthalmol. Vis. Sci.* **2017**, *58*, 4078–4088, doi:10.1167/iovs.17-22242.
36. Garweg, J.G.; Zandi, S.; Pfister, I.B.; Skowronska, M.; Gerhardt, C. Comparison of cytokine profiles in the aqueous humor of eyes with pseudoexfoliation syndrome and glaucoma. *PLoS One* **2017**, *12*, 1–13, doi:10.1371/journal.pone.0182571.
37. Ban, N.; Siegfried, C.J.; Lin, J.B.; Shui, Y.B.; Sein, J.; Pita-Thomas, W.; Sene, A.; Santeford, A.; Gordon, M.; Lamb, R.; et al. GDF15 is elevated in mice following retinal ganglion cell death and in glaucoma patients. *JCI insight* **2017**, *2*, doi:10.1172/jci.insight.91455.
38. Zhang, Y.; Yang, Q.; Guo, F.; Chen, X.; Xie, L. Link between neurodegeneration and trabecular meshwork injury in glaucomatous patients. *BMC Ophthalmol.* **2017**, *17*, 1–7, doi:10.1186/s12886-017-0623-z.
39. Sharma, S.; Bollinger, K.E.; Kodeboyina, S.K.; Zhi, W.; Patton, J.; Bai, S.; Edwards, B.; Ulrich, L.; Bogorad, D.; Sharma, A. Proteomic alterations in aqueous humor from patients with primary open angle glaucoma. *Investig. Ophthalmol. Vis. Sci.* **2018**, *59*, 2635–2643, doi:10.1167/iovs.17-23434.
40. Adav, S.S.; Wei, J.; Terence, Y.; Ang, B.C.H.; Yip, L.W.L.; Sze, S.K. Proteomic Analysis of Aqueous Humor from Primary Open Angle Glaucoma Patients on Drug Treatment Revealed Altered Complement Activation Cascade. *J. Proteome Res.* **2018**, *17*, 2499–2510, doi:10.1021/acs.jproteome.8b00244.
41. Wang, J.; Fu, M.; Liu, K.; Wang, N.; Zhang, Z.; Zhou, M.; Xu, X. Matricellular Proteins Play a Potential Role in Acute Primary Angle Closure. *Curr. Eye Res.* **2018**, *43*, 771–777, doi:10.1080/02713683.2018.1449222.
42. Kaur, I.; Kaur, J.; Sooraj, K.; Goswami, S.; Saxena, R.; Chauhan, V.S.; Sihota, R. Comparative evaluation of the aqueous humor proteome of primary angle closure and primary open angle glaucomas and age-related cataract eyes. *Int. Ophthalmol.* **2019**, *39*, 69–104, doi:10.1007/s10792-017-0791-0.
43. Nikhalashree, S.; George, R.; Shantha, B.; Lingam, V.; Vidya, W.; Panday, M.; Sulochana, K.N.; Coral, K. Detection of Proteins Associated with Extracellular Matrix Regulation in the Aqueous Humour of Patients with Primary Glaucoma.

Curr. Eye Res. **2019**, *44*, 1018–1025, doi:10.1080/02713683.2019.1608261.

44. Guo, T.; Guo, L.; Fan, Y.; Fang, L.; Wei, J.; Tan, Y.; Chen, Y.; Fan, X. Aqueous humor levels of TGFβ2 and SFRP1 in different types of glaucoma. *BMC Ophthalmol.* **2019**, *19*, 1–9, doi:10.1186/s12886-019-1183-1.
45. Can Demirdöğen, B.; Koçan Akçin, C.; Özge, G.; Mumcuoğlu, T. Evaluation of tear and aqueous humor level, and genetic variants of connective tissue growth factor as biomarkers for early detection of pseudoexfoliation syndrome/glaucoma. *Exp. Eye Res.* **2019**, *189*, 107837, doi:10.1016/j.exer.2019.107837.
46. ten Berge, J.C.; Fazil, Z.; van den Born, I.; Wolfs, R.C.W.; Schreurs, M.W.J.; Dik, W.A.; Rothova, A. Intraocular cytokine profile and autoimmune reactions in retinitis pigmentosa, age-related macular degeneration, glaucoma and cataract. *Acta Ophthalmol.* **2019**, *97*, 185–192, doi:10.1111/aos.13899.
47. Basu, K.; Maurya, N.; Kaur, J.; Saxena, R.; Gupta, V.; Sihota, R.; Ghosh, I. Possible role of differentially expressing novel protein markers (ligatin and fibulin-7) in human aqueous humor and trabecular meshwork tissue in glaucoma progression. *Cell Biol. Int.* **2019**, *43*, 820–834, doi:10.1002/cbin.11138.
48. Can Demirdöğen, B.; Demirkaya-Budak, S.; Özge, G.; Mumcuoğlu, T. Evaluation of Tear Fluid and Aqueous Humor Concentration of Clusterin as Biomarkers for Early Diagnosis of Pseudoexfoliation Syndrome and Pseudoexfoliative Glaucoma. *Curr. Eye Res.* **2020**, *45*, 805–813, doi:10.1080/02713683.2019.1698055.
49. Sun, C.; Zhang, H.; Tang, Y.; Chen, Y.; Li, Y.; Nie, C.; Gu, J.; Luo, L.; Wang, Z. Aqueous Inflammation and Ischemia-Related Biomarkers in Neovascular Glaucoma with Stable Iris Neovascularization. *Curr. Eye Res.* **2020**, *45*, 1504–1513, doi:10.1080/02713683.2020.1762226.
50. Sun, C.; Zhang, H.; Jiang, J.; Li, Y.; Nie, C.; Gu, J.; Luo, L.; Wang, Z. Angiogenic and inflammatory biomarker levels in aqueous humor and vitreous of neovascular glaucoma and proliferative diabetic retinopathy. *Int. Ophthalmol.* **2020**, *40*, 467–475, doi:10.1007/s10792-019-01207-4.
51. Ishikawa, K.; Kohno, R. ichiro; Mori, K.; Murakami, Y.; Nakao, S.; Akiyama, M.; Yoshida, S.; Sonoda, K.H. Increased expression of periostin and tenascin-C in eyes with neovascular glaucoma secondary to PDR. *Graefe's Arch. Clin. Exp. Ophthalmol.* **2020**, *258*, 621–628, doi:10.1007/s00417-019-04574-x.
52. Hubens, W.H.G.; Mohren, R.J.C.; Liesenborghs, I.; Eijssen, L.M.T.; Ramdas, W.D.; Webers, C.A.B.; Gorgels, T.G.M.F. The aqueous humor proteome of primary open angle glaucoma: An extensive review. *Exp. Eye Res.* **2020**, *197*, 108077, doi:10.1016/j.exer.2020.108077.
53. Lin, J.B.; Sheybani, A.; Santeford, A.; Maria, A. De; Apte, R.S. Increased aqueous humor gdf15 is associated with worse visual field loss in pseudoexfoliative glaucoma patients. *Transl. Vis. Sci. Technol.* **2020**, *9*, 1–6, doi:10.1167/tvst.9.10.16.
54. Burgos-Blasco, B.; Vidal-Villegas, B.; Saenz-Frances, F.; Morales-Fernandez, L.; Perucho-Gonzalez, L.; Garcia-Feijoo, J.; Martinez-de-la-Casa, J.M. Tear and aqueous humour cytokine profile in primary open-angle glaucoma. *Acta Ophthalmol.* **2020**, *98*, e768–e772, doi:10.1111/aos.14374.
55. Nezu, N.; Usui, Y.; Saito, A.; Shimizu, H.; Asakage, M.; Yamakawa, N.; Tsubota, K.; Wakabayashi, Y.; Narimatsu, A.; Umazume, K.; et al. Machine Learning Approach for Intraocular Disease Prediction Based on Aqueous Humor Immune Mediator Profiles. *Ophthalmology* **2021**, doi:10.1016/j.ophtha.2021.01.019.
56. Igarashi, N.; Honjo, M.; Asaoka, R.; Kurano, M.; Yatomi, Y.; Igarashi, K.; Miyata, K.; Kaburaki, T.; Aihara, M. Aqueous autotaxin and TGF-βs are promising diagnostic biomarkers for distinguishing open-angle glaucoma subtypes. *Sci. Rep.* **2021**, *11*, 1–9, doi:10.1038/s41598-021-81048-3.
57. Kotikoski, H.; Moilanen, E.; Vapaatalo, H.; Aine, E. Biochemical markers of the L-arginine-nitric oxide pathway in the aqueous humour in glaucoma patients. *Acta Ophthalmol. Scand.* **2002**, *80*, 191–195, doi:10.1034/j.1600-0420.2002.800214.x.
58. Galassi, F.; Renieri, G.; Sodi, A.; Ucci, F.; Vannozzi, L.; Masini, E. Nitric oxide proxies and ocular perfusion pressure in primary open angle glaucoma. *Br. J. Ophthalmol.* **2004**, *88*, 757–760, doi:10.1136/bjo.2003.028357.

59. Bleich, S.; Roedl, J.; Von Ahsen, N.; Schlötzer-Schrehardt, U.; Reulbach, U.; Beck, G.; Kruse, F.E.; Naumann, G.O.H.; Kornhuber, J.; Jünemann, A.G.M. Elevated homocysteine levels in aqueous humor of patients with pseudoexfoliation glaucoma. *Am. J. Ophthalmol.* **2004**, *138*, 162–164, doi:10.1016/j.ajo.2004.02.027.
60. Castany, M.; Jordi, I.; Catala, J.; Gual, A.; Morales, M.; Gasull, X.; Pintor, J. Glaucoma patients present increased levels of diadenosine tetraphosphate, Ap4A, in the aqueous humour. *Exp. Eye Res.* **2011**, *92*, 221–226, doi:10.1016/j.exer.2010.12.004.
61. Nucci, C.; Di Pierro, D.; Varesi, C.; Ciuffoletti, E.; Russo, R.; Gentile, R.; Cedrone, C.; Duran, M.D.P.; Coletta, M.; Mancino, R. Increased malondialdehyde concentration and reduced total antioxidant capacity in aqueous humor and blood samples from patients with glaucoma. *Mol. Vis.* **2013**, *19*, 1841–1846.
62. Buisset, A.; Gohier, P.; Leruez, S.; Muller, J.; Amati-Bonneau, P.; Lenaers, G.; Bonneau, D.; Simard, G.; Procaccio, V.; Annweiler, C.; et al. Metabolomic Profiling of Aqueous Humor in Glaucoma Points to Taurine and Spermine Deficiency: Findings from the Eye-D Study. *J. Proteome Res.* **2019**, *18*, 1307–1315, doi:10.1021/acs.jproteome.8b00915.
63. Chen, X.; Chen, Y.; Wang, L.; Sun, X. Metabolomics of the aqueous humor in patients with primary congenital glaucoma. *Mol. Vis.* **2019**, *25*, 489–501.
64. Pan, C.W.; Ke, C.; Chen, Q.; Tao, Y.J.; Zha, X.; Zhang, Y.P.; Zhong, H. Differential metabolic markers associated with primary open-angle glaucoma and cataract in human aqueous humor. *BMC Ophthalmol.* **2020**, *20*, 1–8, doi:10.1186/s12886-020-01452-7.
65. Barbosa Breda, J.; Croitor Sava, A.; Himmelreich, U.; Somers, A.; Matthys, C.; Rocha Sousa, A.; Vandewalle, E.; Stalmans, I. Metabolomic profiling of aqueous humor from glaucoma patients - The metabolomics in surgical ophthalmological patients (MISO) study. *Exp. Eye Res.* **2020**, *201*, 108268, doi:10.1016/j.exer.2020.108268.
66. Tezel, G.; Li, L.Y.; Patil, R. V.; Wax, M.B. TNF-α and TNF-α receptor-1 in the retina of normal and glaucomatous eyes. *Investig. Ophthalmol. Vis. Sci.* **2001**, *42*, 1787–1794.
67. Tezel, G.; Wax, M.B. Hypoxia-inducible factor 1alpha in the glaucomatous retina and optic nerve head. *Arch. Ophthalmol. (Chicago, Ill. 1960)* **2004**, *122*, 1348–1356, doi:10.1001/archopht.122.9.1348.
68. Bhattacharya, S.K.; Rockwood, E.J.; Smith, S.D.; Bonilha, V.L.; Crabb, J.S.; Kuchtey, R.W.; Robertson, N.G.; Peachey, N.S.; Morton, C.C.; Crabb, J.W. Proteomics reveal cochlin deposits associated with glaucomatous trabecular meshwork. *J. Biol. Chem.* **2005**, *280*, 6080–6084, doi:10.1074/jbc.M411233200.
69. Bhattacharya, S.K.; Crabb, J.S.; Bonilha, V.L.; Gu, X.; Takahara, H.; Crabb, J.W. Proteomics implicates peptidyl arginine deiminase 2 and optic nerve citrullination in glaucoma pathogenesis. *Investig. Ophthalmol. Vis. Sci.* **2006**, *47*, 2508–2514, doi:10.1167/iovs.05-1499.
70. Zhang, Y.; Gao, Q.; Duan, S.; He, Y.; Sun, X.; Jiang, R.; Duan, Y.; Zhong, X.; Ge, J. Upregulation of Copine1 in trabecular meshwork cells of POAG patients: A membrane proteomics approach. *Mol. Vis.* **2008**, *14*, 1028–1036.
71. Govindarajan, B.; Laird, J.; Salomon, R.G.; Bhattacharya, S.K. Isolevuglandin-modified proteins, including elevated levels of inactive calpain-1, accumulate in glaucomatous trabecular meshwork. *Biochemistry* **2008**, *47*, 817–825, doi:10.1021/bi701517m.
72. Tezel, G.; Yang, X.; Luo, C.; Cai, J.; Kain, A.D.; Powell, D.W.; Kuehn, M.H.; Pierce, W.M. Hemoglobin expression and regulation in glaucoma: Insights into retinal ganglion cell oxygenation. *Investig. Ophthalmol. Vis. Sci.* **2010**, *51*, 907–919, doi:10.1167/iovs.09-4014.
73. Tezel, G.; Yang, X.; Luo, C.; Kain, A.D.; Powell, D.W.; Kuehn, M.H.; Kaplan, H.J. Oxidative stress and the regulation of complement activation in human glaucoma. *Investig. Ophthalmol. Vis. Sci.* **2010**, *51*, 5071–5082, doi:10.1167/iovs.10-5289.
74. Yang, X.; Luo, C.; Cai, J.; Powell, D.W.; Yu, D.; Kuehn, M.H.; Tezel, G. Neurodegenerative and inflammatory pathway components linked to TNF-α/TNFR1 signaling in the glaucomatous human retina. *Investig. Ophthalmol. Vis. Sci.* **2011**, *52*, 8442–8454, doi:10.1167/iovs.11-8152.

75. Mizokami, J.; Kanamori, A.; Negi, A.; Nakamura, M. A preliminary study of reduced expression of aquaporin-9 in the optic nerve of primate and human eyes with glaucoma. *Curr. Eye Res.* **2011**, *36*, 1064–1067, doi:10.3109/02713683.2011.611610.
76. Kovacs, K.; Marra, K. V.; Yu, G.; Wagley, S.; Ma, J.; Teague, G.C.; Nandakumar, N.; Lashkari, K.; Arroyo, J.G. Angiogenic and inflammatory vitreous biomarkers associated with increasing levels of retinal ischemia. *Investig. Ophthalmol. Vis. Sci.* **2015**, *56*, 6523–6530, doi:10.1167/iovs.15-16793.
77. Inafuku, S.; Noda, K.; Amano, M.; Nishimura, S.I.; Ishida, S. Short Communication: Increase of Sialylated N-Glycansin Eyes with Neovascular Glaucoma Secondary to Proliferative Diabetic Retinopathy. *Curr. Eye Res.* **2016**, *41*, 721–724, doi:10.3109/02713683.2015.1068816.
78. Funke, S.; Perumal, N.; Beck, S.; Gabel-Scheurich, S.; Schmelter, C.; Teister, J.; Gerbig, C.; Gramlich, O.W.; Pfeiffer, N.; Grus, F.H. Glaucoma related proteomic alterations in human retina samples. *Sci. Rep.* **2016**, *6*, 1–18, doi:10.1038/srep29759.
79. Micera, A.; Quaranta, L.; Esposito, G.; Floriani, I.; Pocobelli, A.; Saccà, S.C.; Riva, I.; Manni, G.; Oddone, F. Differential Protein Expression Profiles in Glaucomatous Trabecular Meshwork: An Evaluation Study on a Small Primary Open Angle Glaucoma Population. *Adv. Ther.* **2016**, *33*, 252–267, doi:10.1007/s12325-016-0285-x.
80. Tong, Y.; Zhou, Y.L.; Zheng, Y.; Biswal, M.; Zhao, P.Q.; Wang, Z.Y. Analyzing cytokines as biomarkers to evaluate severity of glaucoma. *Int. J. Ophthalmol.* **2017**, *10*, 925–930, doi:10.18240/ijo.2017.06.15.
81. Mirzaei, M.; Gupta, V.B.; Chick, J.M.; Greco, T.M.; Wu, Y.; Chitranshi, N.; Wall, R. Vander; Hone, E.; Deng, L.; Dheer, Y.; et al. Age-related neurodegenerative disease associated pathways identified in retinal and vitreous proteome from human glaucoma eyes. *Sci. Rep.* **2017**, *7*, 1–16, doi:10.1038/s41598-017-12858-7.
82. Dreyer, E.B.; Zurakowski, D.; Schumer, R.A.; Podos, S.M.; Lipton, S.A. Elevated glutamate levels in the vitreous body of humans and monkeys with glaucoma. *Arch. Ophthalmol. (Chicago, Ill. 1960)* **1996**, *114*, 299–305, doi:10.1001/archopht.1996.01100130295012.
83. Doganay, S.; Cankaya, C.; Alkan, A. Evaluation of corpus geniculatum laterale and vitreous fluid by magnetic resonance spectroscopy in patients with glaucoma; A preliminary study. *Eye* **2012**, *26*, 1044–1051, doi:10.1038/eye.2012.84.
84. Ghaffariyeh, A.; Honarpisheh, N.; Shakiba, Y.; Puyan, S.; Chamacham, T.; Zahedi, F.; Zarrineghbal, M. Brain-derived neurotrophic factor in patients with normal-tension glaucoma. *Optometry* **2009**, *80*, 635–638, doi:10.1016/j.optm.2008.09.014.
85. Pieragostino, D.; Bucci, S.; Agnifili, L.; Fasanella, V.; D'Aguanno, S.; Mastropasqua, A.; Ciancaglini, M.; Mastropasqua, L.; Di Ilio, C.; Sacchetta, P.; et al. Differential protein expression in tears of patients with primary open angle and pseudoexfoliative glaucoma. *Mol. Biosyst.* **2012**, *8*, 1017–1028, doi:10.1039/c1mb05357d.
86. Pieragostino, D.; Agnifili, L.; Fasanella, V.; D'Aguanno, S.; Mastropasqua, R.; Di Ilio, C.; Sacchetta, P.; Urbani, A.; Del Boccio, P. Shotgun proteomics reveals specific modulated protein patterns in tears of patients with primary open angle glaucoma naïve to therapy. *Mol. Biosyst.* **2013**, *9*, 1108–1116, doi:10.1039/c3mb25463a.
87. Gupta, D.; Wen, J.C.; Huebner, J.L.; Stinnett, S.; Kraus, V.B.; Tseng, H.C.; Walsh, M. Cytokine biomarkers in tear film for primary open-angle glaucoma. *Clin. Ophthalmol.* **2017**, *11*, 411–416, doi:10.2147/OPTH.S125364.
88. Sahay, P.; Rao, A.; Padhy, D.; Sarangi, S.; Das, G.; Reddy, M.M.; Modak, R. Functional activity of matrix metalloproteinases 2 and 9 in tears of patients with glaucoma. *Investig. Ophthalmol. Vis. Sci.* **2017**, *58*, 106–113, doi:10.1167/iovs.17-21723.
89. Shpak, A.A.; Guekht, A.B.; Druzhkova, T.A.; Kozlova, K.I.; Gulyaeva, N. V. Ciliary neurotrophic factor in patients with primary open-angle glaucoma and age-related cataract. *Mol. Vis.* **2017**, *23*, 799–809.
90. Martinez-de-la-Casa, J.M.; Perez-Bartolome, F.; Urcelay, E.; Santiago, J.L.; Moreno-Montañes, J.; Arriola-Villalobos, P.; Benitez-del-Castillo, J.M.; Garcia-Feijoo, J. Tear cytokine profile of glaucoma patients treated with preservative-free or preserved latanoprost. *Ocul. Surf.* **2017**, *15*, 723–729, doi:10.1016/j.jtos.2017.03.004.
91. Reddy, S.; Sahay, P.; Padhy, D.; Sarangi, S.; Suar, M.; Modak, R.; Rao, A. Tear biomarkers in latanoprost and bimatoprost treated eyes. *PLoS One* **2018**, *13*, 1–13, doi:10.1371/journal.pone.0201740.

92. Csősz, É.; Deák, E.; Tóth, N.; Traverso, C.E.; Csutak, A.; Tőzsér, J. Comparative analysis of cytokine profiles of glaucomatous tears and aqueous humour reveals potential biomarkers for trabeculectomy complications. *FEBS Open Bio* **2019**, *9*, 1020–1028, doi:10.1002/2211-5463.12637.
93. Sedlak, L.; Wojnar, W.; Zych, M.; Wyględowska-Promieńska, D. Influence of timolol, benzalkonium-preserved timolol, and benzalkonium-preserved brimonidine on oxidative stress biomarkers in the tear film. *Cutan. Ocul. Toxicol.* **2020**, *0*, 1–9, doi:10.1080/15569527.2020.1787435.
94. Roedl, J.B.; Bleich, S.; Reulbach, U.; Rejdak, R.; Kornhuber, J.; Kruse, F.E.; Schlötzer-Schrehardt, U.; Jünemann, A.G. Homocysteine in tear fluid of patients with pseudoexfoliation glaucoma. *J. Glaucoma* **2007**, *16*, 234–239, doi:10.1097/IJG.0b013e31802d6942.
95. Rossi, C.; Cicalini, I.; Cufaro, M.C.; Agnifili, L.; Mastropasqua, L.; Lanuti, P.; Marchisio, M.; De Laurenzi, V.; Del Boccio, P.; Pieragostino, D. Multi-omics approach for studying tears in treatment-naïve glaucoma patients. *Int. J. Mol. Sci.* **2019**, *20*, 1–14, doi:10.3390/ijms20164029.
96. Weinstein, B.I.; Iyer, R.B.; Binstock, J.M.; Hamby, C. V.; Schwartz, I.S.; Moy, F.H.; Wandel, T.; Southren, A.L. Decreased 3α-hydroxysteroid dehydrogenase activity in peripheral blood lymphocytes from patients with primary open angle glaucoma. *Exp. Eye Res.* **1996**, *62*, 39–46, doi:10.1006/exer.1996.0005.
97. Tezel, G.; Edward, D.P.; Wax, M.B. Serum autoantibodies to optic nerve head glycosaminoglycans in patients with glaucoma. *Arch. Ophthalmol.* **1999**, *117*, 917–924, doi:10.1001/archopht.117.7.917.
98. Zabala, L.; Saldanha, C.; Martins e Silva, J.; Souza-Ramalho, P. Red blood cell membrane integrity in primary open angle glaucoma : vivo and in vitro studies. *Eye (Lond).* **1999**, *13 (Pt 1)*, 101–103, doi:10.1038/eye.1999.18.
99. Maruyama, I.; Ohguro, H.; Ikeda, Y. Retinal ganglion cells recognized by serum autoantibody against gamma-enolase found in glaucoma patients. *Invest. Ophthalmol. Vis. Sci.* **2000**, *41*, 1657–1665.
100. Yang, J.; Patil, R. V.; Yu, H.; Gordon, M.; Wax, M.B. T cell subsets and sIL-2R/IL-2 levels in patients with glaucoma. *Am. J. Ophthalmol.* **2001**, *131*, 421–426, doi:10.1016/S0002-9394(00)00862-X.
101. Yang, J.; Tezel, G.; Patil, R. V.; Romano, C.; Wax, M.B. Serum autoantibody against glutathione S-transferase in patients with glaucoma. *Investig. Ophthalmol. Vis. Sci.* **2001**, *42*, 1273–1276.
102. Kremmer, S.; Kreuzfelder, E.; Klein, R.; Bontke, N.; Henneberg-Quester, K.B.; Steuhl, K.P.; Grosse-Wilde, H. Antiphosphatidylserine antibodies are elevated in normal tension glaucoma. *Clin. Exp. Immunol.* **2001**, *125*, 211–215, doi:10.1046/j.1365-2249.2001.01578.x.
103. P.L., L.; D.C., F.; A.D., B.; N., M.; S., T.; I.A., C.; G.Y.H., L.; Lip, P.L.; Felmeden, D.C.; Blann, A.D.; et al. Plasma vascular endothelial growth factor, soluble VEGF receptor FLT-1, and von Willebrand factor in glaucoma. *Br. J. Ophthalmol.* **2002**, *86*, 1299–1302.
104. Wunderlich, K.; Golubnitschaja, O.; Pache, M.; Eberle, A.N.; Flammer, J. Increased plasma levels of 20S proteasome α-subunit in glaucoma patients: An observational pilot study. *Mol. Vis.* **2002**, *8*, 431–435.
105. Golubnitschaja, O.; Yeghiazaryan, K.; Liu, R.; Mönkemann, H.; Leppert, D.; Schild, H.; Haefliger, I.O.; Flammer, J. Increased Expression of Matrix Metalloproteinases in Mononuclear Blood Cells of Normal-Tension Glaucoma Patients. *J. Glaucoma* **2004**, *13*, 66–72, doi:10.1097/00061198-200402000-00013.
106. Emre, M.; Orgül, S.; Haufschild, T.; Shaw, S.G.; Flammer, J. Increased plasma endothelin-1 levels in patients with progressive open angle glaucoma. *Br. J. Ophthalmol.* **2005**, *89*, 60–63, doi:10.1136/bjo.2004.046755.
107. Gherghel, D.; Griffiths, H.R.; Hilton, E.J.; Cunliffe, I.A.; Hosking, S.L. Systemic reduction in glutathione levels occurs in patients with primary open-angle glaucoma. *Investig. Ophthalmol. Vis. Sci.* **2005**, *46*, 877–883, doi:10.1167/iovs.04-0777.
108. Yildirim, Ö.; Ateş, N.A.; Ercan, B.; Muşlu, N.; Ünlü, A.; Tamer, L.; Atik, U.; Kanik, A. Role of oxidative stress enzymes in open-angle glaucoma. *Eye* **2005**, *19*, 580–583, doi:10.1038/sj.eye.6701565.

109. Noureddin, B.N.; Al-Haddad, C.E.; Bashshur, Z.; Safieh-Garabedian, B. Plasma thymulin and nerve growth factor levels in patients with primary open angle glaucoma and elevated intraocular pressure. *Graefe's Arch. Clin. Exp. Ophthalmol.* **2006**, *244*, 750–752, doi:10.1007/s00417-005-0143-z.
110. Grus, F.H.; Joachim, S.C.; Bruns, K.; Lackner, K.J.; Pfeiffer, N.; Wax, M.B. Serum autoantibodies to α-fodrin are present in glaucoma patients from Germany and the United States. *Investig. Ophthalmol. Vis. Sci.* **2006**, *47*, 968–976, doi:10.1167/iovs.05-0685.
111. Acar, N.; Berdeaux, O.; Juaneda, P.; Grégoire, S.; Cabaret, S.; Joffre, C.; Creuzot-Garcher, C.P.; Bretillon, L.; Bron, A.M. Red blood cell plasmalogens and docosahexaenoic acid are independently reduced in primary open-angle glaucoma. *Exp. Eye Res.* **2009**, *89*, 840–853, doi:10.1016/j.exer.2009.07.008.
112. Huang, P.; Qi, Y.; Xu, Y.S.; Liu, J.; Liao, D.; Zhang, S.S.M.; Zhang, C. Serum cytokine alteration is associated with optic neuropathy in human primary open angle glaucoma. *J. Glaucoma* **2010**, *19*, 324–330, doi:10.1097/IJG.0b013e3181b4cac7.
113. Engin, K.N.; Yemişci, B.; Yiğit, U.; Ağaçhan, A.; Coşkun, C. Variability of serum oxidative stress biomarkers relative to biochemical data and clinical parameters of glaucoma patients. *Mol. Vis.* **2010**, *16*, 1260–1271.
114. Sorkhabi, R.; Ghorbanihaghjo, A.; Javadzadeh, A.; Rashtchizadeh, N.; Moharrery, M. Oxidative DNA damage and total antioxidant status in glaucoma patients. *Mol. Vis.* **2011**, *17*, 41–46.
115. Chang, D.; Sha, Q.; Zhang, X.; Liu, P.; Rong, S.; Han, T.; Liu, P.; Pan, H. The evaluation of the oxidative stress parameters in patients with primary angle-closure glaucoma. *PLoS One* **2011**, *6*, 4–9, doi:10.1371/journal.pone.0027218.
116. Majsterek, I.; Malinowska, K.; Stanczyk, M.; Kowalski, M.; Blaszczyk, J.; Kurowska, A.K.; Kaminska, A.; Szaflik, J.; Szaflik, J.P. Evaluation of oxidative stress markers in pathogenesis of primary open-angle glaucoma. *Exp. Mol. Pathol.* **2011**, *90*, 231–237, doi:10.1016/j.yexmp.2011.01.001.
117. Ghaffariyeh, A.; Honarpisheh, N.; Heidari, M.H.; Puyan, S.; Abasov, F. Brain-derived neurotrophic factor as a biomarker in primary open-angle glaucoma. *Optom. Vis. Sci.* **2011**, *88*, 80–85, doi:10.1097/OPX.0b013e3181fc329f.
118. Tanito, M.; Kaidzu, S.; Takai, Y.; Ohira, A. Status of Systemic Oxidative Stresses in Patients with Primary Open-Angle Glaucoma and Pseudoexfoliation Syndrome. *PLoS One* **2012**, *7*, 1–7, doi:10.1371/journal.pone.0049680.
119. Tezel, G.; Thornton, I.L.; Tong, M.G.; Luo, C.; Yang, X.; Cai, J.; Powell, D.W.; Soltau, J.B.; Liebmann, J.M.; Ritch, R. Immunoproteomic analysis of potential serum biomarker candidates in human glaucoma. *Investig. Ophthalmol. Vis. Sci.* **2012**, *53*, 8222–8231, doi:10.1167/iovs.12-10076.
120. Zanon-Moreno, V.; Asensio-Marquez, E.M.; Ciancotti-Oliver, L.; Garcia-Medina, J.J.; Sanz, P.; Ortega-Azorin, C.; Pinazo-Duran, M.D.; Ordovás, J.M.; Corella, D. Effects of polymorphisms in vitamin E-, vitamin C-, and glutathione peroxidase-related genes on serum biomarkers and associations with glaucoma. *Mol. Vis.* **2013**, *19*, 231–242.
121. Abu-Amero, K.K.; Kondkar, A.A.; Mousa, A.; Osman, E.A.; Al-Obeidan, S.A. Decreased total antioxidants in patients with primary open angle glaucoma. *Curr. Eye Res.* **2013**, *38*, 959–964, doi:10.3109/02713683.2013.794246.
122. López-Riquelme, N.; Villalba, C.; Tormo, C.; Belmonte, A.; Fernandez, C.; Torralba, G.; Hernández, F. Endothelin-1 levels and biomarkers of oxidative stress in glaucoma patients. *Int. Ophthalmol.* **2014**, *35*, 527–532, doi:10.1007/s10792-014-9979-8.
123. González-Iglesias, H.; Álvarez, L.; García, M.; Escribano, J.; Rodríguez-Calvo, P.P.; Fernández-Vega, L.; Coca-Prados, M. Comparative proteomic study in serum of patients with primary open-angle glaucoma and pseudoexfoliation glaucoma. *J. Proteomics* **2014**, *98*, doi:10.1016/j.jprot.2013.12.006.
124. Ozgonul, C.; Sertoglu, E.; Mumcuoglu, T.; Kucukevcilioglu, M. Neutrophil-to-lymphocyte ratio and platelet-to-lymphocyte ratio as novel biomarkers of primary open-angle glaucoma. *J. Glaucoma* **2016**, *25*, e815–e820, doi:10.1097/IJG.0000000000000392.
125. Kurtul, B.E.; Kurtul, A.; Altiaylik Ozer, P.; Kabatas, E.U.; Ertugrul, G.T. Serum Lipid Levels in Pseudoexfoliation Syndrome. *Semin. Ophthalmol.* **2017**, *32*, 281–284, doi:10.3109/08820538.2015.1068341.

126. Li, S.; Chen, Y.; Shao, M.; Tang, L.; Sun, X.; Cao, W. Association of plasma complement C3 levels with primary angle-closure glaucoma in older women. *Investig. Ophthalmol. Vis. Sci.* **2017**, *58*, 682–689, doi:10.1167/iovs.16-20675.
127. Oddone, F.; Roberti, G.; Micera, A.; Busanello, A.; Bonini, S.; Quaranta, L.; Agnifili, L.; Manni, G. Exploring serum levels of Brain Derived Neurotrophic Factor and Nerve Growth Factor across glaucoma stages. *PLoS One* **2017**, *12*, 1–14, doi:10.1371/journal.pone.0168565.
128. Li, S.; Cao, W.; Han, J.; Tang, B.; Sun, X. The diagnostic value of white blood cell, neutrophil, neutrophil-to-lymphocyte ratio, and lymphocyte-to-monocyte ratio in patients with primary angle closure glaucoma. *Oncotarget* **2017**, *8*, 68984–68995, doi:10.18632/oncotarget.16571.
129. Rokicki, W.; Zalejska-Fiolka, J.; Pojda-Wilczek, D.; Hampel, A.; Majewski, W.; Ogultekin, S.; Mrukwa-Kominek, E. Differences in serum oxidative status between glaucomatous and nonglaucomatous cataract patients. *BMC Ophthalmol.* **2017**, *17*, 1–7, doi:10.1186/s12886-017-0409-3.
130. Kondkar, A.A.; Sultan, T.; Almobarak, F.A.; Kalantan, H.; Al-Obeidan, S.A.; Abu-Amero, K.K. Association of increased levels of plasma tumor necrosis factor alpha with primary open-angle glaucoma. *Clin. Ophthalmol.* **2018**, *12*, 701–706, doi:10.2147/OPTH.S162999.
131. Kondkar, A.A.; Azad, T.A.; Almobarak, F.A.; Kalantan, H.; Al-Obeidan, S.A.; Abu-Amero, K.K. Elevated levels of plasma tumor necrosis factor alpha in patients with pseudoexfoliation glaucoma. *Clin. Ophthalmol.* **2018**, *12*, 153–159, doi:10.2147/OPTH.S155168.
132. Yaz, Y.A.; Yıldırım, N.; Yaz, Y.; Tekin, N.; İnal, M.; Şahin, F.M. Role of oxidative stress in pseudoexfoliation syndrome and pseudoexfoliation glaucoma. *Turkish J. Ophthalmol.* **2019**, *49*, 61–67, doi:10.4274/tjo.galenos.2018.10734.
133. Yang, X.; Zeng, Q.; Göktaş, E.; Gopal, K.; Al-Aswad, L.; Blumberg, D.M.; Cioffi, G.A.; Liebmann, J.M.; Tezel, G. T-lymphocyte subset distribution and activity in patients with glaucoma. *Investig. Ophthalmol. Vis. Sci.* **2019**, *60*, 877–888, doi:10.1167/iovs.18-26129.
134. Abessolo, F.O.; Nnang, J.F.; Aki, T.M.; Amoussa, M.D.E.M.; Ngou-Milama, E. Plasmatic neuroglobin during the primitive open-angle glaucoma. *Ann. Biol. Clin. (Paris).* **2019**, *77*, 99–105, doi:10.1684/abc.2018.1408.
135. Karakurt, Y.; Mertoglu, C.; Gok, G.; Ucak, T.; Tasli, N.; Icel, E.; Erel, O. Thiol–Disulfide Homeostasis and Serum Ischemia Modified Albumin Levels in Patients with Primary Open–Angle Glaucoma. *Curr. Eye Res.* **2019**, *44*, 896–900, doi:10.1080/02713683.2019.1594925.
136. Beutgen, V.M.; Perumal, N.; Pfeiffer, N.; Grus, F.H. Autoantibody biomarker discovery in primary open angle glaucoma using serological proteome analysis (SERPA). *Front. Immunol.* **2019**, *10*, doi:10.3389/fimmu.2019.00381.
137. Maric, V.D.; Bozic, M.M.; Cirkovic, A.M.; Stankovic, S.D.J.; Marjanovic, I.S.; Grgurevic, A.D. Serum heparan sulfate and chondroitin sulfate concentrations in patients with newly diagnosed exfoliative glaucoma. *PeerJ* **2019**, *2019*, 1–20, doi:10.7717/peerj.6920.
138. Igarashi, T.; Nakamoto, K.; Kobayashi, M.; Suzuki, H.; Tobita, Y.; Igarashi, T.; Okuda, T.; Okada, T.; Takahashi, H. Serum brain-derived neurotrophic factor in glaucoma patients in Japan: An observational study. *J. Nippon Med. Sch.* **2020**, *87*, 339–345, doi:10.1272/jnms.JNMS.2020_87-605.
139. Shin, Y.J.; Kim, E.; Han, B.K.; Yi, K. Serum Biomarkers for the Diagnosis of Glaucoma. *Diagnostics* **2020**, *11*, 20, doi:10.3390/diagnostics11010020.
140. Li, S.; Shao, M.; Li, Y.; Li, X.; Wan, Y.; Sun, X.; Cao, W. Relationship between Oxidative Stress Biomarkers and Visual Field Progression in Patients with Primary Angle Closure Glaucoma. *Oxid. Med. Cell. Longev.* **2020**, *2020*, doi:10.1155/2020/2701539.
141. Beutgen, V.M.; Schmelter, C.; Pfeiffer, N.; Grus, F.H. Autoantigens in the trabecular meshwork and glaucoma-specific alterations in the natural autoantibody repertoire. *Clin. Transl. Immunol.* **2020**, *9*, 1–20, doi:10.1002/cti2.1101.

142. Mirza, E.; Oltulu, R.; Katipoğlu, Z.; Mirza, G.D.; Özkağnıcı, A. Monocyte/HDL Ratio and Lymphocyte/Monocyte Ratio in Patients with Pseudoexfoliation Syndrome. *Ocul. Immunol. Inflamm.* **2020**, *28*, 142–146, doi:10.1080/09273948.2018.1545913.
143. Kondkar, A.A.; Azad, T.A.; Sultan, T.; Osman, E.A.; Almobarak, F.A.; Al-Obeidan, S.A. Elevated Plasma Level of 8-Hydroxy-2′-deoxyguanosine Is Associated with Primary Open-Angle Glaucoma. *J. Ophthalmol.* **2020**, *2020*, doi:10.1155/2020/6571413.
144. Gulpamuk, B.; Elgin, U.; Sen, E.; Yilmazbas, P.; Neselioglu, S.; Erel, O. Evaluation of dynamic thiol–disulfide homeostasis in glaucoma patients and the correlation with retinal nerve fiber layer analysis. *Eur. J. Ophthalmol.* **2020**, *30*, 690–699, doi:10.1177/1120672119839582.
145. Zhang, A.; Ning, L.; Han, J.; Ma, Y.; Ma, Y.; Cao, W.; Sun, X.; Li, S. Neutrophil-To-Lymphocyte Ratio as a Potential Biomarker of Neovascular Glaucoma. *Ocul. Immunol. Inflamm.* **2021**, *29*, 417–424, doi:10.1080/09273948.2019.1677916.
146. Ren, H.; Magulike, N.; Ghebremeskel, K.; Crawford, M. Primary open-angle glaucoma patients have reduced levels of blood docosahexaenoic and eicosapentaenoic acids. *Prostaglandins Leukot. Essent. Fat. Acids* **2006**, *74*, 157–163, doi:10.1016/j.plefa.2005.11.007.
147. Fraenkl, S.A.; Muser, J.; Groell, R.; Reinhard, G.; Orgul, S.; Flammer, J.; Goldblum, D. Plasma citrate levels as a potential biomarker for glaucoma. *J. Ocul. Pharmacol. Ther. Off. J. Assoc. Ocul. Pharmacol. Ther.* **2011**, *27*, 577–580, doi:10.1089/jop.2011.0062.
148. Tranchina, L.; Centofanti, M.; Oddone, F.; Tanga, L.; Roberti, G.; Liberatoscioli, L.; Cortese, C.; Manni, G. Levels of plasma homocysteine in pseudoexfoliation glaucoma. *Graefe's Arch. Clin. Exp. Ophthalmol.* **2011**, *249*, 443–448, doi:10.1007/s00417-010-1487-6.
149. Javadiyan, S.; Burdon, K.P.; Whiting, M.J.; Abhary, S.; Straga, T.; Hewitt, A.W.; Mills, R.A.; Craig, J.E. Elevation of serum asymmetrical and symmetrical dimethylarginine in patients with advanced glaucoma. *Invest. Ophthalmol. Vis. Sci.* **2012**, *53*, 1923–1927, doi:10.1167/iovs.11-8420.
150. Burgess, L.G.; Uppal, K.; Walker, D.I.; Roberson, R.M.; Tran, V.L.; Parks, M.B.; Wade, E.A.; May, A.T.; Umfress, A.C.; Jarrell, K.L.; et al. Metabolome-wide association study of primary open angle glaucoma. *Investig. Ophthalmol. Vis. Sci.* **2015**, *56*, 5020–5028, doi:10.1167/iovs.15-16702.
151. Michalczuk, M.; Tadeusz, P.; Urban, B.; Anna, W.; Bakunowicz-Łazarczyk, A. Plasma citrate concentration: A possible biomarker for glaucoma in children. *BMJ Paediatr. Open* **2017**, *1*, 1–5, doi:10.1136/bmjpo-2017-000023.
152. Rong, S.; Li, Y.; Guan, Y.; Zhu, L.; Zhou, Q.; Gao, M.; Pan, H.; Zou, L.; Chang, D. Long-chain unsaturated fatty acids as possible important metabolites for primary angle-closure glaucoma based on targeted metabolomic analysis. *Biomed. Chromatogr.* **2017**, *31*, doi:10.1002/bmc.3963.
153. Leruez S, Marill A, Bresson T, de Saint Martin G, Buisset A, Muller J, Tessier L, Gadras C, Verny C, Gohier P, Amati-Bonneau P, Lenaers G, Bonneau D, Simard G, Milea D, Procaccio V, Reynier P, Chao de la Barca JM. Leruez S, Marill A, Bresson T, de Saint M, C. de la B.J. A Metabolomics Profiling of Glaucoma Points to Mitochondrial Dysfunction, Senescence, and Polyamines Deficiency. *Invest. Ophthalmol. Vis. Sci.* **2018**, *59*, 4355–4361, doi:10.1167/iovs.18-24938.
154. Vohra, R.; Dalgaard, L.M.; Vibæk, J.; Langbøl, M.A.; Bergersen, L.H.; Olsen, N.V.; Hassel, B.; Chaudhry, F.A.; Kolko, M. Potential metabolic markers in glaucoma and their regulation in response to hypoxia. *Acta Ophthalmol.* **2019**, *97*, 567–576, doi:10.1111/aos.14021.
155. Umeno, A.; Tanito, M.; Kaidzu, S.; Takai, Y.; Horie, M.; Yoshida, Y. Comprehensive measurements of hydroxylinoleate and hydroxyarachidonate isomers in blood samples from primary open-angle glaucoma patients and controls. *Sci. Rep.* **2019**, *9*, 1–11, doi:10.1038/s41598-018-36952-6.
156. Li, S.; Shao, M.; Li, D.; Tang, B.; Cao, W.; Sun, X. Association of serum uric acid levels with primary open-angle glaucoma: a 5-year case–control study. *Acta Ophthalmol.* **2019**, *97*, e356–e363, doi:10.1111/aos.13789.

157. Lin, Z.; Huang, S.; Yu, H.; Sun, J.; Huang, P.; Zhong, Y. Analysis of plasma hydrogen sulfide, homocysteine, and l-cysteine in open-angle glaucoma patients. *J. Ocul. Pharmacol. Ther.* **2020**, *36*, 649–657, doi:10.1089/jop.2020.0023.
158. Nzoughet, J.K.; Guehlouz, K.; Leruez, S.; Gohier, P.; Bocca, C.; Muller, J.; Blanchet, O.; Bonneau, D.; Simard, G.; Milea, D.; et al. A data mining metabolomics exploration of glaucoma. *Metabolites* **2020**, *10*, doi:10.3390/metabo10020049.
